# Case Definitions and Data Sources for NNCSS Parkinson Disease Surveillance: A Systematic Review

**DOI:** 10.1001/jamanetworkopen.2026.13928

**Published:** 2026-06-08

**Authors:** Christine D. Esper, Kelly N. Hogan, Purni Abeysekara, Kasey Nichole Smith, Tenbroeck G. Smith, Shawna L. Mercer

**Affiliations:** 1 Department of Neurology, Emory University School of Medicine, Atlanta, Georgia; 2 National Neurological Conditions Surveillance System, Inform and Disseminate Division, Office of Public Health Data, Science, and Technology, Centers for Disease Control and Prevention (CDC), Department of Health and Human Services; 3 Guidehouse Inc, Chamblee, Georgia; 4 Alaka’ina Foundation Inc, Orlando, Florida; 5 4ES, San Antonio, Texas

## Abstract

**Importance:**

The National Neurological Conditions Surveillance System (NNCSS) is congressionally mandated to track the epidemiology of neurological conditions in the United States. Parkinson disease (PD) was selected as an initial condition, but methods for conducting PD surveillance are limited.

**Objective:**

To review evidence for, evaluate the performance of, and select data source types and case definitions for national PD surveillance.

**Evidence Review:**

Medline, Embase, PsycInfo, CINAHL, and Scopus were searched from January 1980 through November 2023; expert-identified and bibliographically mined references were included. Articles were screened and reviewed for eligibility from April 2020 to May 2024. Eligible articles included case definitions to produce population-based estimates and used data sources appropriate for population-level analyses. Study quality was assessed using Methodological Evaluation of Observational Research and Quality Assessment of Diagnostic Accuracy Studies–2. Case definitions and data source types were evaluated using key attributes of public health surveillance systems (hereafter key attributes).

**Findings:**

A total of 156 articles, published between 2001 and 2023, were included. These articles largely used administrative data (140 [90%]), especially claims (102 [65%]). The 163 included case definitions varied substantially and were allocated into 6 groups along a spectrum according to likely inclusion and/or exclusion of other parkinsonisms (OP) and resulting misclassification bias. At one end, “PD probable” definitions (15 of 163 [9%]) removed most OP, maximizing specificity but likely missing some PD cases with limited or conflicting claims evidence. Alternatively, “PD possible” definitions (11 of 163 [7%]) captured PD cases with limited or conflicting evidence, maximizing sensitivity but likely including some OP. Case definitions’ performance on key attributes varied widely. Claims performed best on key attributes.

**Conclusions and Relevance:**

In this systematic review of PD case definitions for national surveillance, no single definition provided a precise estimate due to potential diagnostic uncertainty in PD’s early stages and *International Classification of Disease *(*ICD*) coding limitations that preclude definitively distinguishing PD from OP in population-based data sources. The best-performing PD probable and PD possible case definitions were selected to use to establish an estimate range. The probable definition provides high diagnostic certainty of PD. The possible definition avoids missing PD cases with limited or conflicting evidence. This work demonstrated NNCSS’s methods for selecting and reappraising case definitions and data source types, providing a foundation for NNCSS’s PD surveillance and expansion to other neurological conditions.

## Key Points

**Question:**

What are the most suitable methods for national Parkinson disease (PD) surveillance in the United States via the National Neurological Conditions Surveillance System (NNCSS)?

**Findings:**

This systematic review of 156 articles found that case definitions varied widely due to the PD diagnostic process involving differentiating PD from other parkinsonisms and *International Classification of Disease *(*ICD*) code challenges. Evaluation identified claims as the most appropriate data source type, and 2 case definitions, one prioritizing sensitivity and the other specificity, were selected to produce an estimate range, preventing a false sense of precision.

**Meaning:**

These findings suggest foundational components for NNCSS’s national PD surveillance and expansion to other neurological conditions.

## Teaser

This systematic review evaluates data sources and case definitions in order for the National Neurological Conditions Surveillance System (NNCSS) to conduct surveillance on the incidence of Parkinson disease.

## Introduction

Parkinson disease (PD) is one of the most common neurodegenerative conditions and a substantial source of disability,^1,2^ contributing a significant burden to the patient, caregiver, and health care system.^3,4^ Diagnosis is typically based on a thorough history and clinical examination but can be challenging, as clinical features may overlap with other parkinsonisms (OP), particularly earlier in the disease course.^5^ While diagnostic accuracy generally increases with time,^1^ due to PD’s complexity and diagnostic challenges, as many as 20% of PD diagnoses may be incorrect.^6,7^

One 2025 study^8^ estimated nearly 700 000 US residents are living with PD, with prevalence higher among males and increasing by age. Other US estimates are limited, and no US national public health surveillance system for PD exists. Producing robust PD estimates and tracking national trends over time are important for identifying research priorities and informing action on risk factors, treatment, and support of patients and caregivers. The absence of situational awareness for PD and other neurological conditions led Congress to authorize the National Neurological Conditions Surveillance System (NNCSS), charging the US Centers for Disease Control and Prevention (CDC) with surveilling neurological conditions to facilitate further research.^9^ PD’s substantial burden led to its selection as one of NNCSS’s first conditions. The study’s aims were (1) to systematically evaluate and select case definitions for NNCSS’s PD surveillance and (2) to use standardized criteria to assess data source types available within or accessible to CDC to determine the most appropriate for national neurological condition surveillance.

## Methods

### Systematic Review

For this systematic review, we followed the Preferred Reporting Items for Systematic Reviews and Meta-Analyses (PRISMA) reporting guideline.^10^ Medline, Embase, PsycInfo, CINAHL, Scopus, and study references were searched for English-language studies about PD case definitions and population-based data sources, published January 1980 to November 2023 (eTable 1 in Supplement 1).

Two reviewers (C.D.E., K.N.H., P.A., K.N.S., and T.G.S.) independently screened studies from April 2020 to May 2024 using Covidence.^11^ A third reviewer (K.N.H.) resolved disagreements. Inclusion and exclusion criteria focused on data sources and case definitions useful for population-based analyses (eMethods 1 in Supplement 1). Reviewers abstracted study characteristics, data sources, and case definitions.

Two reviewers (K.N.S. and T.G.S.) independently assessed study quality (eMethods 2 in Supplement 1) using the Quality Assessment of Diagnostic Accuracy Studies–2 (QUADAS-2)^12^ for studies validating case definitions (hereafter validation studies) and Methodological Evaluation of Observational Research^13^ for remaining studies (hereafter nonvalidation studies). Interrater reliability (IRR) was calculated. Two reviewers (K.N.S. and T.G.S.) resolved discrepancies to reach consensus. A third reviewer (K.N.H.) resolved disagreements.

### Data Analysis

We developed a multistage, iterative process for case definition evaluation and selection (Figure). We first assessed case definitions for suitability. To understand case definitions’ risk of bias (RoB) from misclassification, we identified which, if any, other conditions could act as a source of potential misclassification and compared their characteristics with PD based on findings from published literature (ie, morbidity, mortality, demographics, and disease burden). Those conditions that, if included in a PD case definition, would likely overestimate, underestimate, or otherwise skew PD measures were considered to present RoB from misclassification. Based on this, we determined the risk of misclassification^14,15,16,17^ from each source and assessed the likely impact on each case definition. Finally, we evaluated case definitions for performance on key attributes of public health surveillance systems (hereafter key attributes) (eTable 2 in Supplement 1),^18^ and those that best maximized and balanced the key attributes were selected.

**Figure.**
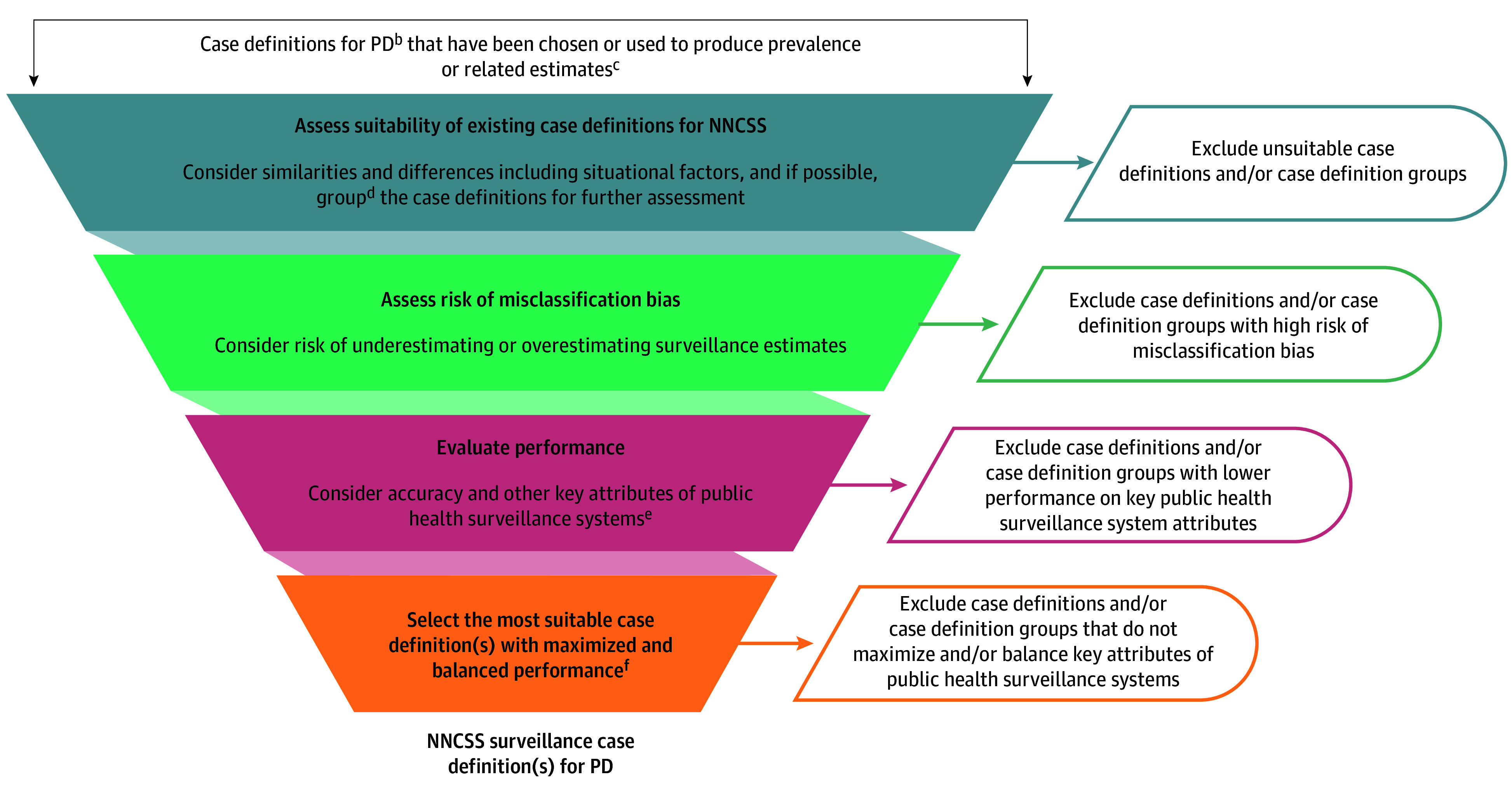
National Neurological Conditions Surveillance System (NNCSS) Case Definition Selection Process for Parkinson Disease (PD)^a^ ^a^This process was iterative; findings from steps may inform the need to return to prior steps, resulting in reassessment of the case definitions at prior stages in the process. ^b^Also referred to in the literature as case algorithms or case identification algorithms. ^c^Related estimates include estimates such as incidence, health care and medication use, cost, and risk factors, among others. ^d^Grouping can be performed by data source type, consistency with current diagnostic and/or treatment standards, and parameters used, among other considerations. ^e^The key attributes of public health surveillance systems are adapted and extended from Groseclose and Buckeridge^18^ and include accuracy (ie, sensitivity, specificity, positive predictive value, and negative predictive value), simplicity, acceptability, cost-effectiveness, reproducibility, and scalability/spreadability. ^f^If no case definition options remain, return to step 1 and reassess or create a new surveillance case definition using evidence from systematic review.

We reviewed search results to identify data source types used in the literature. Among data source types accessible to CDC, we considered their performance on key attributes^18,19^ and compared them to identify the most appropriate for NNCSS.

## Results

### Search Results

In total, we identified and screened 7954 citations; 156 studies met eligibility criteria (eFigure in Supplement 1) including 23 validation^20,21,22,23,24,25,26,27,28,29,30,31,32,33,34,35,36,37,38,39,40,41,42^ and 133 nonvalidation studies.^43,44,45,46,47,48,49,50,51,52,53,54,55,56,57,58,59,60,61,62,63,64,65,66,67,68,69,70,71,72,73,74,75,76,77,78,79,80,81,82,83,84,85,86,87,88,89,90,91,92,93,94,95,96,97,98,99,100,101,102,103,104,105,106,107,108,109,110,111,112,113,114,115,116,117,118,119,120,121,122,123,124,125,126,127,128,129,130,131,132,133,134,135,136,137,138,139,140,141,142,143,144,145,146,147,148,149,150,151,152,153,154,155,156,157,158,159,160,161,162,163,164,165,166,167,168,169,170,171,172,173,174,175^ Nearly two-thirds (100 [64%]) were published between 2016 and 2023^25,27,28,29,31,32,35,37,38,41,43,45,49,51,53,54,56,57,58,59,60,61,62,65,66,67,68,69,70,71,72,74,76,77,78,80,81,84,85,88,89,90,91,94,95,96,97,102,103,107,108,111,112,114,115,116,117,119,120,121,122,123,124,125,127,128,130,132,133,135,136,137,138,139,140,141,142,143,144,146,147,149,150,151,152,153,156,159,160,164,165,166,167,170,171,172,173,174,175^ (Table 1; eTable 3 in Supplement 1). Nearly all (140 [90%]) utilized administrative data^20,21,22,23,24,27,28,30,31,32,33,34,35,36,39,40,41,42,43,44,46,47,48,50,51,52,53,54,55,56,57,58,59,60,62,63,64,65,66,67,68,69,70,71,72,73,74,76,78,79,80,81,82,83,85,86,87,88,89,90,91,92,93,94,95,96,97,98,99,100,101,102,103,104,105,106,107,108,109,110,111,112,113,114,116,117,119,120,121,122,123,124,125,126,127,128,129,130,131,132,133,134,135,137,138,139,140,141,143,144,145,146,147,148,149,150,151,152,153,154,155,156,157,158,159,160,161,162,163,164,165,166,167,168,169,171,172,174,175^ (102 [65%] used claims^20,21,22,27,28,30,35,41,43,48,50,53,54,56,57,58,59,60,62,63,64,65,66,67,68,69,70,71,72,73,74,76,78,79,80,81,82,83,85,86,87,88,89,90,91,93,94,95,96,97,100,101,102,103,104,106,107,108,109,110,111,112,113,114,119,120,121,122,123,124,125,127,128,129,130,131,132,133,134,135,137,141,143,145,146,149,150,151,153,155,159,160,161,162,163,166,167,168,169,171,172,174,175^); few used EHR^25,37,45,75,77,84,142^ or survey data.^26,49,61,118,155,170^ Just under half of the included studies (66 [42%]) were US-based.^25,26,29,30,31,32,33,34,36,37,38,39,42,48,49,55,56,58,59,61,62,73,75,77,78,79,80,84,86,89,91,94,95,96,97,98,99,104,106,109,112,113,115,116,118,119,128,131,132,141,145,149,153,154,155,157,162,163,165,167,168,169,170,173,174,175^ The most-used US data sources were Medicare (26 [39%]),^26,29,30,56,62,73,78,80,89,91,94,95,96,97,113,119,132,141,149,153,155,162,163,167,168,169,174,175^ MarketScan (6 [9%]),^58,94,106,112,128,131^ and Veterans Health Administration (5 [8%]).^32,33,34,36,165^

**Table 1.** Characteristics of Included Studies

Characteristic	No. (%) (N = 156)
Publication year	
1980-2000	0
2001-2005	5 (3)
2006-2010	17 (11)
2011-2015	34 (22)
2016-2020	67 (43)
2021-2023	33 (21)
Country	
United States	65 (42)
United States and international	1 (<1)
International	90 (58)
Data source type^a^	
Administrative	140 (90)
Claims	102 (65)
Other	55 (35)
Health care records or utilization information for nonbilling purposes	41 (26)
Hospitalization or hospital discharge	17 (11)
Other	6 (4)
Facility-reported	3 (2)
EHR	7 (4)
EHR linked with administrative (claims or other) data	3 (2)
Survey	6 (4)
Other	2 (1)

Abbreviation: EHR, electronic health records or electronic medical records.

^a^ Not mutually exclusive; some studies used more than 1 data source type.

Validation study quality assessment IRR ranged from 33.3% to 88.9% (eTable 4 in Supplement 1). The most common concern was patient selection causing low applicability (73.9%) (eTable 5 in Supplement 1). Nonvalidation study quality assessment IRR ranged from 55.8% to 100% (eTable 6 in Supplement 1). The most common concern was RoB due to case identification method (94.0%) (eTable 7 in Supplement 1).

### Case Definition Suitability

We abstracted 163 distinct case definitions selected by authors from included studies: 25 from validation studies and 138 from nonvalidation studies. Situational factors (eg, frequency of use or citation, study purpose) did not adequately differentiate case definitions (eTable 8 in Supplement 1).

Across case definitions, 4 parameters were commonly used alone or in combination to include cases and/or exclude noncases: (1) diagnosis, including setting (eg, inpatient, outpatient) and *International Classification of Disease *(*ICD*) or other code; (2) pharmaceutical claim; (3) provider (ie, individual practitioners, organizational entities, or facilities/suppliers providing medical services) specialty; and 4) time, including time required between parameters and within which parameters must be met. Case definitions varied the most on the diagnosis parameter due to several factors. First, challenges with *ICD* codes^176,177^ codes (eTable 9 in Supplement 1) exist due to overlap in *ICD* code definitions for PD and OP. For example, the *International Statistical Classification of Diseases and Related Health Problems, Tenth Revision *(*ICD-10*) code for PD is G20, but based on its definition, it also includes OP. Therefore, OP, such as progressive supranuclear palsy (PSP), which has the specific *ICD-10* code of G23.1, could be correctly coded as G20 or G23.1. Second, disease course may affect the PD diagnostic process, particularly early on, as response to dopaminergic medications and/or presence of red flags^178^ (ie, atypical clinical history or signs) may be unclear, potentially resulting in diagnosis changes. Third, a change in diagnosis may not be reflected in *ICD* codes on diagnostic claims given the aforementioned code definition overlap. Finally, coding practices vary, particularly the use of G20 for PD and OP, despite availability of more specific codes for OP (eTable 9 in Supplement 1).^179,180^

Examination of case definitions indicated that to get as close as possible to capturing only PD cases, a case definition must use multiple parameters to confirm the PD diagnosis and as well as additional parameters to exclude OP. Case definitions differed substantially in their use of parameter combinations to reduce inclusion of OP, creating considerable variability in the type and proportion of OP likely captured. Among studies explicitly describing case definitions as removing or capturing OP (eTable 3 in Supplement 1), the most frequently referenced conditions were PSP, multiple system atrophy (MSA), corticobasal degeneration/syndrome (CBD/CBS), dementia with Lewy Bodies (DLB), drug-induced parkinsonism (DIP), and vascular parkinsonism (VasP).

### Risk of Misclassification Bias

We compared characteristics of these OP with PD to understand the risk of misclassification in case definitions. Ultimately, we categorized OP into 2 groups based on their RoB. The OP posing the biggest RoB to PD surveillance estimates were DIP, VasP, and DLB. For example, because the burden of these conditions by sex differs from PD, their inclusion would likely skew PD surveillance estimates by sex.^1,132,181,182,183,184,185,186,187^ The OP posing some RoB were MSA, PSP, and CBD/CBS. While less common than PD, their characteristics could introduce bias in PD surveillance estimates. For example, their survival time is shorter than that of PD, and inclusion would likely underestimate PD survival time.^188,189,190^ Overall, these findings suggest OP included in PD case definitions could introduce meaningful misclassification bias and are best surveilled as separate conditions.

We assigned case definitions into groups A to E according to the type and proportion of OP they captured (Table 2). The groups are bracketed by a theoretical group that would remove all OP (not currently possible due to coding and disease progression challenges) and a group that would include all OP (not assessed as outside this review’s scope). Case definitions were distributed across groups, from group A, which included parameter combinations that removed OP posing the biggest RoB and at least some OP posing some RoB, to group E, which included OP posing the biggest RoB and at least some OP posing some RoB. An additional drug tracer group, which used detailed longitudinal pharmaceutical data, was not feasible for NNCSS within currently available data source types.

**Table 2.** Case Definition Groupings Based on Risk of Misclassification Bias to Parkinson Disease Surveillance Estimates From Other Parkinsonisms

Group^b^	Group criteria^c^	Misclassification bias attributed to^a^		Case definitions evaluated, No. (%) (n = 153)
		OP with the biggest risk^d^	OP with some risk^e^	
Theoretical	Currently not possible^f^; confirms probable PD diagnosis; removes all OP	None	None	NA
A	Confirms a probable PD diagnosis and removes most OP	Low	Low to moderate	15 (10)
B	May confirm a probable PD diagnosis and removes some OP	Moderate	Moderate	47 (31)
C	Identifies PD (does not confirm diagnosis) and does not remove or add OP^g^	High	High	47 (31)
D	May or may not confirm diagnosis, but includes PD as well as some OP	High to definite	High	33 (22)
E	May or may not confirm diagnosis, but includes PD as well as many OP	High to definite	High to definite	11 (7)
Unassessed group^h^	May or may not confirm diagnosis, but includes PD and all OP	Definite	Definite	NA

Abbreviations: NA, not applicable; OP, other parkinsonisms; PD, Parkinson disease.

^a^ When case definitions include PD and OP, they introduce misclassification bias to PD estimates generated using those case definitions. We assessed each OP for the risk of bias it could introduce if included in a PD case definition by comparing it to PD on characteristics such as morbidity, mortality, subgroup characteristics, and disease burden.

^b^ One additional group of 10 drug tracer case definitions was not included in this table because they are not feasible with data source types currently available to the US Centers for Disease Control and Prevention, which are limited on length of longitudinal data available, detailed information on prescriptions, and patient-specific information such as risk factors.

^c^ Group A and B definitions use multiple parameters to confirm a PD diagnosis (eg, require more than 1 inpatient or outpatient diagnosis with an associated PD *International Classification of Diseases* [*ICD*] code). Group C definitions do not confirm a PD diagnosis. Groups D and E may or may not confirm a PD diagnosis, but the distinguishing feature of these case definitions is the inclusion of some OP conditions.

^d^ OP with the biggest risk of misclassification bias are dementia with Lewy Bodies, vascular parkinsonism, and drug-induced parkinsonism.

^e^ OP with some risk of misclassification bias are progressive supranuclear palsy, multiple system atrophy, and corticobasal degeneration/syndrome.

^f^ Not currently possible due to current *ICD* code definitions, coding practices, and potential ambiguity of diagnosis in early stages of PD.

^g^ Secondary parkinsonisms must be removed in case definitions utilizing certain *ICD*, *Ninth Edition*, codes.

^h^ Would require using case definitions for all 6 OP in addition to the PD case definition, which is beyond the scope of this study and was therefore not assessed.

Groups A and E reflect contrasting approaches to PD case definitions. Group A definitions (15 of 163 [9%]) use a “PD probable” approach, which prioritizes specificity (ie, minimizes false positives) and identifies cases with claims evidence indicating high diagnostic certainty of PD. This minimizes overestimation of PD cases due to misclassification of OP as PD; however, it may miss some cases early in their diagnostic process with incomplete evidence of PD or whose PD diagnosis is unclear due to coding challenges, although this number is likely relatively small.

Group E definitions (11 of 163 [7%]) use a “PD possible” approach, which prioritizes sensitivity (ie, minimizes false negatives) and includes cases in the PD diagnostic process gray area where there is potential ruling in or out of PD and OP based on the presence of red flags and/or cases whose coding could be consistent with PD or OP. This increases the chance of misclassification bias because these definitions capture some patients who may ultimately be diagnosed with an OP rather than PD.

Given the inherent limitations of identifying PD in claims data and the complex diagnostic process, producing estimates based on a single case definition would convey a misleading sense of precision. Instead, we selected 2 PD case definitions to use to establish an estimate range: one probable case definition (group A) to capture more definitive cases and one possible case definition (group E) to capture the full extent of potential cases.

### Performance on Key Attributes of Public Health Surveillance Systems

Next, we evaluated the performance of group A and E case definitions through an assessment of measures of accuracy, when provided, and other key attributes (Table 3; eTable 10 in Supplement 1). We compared measures of accuracy provided by authors who validated their case definitions against a reference standard. Using our expertise in epidemiology, surveillance, and movement disorders, we evaluated the other key attributes using the definitions and criteria identified by Groseclose et al^18^ and considered relevant information provided on other key attributes in the reviewed articles. Each case definition was assessed relative to each other and rated on the other key attributes using a scale of 1 (lowest performance) to 6 (highest performance).

**Table 3.** Evaluation of Group A and E Case Definitions on Key Attributes of Public Health Surveillance Systems by Case Definition Group and Data Source Type—Part A: Measures of Accuracy^a^^,^^b^^,^^c^

Source	Case definition	*ICD *code	Reference standard		% (95% CI)			
			PD or PD with OP^d^	Source	Sensitivity	Specificity	PPV	NPV
**Group A**								
Data source type: claims								
Lee et al,^27^ 2016^e^	Inclusions: ≥2 inpatient or outpatient diagnosis (first and last diagnosis ≥90 d apart) and ≥3 PD pharmaceutical claims after index PD diagnosis (all in 3 y) Exclusions: secondary parkinsonism diagnosis (*ICD-9 *code 332.1) at any time, or any neuroleptic pharmaceutical claim within 180 d prior to first PD diagnosis, or dementia diagnosis (*ICD-9 *codes 290 or 331) prior to first PD diagnosis	*ICD-9 *code 332.0	PD	PD cohort^f^	97.6 (NR)	92.3 (NR)	98.8 (NR)	85.7 (NR)
Liu et al,^28^ 2016^e^	Inclusions: ≥3 inpatient or outpatient diagnosis and ≥3 PD pharmaceutical claims after first diagnosis in 7 y and first and last inpatient/outpatient diagnosis separated by ≥90 d Exclusions: secondary parkinsonism diagnosis (*ICD-9 *code 332.1) at any time, or any neuroleptic pharmaceutical claim within 180 d prior to first PD diagnosis or ≥3 inpatient, or outpatient diagnosis for dementia (*ICD-9 *codes 290 or 331) prior to first PD diagnosis	*ICD-9 *code 332.0	PD	PD cohort^f^	97.6 (NR)	92.3 (NR)	98.8 (NR)	85.7 (NR)
Szatmari et al,^35^ 2019^e^	Inclusions: ≥2 inpatient or outpatient diagnosis (1 diagnosis in ≥2 of 10 y) Exclusions: diagnosis with *ICD-10 *codes G21-G26 at any time	*ICD-10* code G20	PD	MRR^g^	NR (NR)	NR (NR)	88.0 (NR)	NR (NR)
Data source type: other administrative^h^								
Szumski et al,^36^ 2009^e^	Inclusions: ≥2 outpatient diagnosis (if most used diagnosis from highest specialist) Exclusions: any other code more diagnosed by the highest specialist on record or only 1 diagnosis of *ICD-9 *code 332.0	*ICD-9 *code 332.0	PD	MRR^g^	87.4 (83.9-90.4)	45.4 (37.0-54.0)	83.2 (79.4-86.5)	53.8 (44.4-63.0)
Szumski et al,^36^ 2009^i^	Inclusions: ≥2 outpatient diagnosis (if most used diagnosis from highest specialist) and ≥1 pharmaceutical claim Exclusions: any other code more diagnosed by the highest specialist on record or only 1 diagnosis of *ICD-9 *code 332.0	*ICD-9 *code 332.0	PD	MRR^g^	77.1 (72.8-80.9)	68.1 (59.7-75.7)	88.2 (84.5-91.3)	49.0 (41.8-56.2)
**Group E^j^**								
Data source type: claims								
Noyes et al,^30^ 2007^i^	Inclusions: ≥1 diagnosis (PHY only)	*ICD-9 *codes 332.0, 332.1, 333.0, or 333.1	PD and OP	MCBS and pharmaceutical claims^k^	52.5 (NR)	99.3 (NR)	67.2 (NR)	NR (NR)
Noyes et al,^30^ 2007^i^	Inclusions: ≥1 diagnosis (in any claims)	*ICD-9 *codes 332.0, 332.1, 333.0, or 333.1	PD and OP	MCBS and pharmaceutical claims^k^	61.1 (NR)	99.1 (NR)	65.1 (NR)	NR (NR)
Noyes et al,^30^ 2007^i^	Inclusions: ≥1 diagnosis (PHY only)	*ICD-9 *codes 332.0, 332.1, 333.0, or 333.1	PD and OP	MCBS^l^	57.0 (NR)	99.2 (NR)	62.3 (NR)	NR (NR)
Noyes et al,^30^ 2007^i^	Inclusions: ≥1 diagnosis (in any claims)	*ICD-9 *codes 332.0, 332.1, 333.0, or 333.1	PD and OP	MCBS^l^	66.1 (NR)	99.0 (NR)	60.1 (NR)	NR (NR)
Data source type: other administrative								
Feldman et al,^23^ 2012^i^	Inclusions: ≥1 inpatient diagnosis	*ICD-9 *codes 332.0 or 333.0; *ICD-10* codes G20, G21.4, G21.8-G21.9, G23.1-G23.2, G23.9, or G25.9; or *ICD 7-8* codes	PD and OP	SALT study^m^	63.4 (56.2-70.2)	NR	88.0 (78.4-94.4)	NR
Swarztrauber et al,^33^ 2005^i^	Inclusions: ≥1 inpatient or outpatient diagnosis	*ICD-9 *codes 332.0, 332.1, or 333.0	PD and OP	MRR^g^	18.7 (8.1-29.3)	99.9 (99.9-99.9)	81.0 (75.4-86.7)	NR
Swarztrauber et al,^33^ 2005^i^	Inclusions: (≥1 inpatient or outpatient diagnosis) or (≥1 pharmaceutical claim)	*ICD-9 *codes 332.0, 332.1, 333.0, or 781.0	PD and OP	MRR^g^	42.5 (18.3-66.7)	99.3 (99.2-99.4)	53.3 (46.5-60.1)	NR
Swarztrauber et al,^33^ 2005^i^	Inclusions: 1 pharmaceutical claim	NU	PD and OP	MRR^g^	34.6 (14.3-54.9)	99.6 (99.5-99.7)	60.9 (56.9-64.9)	NR
Swarztrauber et al,^33^ 2005^e^	Inclusions: (≥1 inpatient or outpatient diagnosis) or (≥1 pharmaceutical claim)	*ICD-9 *codes 332.0, 332.1, or 333.0	PD and OP	MRR^g^	37.9 (16.3-59.5)	99.5 (99.4-99.6)	60.4 (52.0-68.8)	NR
Szumski et al,^36^ 2009^e^	Inclusions: 1 pharmaceutical claim	NU	PD	MRR^g^	80.0 (76-83.7)	58.2 (49.6-66.4)	85.5 (81.8-88.8)	48.5 (40.8-56.3)
Data source type: claims and other administrative								
Baldacci et al,^20^ 2015^e^	Inclusions: ≥1 of the following: ≥1 inpatient PD diagnosis, PD exemption, or ≥2 PD pharmaceutical claim dispensed in 1 y ≥6 mo apart	*ICD-9 *code 332	PD	PD cohort^f^	91.2 (NR)	NR	NR	NR
Butt et al,^21^ 2014^i^^,^^n^	Inclusions: 1 pharmaceutical claim	NU	PD and OP	MRR^g^	71.0 (65.1-76.8)	99.6 (99.5-99.6)	35.7 (31.3-40.1)	99.9 (99.9-99.9)
Butt et al,^21^ 2014^e^^,^^n^^,^^o^	Inclusions: (≥2 outpatient diagnosis ≥30 d apart in 1 y) or (1 pharmaceutical claim and 1 outpatient diagnosis 6 mo before or after pharmaceutical claim)	*ICD-9 *codes 332, 332.0, or 332.1; or *ICD-10* codes G20, G21.0-G21.4, G21.8-G21.9, G22, or F02.3	PD and OP	MRR^g^	77.8 (72.6-83.3)	99.9 (99.9-99.9)	76.9 (71.5-82.3)	99.9 (99.9-99.9)
Butt et al,^21^ 2014^e^^,^^n^^,^^o^	Inclusions: 2 outpatient diagnosis ≥30 d apart in 1 y	*ICD-9 *codes 332, 332.0, or 332.1; or *ICD-10* codes G20, G21.0-G21.4, G21.8-G21.9, G22, or F02.3	PD and OP	MRR^g^	70.6 (64.7-76.4)	99.9 (99.9-100)	79.5 (74.0-85.0)	99.9 (99.9-99.9)
Butt et al,^21^ 2014^i^^,^^n^	Inclusions: 1 outpatient diagnosis or 1 pharmaceutical claim	*ICD-9 *codes 332, 332.0, or 332.1; or *ICD-10* codes G20, G21.0-G21.4, G21.8-G21.9, G22, or F02.3	PD and OP	MRR^g^	87.0 (82.7-91.3)	99.3 (99.2-99.4)	28.3 (25.0-31.6)	100 (99.9-100)
Butt et al,^21^ 2014^i^^,^^n^	Inclusions: 1 outpatient diagnosis and 1 pharmaceutical claim	*ICD-9 *codes 332, 332.0, or 332.1; or *ICD-10* codes G20, G21.0-G21.4, G21.8-G21.9, G22, or F02.3	PD and OP	MRR^g^	66.7 (60.6-72.7)	100 (99.9-100)	83.7 (78.4-89.0)	99.9 (99.9-99.9)
Butt et al,^21^ 2014^i^^,^^n^	Inclusions: 1 pharmaceutical claim and 1 outpatient diagnosis 6 mo before or after pharmaceutical claim	*ICD-9 *codes 332, 332.0, or 332.1; or *ICD-10* codes G20, G21.0-G21.4, G21.8-G21.9, G22, or F02.3	PD and OP	MRR^g^	65.8 (59.7-71.9)	100 (100-100)	86.4 (81.3-91.4)	99.9 (99.9-99.9)
Butt et al,^21^ 2014^i^^,^^n^	Inclusions: 1 outpatient diagnosis and 1 pharmaceutical claim, all in 1 y	*ICD-9 *codes 332, 332.0, or 332.1; or *ICD-10* codes G20, G21.0-G21.4, G21.8-G21.9, G22, or F02.3	PD and OP	MRR^g^	65.8 (59.7-71.9)	100 (100-100)	86.4 (81.3-91.4)	99.9 (99.9-99.9)
Butt et al,^21^ 2014^i^^,^^n^	Inclusions: 2 outpatient diagnosis ≥30 d apart and 1 pharmaceutical claim, all in 1 y	*ICD-9 *codes 332, 332.0, or 332.1; or *ICD-10* codes G20, G21.0-G21.4, G21.8-G21.9, G22, or F02.3	PD and OP	MRR^g^	61.0 (54.8-67.3)	100 (100-100)	89.2 (84.4-94.1)	99.9 (99.9-99.9)
Butt et al,^21^ 2014^i^^,^^n^	Inclusions: (≥2 outpatient diagnosis ≥30 d apart in 1 y) or (1 pharmaceutical claim and 1 outpatient diagnosis)	*ICD-9 *codes 332, 332.0, or 332.1; or *ICD-10* codes G20, G21.0-G21.4, G21.8-G21.9, G22, or F02.3	PD and OP	MRR^g^	78.4 (73.0-83.7)	99.9 (99.9-99.9)	75.4 (70.0-80.9)	99.9 (99.9-99.9)
Butt et al,^21^ 2014^i^^,^^n^	Inclusions: 1 pharmaceutical claim and 1 inpatient or outpatient diagnosis 6 mo before or after pharmaceutical claim	*ICD-9 *codes 332, 332.0, or 332.1; or *ICD-10* codes G20, G21.0-G21.4, G21.8-G21.9, G22, or F02.3	PD and OP	MRR^g^	66.2 (60.1-72.3)	100 (100-100)	86.4 (81.4-91.5)	99.9 (99.9-99.9)
Butt et al,^21^ 2014^i^^,^^n^	Inclusions: (1 inpatient diagnosis or 2 outpatient diagnosis ≥30 d apart in 1 y) or (1 pharmaceutical claim and 1 inpatient or outpatient diagnosis)	*ICD-9 *codes 332, 332.0, or 332.1; or *ICD-10* codes G20, G21.0-G21.4, G21.8-G21.9, G22, or F02.3	PD and OP	MRR^g^	78.4 (73.9-83.7)	99.9 (99.9-99.9)	70.7 (65.1-76.3)	99.9 (99.9-99.9)
Butt et al,^21^ 2014^i^^,^^n^	Inclusions: (1 inpatient diagnosis or 2 outpatient diagnosis ≥30 d apart in 1 y) or (1 pharmaceutical claim and 1 inpatient or outpatient diagnosis 6 mo before or after pharmaceutical claim)	*ICD-9 *codes 332, 332.0, or 332.1; or *ICD-10* codes G20, G21.0-G21.4, G21.8-G21.9, G22, or F02.3	PD and OP	MRR^g^	77.8 (72.6-83.3)	99.9 (99.9-99.9)	72.0 (66.4-77.6)	99.9 (99.9-99.9)
Butt et al,^21^ 2014^i^^,^^n^	Inclusions: 1 inpatient diagnosis	*ICD-9 *codes 332, 332.0, or 332.1; or *ICD-10* codes G20, G21.0-G21.4, G21.8-G21.9, G22, or F02.3	PD and OP	MRR^g^	26.0 (20.3-31.6)	100 (100-100)	75.9 (66.5-85.4)	99.8 (99.7-99.8)
Butt et al,^21^ 2014^i^^,^^n^	Inclusions: 1 inpatient or emergency department or same-day surgery diagnosis	*ICD-9 *codes 332, 332.0, or 332.1; or *ICD-10* codes G20, G21.0-G21.4, G21.8-G21.9, G22, or F02.3	PD and OP	MRR^g^	32.9 (26.8-39.0)	100 (100-100)	74.5 (66.1-83.0)	99.8 (99.8-99.8)
Butt et al,^21^ 2014^i^^,^^n^	Inclusions: 1 outpatient diagnosis	*ICD-9 *codes 332, 332.0, or 332.1; or *ICD-10* codes G20, G21.0-G21.4, G21.8-G21.9, G22, or F02.3	PD and OP	MRR^g^	82.7 (77.8-87.6)	99.7 (99.6-99.7	43.9 (39.2-48.6)	99.9 (99.9-99.9)
Butt et al,^21^ 2014^i^^,^^n^	Inclusions: 1 outpatient diagnosis (specialist only)	*ICD-9 *codes 332, 332.0, or 332.1; or *ICD-10* codes G20, G21.0-G21.4, G21.8-G21.9, G22, or F02.3	PD and OP	MRR^g^	69.3 (63.3-75.2)	99.8 (99.8-99.9)	56.1 (50.4-61.9)	99.9 (99.9-99.9)
Butt et al,^21^ 2014^i^^,^^n^	Inclusions: 2 outpatient diagnosis in 1 y	*ICD-9 *codes 332, 332.0, or 332.1; or *ICD-10* codes G20, G21.0-G21.4, G21.8-G21.9, G22, or F02.3	PD and OP	MRR^g^	73.2 (67.4-78.9)	99.9 (99.9-99.9)	72.8 (67.1-78.6)	99.9 (99.9-99.9)
Butt et al,^21^ 2014^i^^,^^n^	Inclusions: 2 outpatient diagnosis ≥30 d apart in 1 y (specialist only)	*ICD-9 *codes 332, 332.0, or 332.1; or *ICD-10* codes G20, G21.0-G21.4, G21.8-G21.9, G22, or F02.3	PD and OP	MRR^g^	58.4 (52.1-64.8)	100 (99.0-100)	83.3 (77.6-89.1)	99.9 (99.8-99.9)
Butt et al,^21^ 2014^i^^,^^n^	Inclusions: 2 outpatient diagnosis ≥30 d apart in 2 y	*ICD-9 *codes 332, 332.0, or 332.1; or *ICD-10* codes G20, G21.0-G21.4, G21.8-G21.9, G22, or F02.3	PD and OP	MRR^g^	71.9 (66.1-77.7)	99.9 (99.9-100)	77.2 (71.6-82.8)	99.9 (99.9-99.9)
Butt et al,^21^ 2014^i^^,^^n^	Inclusions: 2 outpatient diagnosis ≥30 d apart in 3 y	*ICD-9 *codes 332, 332.0, or 332.1; or *ICD-10* codes G20, G21.0-G21.4, G21.8-G21.9, G22, or F02.3	PD and OP	MRR^g^	72.3 (66.5-78.1)	99.9 (99.9-99.9)	75.9 (70.3-81.6)	99.9 (99.9-99.9)
Butt et al,^21^ 2014^i^^,^^n^	Inclusions: 3 outpatient diagnosis ≥30 d apart in 1 y	*ICD-9 *codes 332, 332.0, or 332.1; or *ICD-10* codes G20, G21.0-G21.4, G21.8-G21.9, G22, or F02.3	PD and OP	MRR^g^	58.9 (52.5-65.2)	100 (100-100)	88.3 (83.2-93.4)	99.9 (99.8-99.9)
Butt et al,^21^ 2014^i^^,^^n^	Inclusions: (1 inpatient diagnosis) or (2 outpatient diagnosis ≥30 d apart, all in 1 y)	*ICD-9 *codes 332, 332.0, or 332.1; or *ICD-10* codes G20, G21.0-G21.4, G21.8-G21.9, G22, or F02.3	PD and OP	MRR^g^	71.9 (66.1-77.7)	99.9 (99.9-99.9)	73.8 (68.0-79.5)	99.9 (99.9-99.9)
Butt et al,^21^ 2014^e^^,^^o^^,^^p^	Inclusions: 2 outpatient diagnosis ≥30 d apart in 1 y	*ICD-9 *codes 332, 332.0, or 332.1; or *ICD-10* codes G20, G21.0-G21.4, G21.8-G21.9, G22, or F02.3	PD and OP	MRR^g^	72.3 (67.0-79.6)	99.8 (99.7-99.9)	82.8 (77.1-88.5)	99.6 (99.5-99.7)
Butt et al,^21^ 2014^e^^,^^o^^,^^p^	Inclusions: 1 pharmaceutical claim and 1 outpatient diagnosis 6 mo before or after pharmaceutical claim	*ICD-9 *codes 332, 332.0, or 332.1; or *ICD-10* codes G20, G21.0-G21.4, G21.8-G21.9, G22, or F02.3	PD and OP	MRR^g^	75.9 (69.9-82.0)	99.9 (99.8-99.9)	87.3 (82.3-92.4)	99.7 (99.6-99.8)
Butt et al,^21^ 2014^i^^,^^p^	Inclusions: 1 inpatient diagnosis	*ICD-9 *codes 332, 332.0, or 332.1; or *ICD-10* codes G20, G21.0-G21.4, G21.8-G21.9, G22, or F02.3	PD and OP	MRR^g^	28.8 (22.4-35.2)	99.9 (99.9-100)	87.3 (79.1-95.5)	99.0 (98.9-99.2)
Butt et al,^21^ 2014^i^^,^^p^	Inclusions: 1 inpatient or emergency department or same-day surgery diagnosis	*ICD-9 *codes 332, 332.0, or 332.1; or *ICD-10* codes G20, G21.0-G21.4, G21.8-G21.9, G22, or F02.3	PD and OP	MRR^g^	35.1 (28.3-41.8)	99.9 (99.9-100)	83.8 (75.7-91.8)	99.1 (99.0-99.3)
Butt et al,^21^ 2014^i^^,^^p^	Inclusions: 1 outpatient diagnosis	*ICD-9 *codes 332, 332.0, or 332.1; or *ICD-10* codes G20, G21.0-G21.4, G21.8-G21.9, G22, or F02.3	PD and OP	MRR^g^	84.8 (79.7-89.9)	99.0 (98.9-99.2)	54.5 (48.9-60.2)	99.8 (99.7-99.9)
Butt et al,^21^ 2014^i^^,^^p^	Inclusions: 1 outpatient diagnosis (specialist only)	*ICD-9 *codes 332, 332.0, or 332.1; or *ICD-10* codes G20, G21.0-G21.4, G21.8-G21.9, G22, or F02.3	PD and OP	MRR^g^	70.7 (64.2-77.1)	99.6 (99.5-99.7)	68.5 (62.0-75.0)	99.6 (99.5-99.7)
Butt et al,^21^ 2014^i^^,^^p^	Inclusions: 2 outpatient diagnosis in 1 y	*ICD-9 *codes 332, 332.0, or 332.1; or *ICD-10* codes G20, G21.0-G21.4, G21.8-G21.9, G22, or F02.3	PD and OP	MRR^g^	75.4 (69.3-81.5)	99.7 (99.6-99.8)	76.6 (70.5-82.6)	99.7 (99.6-99.8)
Butt et al,^21^ 2014^i^^,^^p^	Inclusions: 2 outpatient diagnosis ≥30 d apart in 1 y (specialist only)	*ICD-9 *codes 332, 332.0, or 332.1; or *ICD-10* codes G20, G21.0-G21.4, G21.8-G21.9, G22, or F02.3	PD and OP	MRR^g^	60.7 (53.8-67.7)	99.9 (99.8-99.9)	86.6 (80.8-92.3)	99.5 (99.4-99.6)
Butt et al,^21^ 2014^i^^,^^p^	Inclusions: 2 outpatient diagnosis ≥30 d apart in 2 y	*ICD-9 *codes 332, 332.0, or 332.1; or *ICD-10* codes G20, G21.0-G21.4, G21.8-G21.9, G22, or F02.3	PD and OP	MRR^g^	74.9 (68.7-81.0)	99.8 (99.7-99.8)	80.8 (75.0-86.6)	99.7 (99.6-99.8)
Butt et al,^21^ 2014^i^^,^^p^	Inclusions: 2 outpatient diagnosis ≥30 d apart in 3 y	*ICD-9 *codes 332, 332.0, or 332.1; or *ICD-10* codes G20, G21.0-G21.4, G21.8-G21.9, G22, or F02.3	PD and OP	MRR^g^	75.4 (69.3-81.5)	99.7 (99.7-99.8)	80.0 (74.2-85.8)	99.7 (99.6-99.8)
Butt et al,^21^ 2014^i^^,^^p^	Inclusions: 3 outpatient diagnosis ≥30 d apart in 1 y	*ICD-9 *codes 332, 332.0, or 332.1; or *ICD-10* codes G20, G21.0-G21.4, G21.8-G21.9, G22, or F02.3	PD and OP	MRR^g^	60.7 (53.8-67.7)	99.0 (99.0-100)	91.3 (86.4-96.2)	99.5 (99.4-99.6)
Butt et al,^21^ 2014^i^^,^^p^	Inclusions: 1 inpatient diagnosis or 2 diagnosis ≥30 d apart, all in 1 y	*ICD-9 *codes 332, 332.0, or 332.1; or *ICD-10* codes G20, G21.0-G21.4, G21.8-G21.9, G22, or F02.3	PD and OP	MRR^g^	74.9 (68.7-81.0)	99.8 (99.7-99.8)	80.3 (74.5-86.2)	99.7 (99.6-99.8)
Butt et al,^21^ 2014^i^^,^^p^	Inclusions: 1 outpatient diagnosis or 1 pharmaceutical claim	*ICD-9 *codes 332, 332.0, or 332.1; or *ICD-10* codes G20, G21.0-G21.4, G21.8-G21.9, G22, or F02.3	PD and OP	MRR^g^	89.5 (85.2-93.9)	97.6 (97.3-97.8)	33.0 (29.0-37.1)	99.9 (99.8-99.9)
Butt et al,^21^ 2014^i^^,^^p^	Inclusions: 1 outpatient diagnosis and 1 pharmaceutical claim	*ICD-9 *codes 332, 332.0, or 332.1; or *ICD-10* codes G20, G21.0-G21.4, G21.8-G21.9, G22, or F02.3	PD and OP	MRR^g^	77.0 (71.0-82.9)	99.8 (99.7-99.9)	84.5 (79.1-89.9)	99.7 (99.6-99.8)
Butt et al,^21^ 2014^i^^,^^p^	Inclusions: 1 pharmaceutical claim	*ICD-9 *codes 332, 332.0, or 332.1; or *ICD-10* codes G20, G21.0-G21.4, G21.8-G21.9, G22, or F02.3	PD and OP	MRR^g^	81.7 (76.2-87.2)	98.3 (98.1-98.5)	39.5 (34.7-44.3)	99.7 (99.7-99.8)
Butt et al,^21^ 2014^i^^,^^p^	Inclusions: 1 outpatient diagnosis and 1 pharmaceutical claim, all in 1 y	*ICD-9 *codes 332, 332.0, or 332.1; or *ICD-10* codes G20, G21.0-G21.4, G21.8-G21.9, G22, or F02.3	PD and OP	MRR^g^	75.9 (69.9-82.0)	99.9 (99.8-99.9)	87.3 (82.3-92.4)	99.7 (99.6-99.8)
Butt et al,^21^ 2014^i^^,^^p^	Inclusions: 2 outpatient diagnosis ≥30 d apart and 1 pharmaceutical claim, all in 1 y	*ICD-9 *codes 332, 332.0, or 332.1; or *ICD-10* codes G20, G21.0-G21.4, G21.8-G21.9, G22, or F02.3	PD and OP	MRR^g^	70.7 (64.2-77.1)	99.9 (99.8-99.9)	90.0 (85.2-94.8)	99.6 (99.5-99.7)
Butt et al,^21^ 2014^i^^,^^p^	Inclusions: (≥2 outpatient diagnosis ≥30 d apart in 1 y) or (1 pharmaceutical claim and 1 outpatient diagnosis)	*ICD-9 *codes 332, 332.0, or 332.1; or *ICD-10* codes G20, G21.0-G21.4, G21.8-G21.9, G22, or F02.3	PD and OP	MRR^g^	82.2 (76.8-87.6)	99.7 (99.6-99.8)	78.1 (72.4-83.8)	99.8 (99.7-99.8)
Butt et al,^21^ 2014^i^^,^^p^	Inclusions: (≥2 outpatient diagnosis ≥30 d apart in 1 y) or (1 pharmaceutical claim and 1 outpatient diagnosis 6 mo before or after pharmaceutical claim)	*ICD-9 *codes 332, 332.0, or 332.1; or *ICD-10* codes G20, G21.0-G21.4, G21.8-G21.9, G22, or F02.3	PD and OP	MRR^g^	81.7 (76.2-87.2)	99.7 (99.6-99.8)	80.0 (74.4-85.6)	99.7 (99.7-99.8)
Butt et al,^21^ 2014^i^^,^^p^	Inclusions: 1 pharmaceutical claim and 1 inpatient or outpatient diagnosis 6 mo before or after pharmaceutical claim	*ICD-9 *codes 332, 332.0, or 332.1; or *ICD-10* codes G20, G21.0-G21.4, G21.8-G21.9, G22, or F02.3	PD and OP	MRR^g^	76.4 (70.4-82.5)	99.9 (99.8-99.9)	87.4 (82.4-92.5)	99.7 (99.6-99.8)
Butt et al,^21^ 2014^i^^,^^p^	Inclusions: (1 inpatient diagnosis or ≥2 outpatient diagnosis ≥30 d apart in 1 y) or (1 pharmaceutical claim and 1 inpatient or outpatient diagnosis)	*ICD-9 *codes 332, 332.0, or 332.1; or *ICD-10* codes G20, G21.0-G21.4, G21.8-G21.9, G22, or F02.3	PD and OP	MRR^g^	82.2 (76.8-87.6)	99.7 (99.6-99.8)	76.2 (70.4-82.0)	99.8 (99.7-99.8)
Butt et al,^21^ 2014^i^^,^^p^	Inclusions: (1 inpatient diagnosis or 2 outpatient diagnosis ≥30 d apart in 1 y) or (1 pharmaceutical claim and 1 inpatient or outpatient diagnosis 6 mo before or after pharmaceutical claim)	*ICD-9 *codes 332, 332.0, or 332.1; or *ICD-10* codes G20, G21.0-G21.4, G21.8-G21.9, G22, or F02.3	PD and OP	MRR^g^	81.7 (76.2-87.2)	99.7 (99.6-99.8)	78.0 (72.3-83.7)	99.8 (99.7-99.8)
Data source type: EHR and linked claims								
Wei et al,^38^ 2016^i^	Inclusions: ≥1 pharmaceutical claim	NU	PD	MRR^g^	23.0 (NR)	NR (NR)	87.0 (NR)	NR (NR)
Wei et al,^38^ 2016^i^	Inclusions: ≥1 mention of PD in EHR	NU	PD	MRR^g^	93.0 (NR)	NR (NR)	33.0 (NR)	NR (NR)
Data source type: survey								
Jain et al,^26^ 2015^i^	Inclusions: ≥1 PD pharmaceutical claim (self-reported)	NU	PD	Framingham Heart Study^q^	91.3 (72.0-98.9)	100 (99.9-100)	100 (83.9-100)	99.9 (99.8-100)
Jain et al,^26^ 2015^i^	Inclusions: yes to ≥1 of the following (self-reported): diagnosed with PD, hospitalized with PD, or ≥1 PD pharmaceutical claim	NU	PD	Framingham Heart Study^q^	88.9 (73.9-96.9)	100 (99.9-100)	100 (89.1-100)	99.9 (99.7-100)
Jain et al,^26^ 2015^e^	Inclusions: yes to ≥1 of the following (self-reported): diagnosed with PD or hospitalized with PD	NU	PD	Framingham Heart Study^q^	92.9 (76.5-99.1)	100 (99.9-100)	100 (86.8-100)	99.9 (99.7-100)

Abbreviations: EHR, electronic health records; *ICD*, *International Classification of Diseases*; MCBS, Medicare Current Beneficiary Survey; MRR, medical record review; NPV, negative predictive value; NR, not reported; NU, not used; OP, other parkinsonisms; PD, Parkinson disease; PHY, physician or carrier claims; PPV, positive predictive value; SALT, Screening Across the Lifespan Twin study.

^a^ Table 2 provides more information on case definition groups.

^b^ Data source type refers to the type of data source to which the case definition was applied.

^c^ Information about each case definition’s other surveillance system attributes appears in eTable 10 in Supplement 1.

^d^ PD with OP refers to a reference standard that compares the case definition to a gold standard for PD and OP.

^e^ Case definition selected by authors of the cited study after they validated case definition(s) in their study.

^f^ PD cohort refers to a group of people previously diagnosed with PD.

^g^ MRR was referred to in the cited articles as “medical chart review.”

^h^ For the purposes of this study, administrative data–based studies were split into 2 categories: claims and other. Other administrative data source types included medical or billing information that is not claims based (eg, aggregated information pulled from the EHR for research or administrative purposes).

^i^ Case definition not selected by authors of the cited study after they validated case definition(s) in their study.

^j^ Group E case definitions did not include any exclusions.

^k^ Noyes et al^30^ reference standard using MCBS self-report of PD or self-reported use of PD medication.

^l^ Noyes et al^30^ reference standard using MCBS self-report of PD.

^m^ Feldman et al^23^ reference standard was SALT, a longitudinal study that included diagnoses of PD based on survey and specialist medical record review.

^n^ Butt et al^21^ case definition validated in a cohort of persons aged 20 years and older.

^o^ Butt et al^21^ selected 4 case definitions: (1) age 20 years and older with pharmaceutical claims, (2) age 20 years and older without pharmaceutical claims, (3) age 65 years and older with pharmaceutical claims, and (4) age 65 years and older without pharmaceutical claims.

^p^ Butt et al,^21^ 2014 case definition validated in a cohort of persons aged 65 years and older.

^q^ Jain et al,^26^ 2015 reference standard using Framingham Heart Study, a longitudinal study that included diagnosis of PD based on neurologist examination and MRR.

Validation data were available for 4 group A definitions.^27,28,35,36^ To maximize available accuracy data, one group A definition not chosen by the validation study authors was added.^36^ These 5 definitions were validated using a PD reference standard. While 2 definitions performed well on sensitivity, specificity, and positive predictive value (PPV), their performance on most other key attributes was limited. Group A definitions’ performance on other key attributes varied substantially. No definition maximized and balanced the key attributes.

Case definition validation data were available for 6 group E definitions, 2 of which were validated twice for different age groups.^20,21,30^ An additional 52 group E definitions not chosen by validation study authors were added to the analysis.^21,23,26,30,33,38^ Of these 60 definitions, most (53 [88%]) were validated using a PD and OP reference standard; among these, the specificity was uniformly high. The remainder (7 definitions [12%]) used a PD reference standard; among these, specificity was more variable. Across group E definitions, sensitivity, PPV, acceptability, and cost-effectiveness varied widely.

### Case Definitions Selection

Because no group A definition successfully maximized and balanced the key attributes, we utilized our review of parameter combinations to develop other potential group A definitions. We examined existing performance data to anticipate which newly developed definition would best maximize and balance performance across all key attributes; this was then selected as NNCSS’s PD probable case definition (eTable 11 in Supplement 1). The PD probable case definition required either (1) 2 or more inpatient or outpatient diagnostic claims for PD (ie, *ICD *code G20) at least 90 days apart or (2) 1 or more inpatient or outpatient diagnostic claims for PD and 2 or more pharmaceutical claims for PD medications at least 90 days after 1 diagnostic claim. Possible cases with 1 or more inpatient or outpatient diagnostic claims for OP (G21.X, G23.X, G31.83, G31.85, G90.3) at any time during the case ascertainment period were excluded.

Of all group E definitions, one best maximized and balanced the key attributes.^21^ It was selected as NNCSS’s PD possible case definition and adapted to utilize US *ICD* codes (eTable 11 in Supplement 1). The PD possible case definition required either (1) 2 or more outpatient diagnostic claims for PD or OP at least 30 days apart in a 1-year period or (2) 1 or more pharmaceutical claim for PD medications and 1 or more outpatient diagnostic claim for PD or OP within 6 months of each other in any order. Additional literature informed the lower age bound and drug codes utilized to identify US Food and Drug Administration (FDA)–approved PD medications in the NNCSS PD case definitions (eResults and eTable 12 in Supplement 1).

### Data Source Types: Evaluation and Selection

Performance on the key attributes varied for vital records, EHR, survey, and hospital discharge data sources (Table 4).^18,19,191,192,193,194,195,196,197^ In contrast, claims performed uniformly high across the key attributes due to characteristics including standardized coding systems, coverage of large discrete populations, high representativeness, and inclusion of demographic characteristics useful for weighting and subgroup analyses. At present, claims data have emerged as the most suitable source for supporting national PD surveillance efforts conducted by NNCSS.

**Table 4.** Evaluation of Data Source Types for NNCSS on Key Attributes of Public Health Surveillance Systems^a^

Key attribute^b^	Vital records^c^	EHR	Survey	Claims	Facility-based
Representativeness and coverage	High: all deaths	Low to moderate: data from one or an unspecified health system or system type	Moderate: often a convenience sample; costliness may limit sample size	High: all claims for all enrolled beneficiaries	Moderate: some are national, others are state based
Timeliness	Moderate: ≥1 y delay	Moderate to high: 1- to 6-mo delay depending on EHR	Low: 1- to 2-y delay depending on survey	Moderate to high: 1- to 6-mo delay; 2 y for some sources	Low: Approximately a 2-y delay
Stability^d^	High: no major concerns	High: no major concerns	High: no major concerns	High: no major concerns	High: no major concerns
Data quality	Low to moderate: overall high^e^	Moderate: incomplete data for outside health system(s); hard to interpret data (eg, problem list)	Low: recall bias is a particular concern for complex NCs^f^	Moderate to high: standardized coding can make NCs easy to identify	Moderate: data can vary from state to state but often standardized within a state
General attributes	High: multiple geographic and demographic characteristics; includes race and ethnicity	High: multiple geographic and demographic characteristics; may include race and ethnicity	Variable: varies by survey	High: multiple demographic and geographic characteristics; some sources include race and ethnicity	High: multiple demographic and geographic characteristics; typically includes race and ethnicity
Versatility	High: includes the complete population, identification of mutually exclusive groups not needed	Low: includes population of a specific health system, challenging to determine mutual exclusivity	Low: includes survey respondents, challenging to determine mutual exclusivity	High: includes discrete populations, often allows identification of mutually exclusive groups	Low: includes population of a specific health system or hospitalizations in a geographic area, challenging to determine mutual exclusivity
Ability to link to other sources	Moderate: likely able to link; may be cost-prohibitive	Moderate: likely able to link; varies by EHR	Low: likely able to link; varies by survey^g^	High: likely able to link	Low: unlikely able to link
Overall assessment of appropriateness^h^	Less appropriate	Less appropriate	Less appropriate	Most appropriate	Less appropriate

Abbreviations: EHR, electronic health records; NC, neurological condition; NNCSS, National Neurological Conditions Surveillance System; PD, Parkinson disease.

^a^ Data source types were assessed on the evaluation criteria relative to one another.

^b^ Key attributes of public health surveillance systems that are relevant to data source types, adapted from Groseclose and Buckeridge^18^ and El Burai Felix et al^19^; eTable 2 in Supplement 1 includes additional information.

^c^ For the purposes of NNCSS surveillance use for PD, vital records refer to death record data.

^d^ Stability refers to the reliability and availability of the data. There are no concerns for stability for these data sources, except for survey data, which may be less reliable due to their resource-intensive nature.

^e^ Cause of death attribution may not list PD on at least 50% of certificates.^191,192,193,194^

^f^ The overall data quality for surveys is high; however, for conditions such as PD, the accuracy of self-reported Parkinson disease status can be low.^195^

^g^ While some surveys may be able to link to other data sources (eg, National Health Interview Survey linkage to Medicare data),^196,197^ these types of linkages provide limited utility in producing national surveillance estimates for PD.

^h^ Final determination of appropriateness was based on assessment of data source types relative to one another in addition to verification that the selected data source type is appropriate for PD surveillance.

## Discussion

NNCSS’s PD surveillance methods were established through systematic review and evaluation of case definitions and data source types. Our systematic review identified substantial heterogeneity across 163 case definitions from 156 studies. This reflects the challenges of identifying PD in population-based data sources due to potential diagnostic uncertainty early in the disease course, overlapping *ICD* code definitions, and variable coding practices. To address these challenges, we selected 2 case definitions that maximize and balance performance on key attributes to use to produce an estimate range within which the true PD estimate likely exists. Claims data were determined to be the most appropriate source for NNCSS surveillance due to their representativeness, versatility, high-quality data, comprehensive demographic information, and broad coverage of the US population.

The PD probable case definition would provide the lower bound of the estimate range. The probable case definition prioritizes specificity by utilizing more stringent criteria to reduce the inadvertent inclusion of persons with OP. The PD possible case definition would provide the upper bound of the estimate range. The possible case definition prioritizes sensitivity by utilizing broader criteria, likely including some persons early in the PD diagnostic process who may have unclear or conflicting claims, of whom a portion will likely be diagnosed with some OP.

NNCSS’s approach to using multiple case definitions with varying parameter strictness is supported in the literature. French investigators utilized 4 definitions based on administrative data evidence of PD.^50^ Their highly probable definition prioritized specificity, and their probable definition prioritized sensitivity, similar to our probable and possible definitions. Researchers estimated PD incidence in the United Kingdom using definitions with varying stringency; their broadest definition was similar to our possible definition.^139^ In a German study,^137^ 2 definitions were selected to address limitations of claims data, particularly the risk of identifying false positives. Three additional international studies used similar methods.^20,22,81^ In the US and internationally, different levels of parameter strictness have been used for other conditions with possible diagnostic uncertainty or a complex diagnostic process, including asthma,^198,199^ coronary heart disease,^200^ diabetes,^201^ multiple sclerosis,^202^ and chronic obstructive pulmonary disease.^203^

While there are challenges in identifying PD cases in population-based data sources, our findings suggest using multiple case definition parameters to include cases and additional parameters to exclude noncases mitigated the issue. Our systematic evaluation identified PD case definitions that maximized and balanced the key attributes. The studies reviewed suggest the accuracy of our selected case definitions is high; validation of our case definitions is encouraged. Our PD case definitions were selected specifically to use to produce an estimate range for ongoing national surveillance. This approach may not align with other research goals, so researchers should carefully consider which characteristics best align with their goals and identify PD case definitions accordingly.

### Strengths and Limitations

Our systematic review and evaluation is one of the only studies to identify, compare, and evaluate PD case definitions. To our knowledge, it is the only assessment of case definitions and data source types for US national PD surveillance. We used rigorous, iterative methods at all stages, including a comprehensive search strategy, independent abstraction, and ongoing collaboration with public health surveillance experts, epidemiologists, and fellowship-trained movement disorders neurologists. Additionally, we included systematic evaluation approaches, such as developing methods to assess the risk of misclassification bias and using the widely accepted key attributes^18,19^ to assess case definitions and data source types.

Through a comprehensive, standardized assessment of all possible data source types accessible to CDC, we identified claims data as the best current option for NNCSS surveillance. Claims data have limitations and were not originally designed for surveillance. They rely on coding accuracy and completeness, and can be impacted by factors including access to care, particularly specialists, and demographic and geographic coverage that varies from the overall population. Strengths of claims data include high coverage, ability to combine data sources while ensuring unduplicated observations, and data attributes not found in other data source types.

This study also has limitations. Despite the systematic nature of our review, some relevant studies may have been missed. However, the impact of missed studies is likely low, as all heterogeneous case definitions we identified aligned with the case definition groups we defined. Furthermore, results may have been impacted by lower-quality studies that met inclusion criteria. While some quality assessment questions had low initial agreement, all had 100% agreement after reconciliation. For validation studies, the most frequently identified concern was for the patient selection domain, which may reflect study samples not representing the general population. Despite this, accuracy findings were relatively consistent across studies validating group A and E case definitions, so it is unlikely that validation study quality substantially influenced our case definition selections. Most nonvalidation studies had a minor RoB due to cases being identified in administrative data sources. This bias is expected for any clinical diagnosis proxy (the gold standard). The least biased proxy, expert medical record review, is not feasible at the population level. Despite this, nonvalidation study case definitions informed the development of case definition groups without any major outliers.

## Conclusions

In this systematic review and evaluation, we provide a foundation for NNCSS PD surveillance with the selection of claims data, which had high performance on key attributes, and 2 case definitions, which underscored the importance of distinguishing PD from OP. NNCSS will systematically appraise advancements in data source types (eg, EHR), clinical practice changes (eg, new FDA-approved treatments), and clinical research or diagnostic criteria advances (eg, biological staging, biomarkers)^204,205^ to ensure PD surveillance methods remain current. Increased understanding of PD burden through national surveillance can inform clinical and public health initiatives and drive research to better understand risk factors for developing PD, promote earlier diagnosis and intervention, and minimize patient and caregiver burden.

## Notes

Supplement 1. **eTable 1.** Systematic Review Search Strategy **eMethods 1.** Screening Inclusion and Exclusion Criteria **eMethods 2.** Quality Assessments **eTable 2.** Key Attributes of Public Health Surveillance Systems Used to Evaluate Case Definitions and Data Source Types **eFigure.** PRISMA Flowchart **eTable 3.** Study Characteristics **eTable 4.** Interrater Reliability of Quality Assessment of Validation Articles Using Modified QUADAS-2 Tool **eTable 5.** Risk of Bias and Applicability Concerns in Included Validation Studies Identified Using Modified QUADAS-2 Tool **eTable 6.** Interrater and Intrarater Reliability of Quality Assessment of Nonvalidation Articles Using Modified MORE Tool **eTable 7.** Poor Reporting and Bias Issues Identified in Included Nonvalidation Literature Using Modified MORE Tool **eTable 8.** NNCSS’s Assessment of Situational Factors to Determine Suitability of PD Case Definitions **eTable 9.** *ICD-9* and *ICD-10* Coding of Parkinson Disease and Other Parkinsonisms **eResults.** Additional NNCSS Case Definition Parameters **eTable 10.** Evaluation of Group A and Group E Case Definitions on Key Attributes of Public Health Surveillance Systems by Case Definition Group and Data Source Type—Part B: Other Surveillance System Attributes **eTable 11.** NNCSS’s Selected Parkinson Disease Surveillance Case Definitions and Characteristics **eTable 12.** Medications Used in NNCSS’s Parkinson Disease Case Definitions
eReferences.

Supplement 2.
Data Sharing Statement
