# Supplementary material for: Case Definitions and Data Sources for NNCSS Parkinson Disease Surveillance: A Systematic Review
Source: JAMA Netw Open. 2026 Jun 8;9(6):e2613928. doi: 10.1001/jamanetworkopen.2026.13928 (PMC13247814; doi:10.1001/jamanetworkopen.2026.13928)
Supplement: Supplement 1. — eTable 1. Systematic Review Search Strategy eMethods 1. Screening Inclusion and Exclusion Criteria eMethods 2. Quality Assessments eTable 2. Key Attributes of Public Health Surveillance Systems Used to Evaluate Case Definitions and Data Source Types eFigure. PRISMA Flowchart eTable 3. Study Characteristics eTable 4. Interrater Reliability of Quality Assessment of Validation Articles Using Modified QUADAS-2 Tool eTable 5. Risk of Bias and Applicability Concerns in Included Validation Studies Identified Using Modified QUADAS-2 Tool eTable 6. Interrater and Intrarater Reliability of Quality Assessment of Nonvalidation Articles Using Modified MORE Tool eTable 7. Poor Reporting and Bias Issues Identified in Included Nonvalidation Literature Using Modified MORE Tool eTable 8. NNCSS’s Assessment of Situational Factors to Determine Suitability of PD Case Definitions eTable 9. ICD-9 and ICD-10 Coding of Parkinson Disease and Other Parkinsonisms eResults. Additional NNCSS Case Definition Parameters eTable 10. Evaluation of Group A and Group E Case Definitions on Key Attributes of Public Health Surveillance Systems by Case Definition Group and Data Source Type—Part B: Other Surveillance System Attributes eTable 11. NNCSS’s Selected Parkinson Disease Surveillance Case Definitions and Characteristics eTable 12. Medications Used in NNCSS’s Parkinson Disease Case Definitions eReferences. [file jamanetwopen-e2613928-s001.pdf]

Supplemental Online Content

Esper CD, Hogan KN, Abeysekara P, Smith KN, Smith TG, Mercer SL. Case definitions and data sources for NNCSS Parkinson disease surveillance. *JAMA Netw Open.* 2026;9(5):e2613928. doi:10.1001/jamanetworkopen.2026.13928

eTable 1. Systematic Review Search Strategy

eMethods 1. Screening Inclusion and Exclusion Criteria

eMethods 2. Quality Assessments

eTable 2. Key Attributes of Public Health Surveillance Systems Used to Evaluate Case Definitions and Data Source Types

eFigure. PRISMA Flowchart

eTable 3. Study Characteristics

eTable 4. Interrater Reliability of Quality Assessment of Validation Articles Using Modified QUADAS-2 Tool

eTable 5. Risk of Bias and Applicability Concerns in Included Validation Studies Identified Using Modified QUADAS-2 Tool

eTable 6. Interrater and Intrarater Reliability of Quality Assessment of Nonvalidation Articles Using Modified MORE Tool

eTable 7. Poor Reporting and Bias Issues Identified in Included Nonvalidation Literature Using Modified MORE Tool

eTable 8. NNCSS's Assessment of Situational Factors to Determine Suitability of PD Case Definitions

eTable 9. *ICD-9* and *ICD-10* Coding of Parkinson Disease and Other Parkinsonisms

eResults. Additional NNCSS Case Definition Parameters

eTable 10. Evaluation of Group A and E Case Definitions on Key Attributes of Public Health Surveillance Systems by Case Definition Group and Data Source Type – Part B: Other Surveillance System Attributes

eTable 11. NNCSS's Selected Parkinson Disease Surveillance Case Definitions and Characteristics

eTable 12. Medications Used in NNCSS's Parkinson Disease Case Definitions

eReferences.

This supplemental material has been provided by the authors to give readers additional information about their work.

© 2026 Esper CD et al. *JAMA Network Open*.

eTable 1. Systematic Review Search Strategy[a]

Database	Strategy
Medline (OVID) 1946-	Parkinson Disease/ OR Parkinson*.ti,ab.<br>AND<br>Algorithms/ OR Case Algorithm* OR case identification* OR case certification OR case definition* OR case ascertainment OR case finding* OR case cohort* OR classification* OR category OR categories OR categorization OR patient characteristics OR clinical characteristic* OR ICD* OR G20 OR diagnostic code* OR diagnostic criteria OR coding OR data mining OR retrospective analysis OR retrospective stud* OR cohort stud* OR spatial analysis OR standardiz* OR population based<br>AND<br>Database* OR (claims ADJ5 data*) OR (analy* ADJ5 record*) OR data source* OR administrative data* OR health* data* OR survey* OR surveillance OR informatic* OR information system* OR health* system* OR insurance system* OR insurance data* OR health maintenance organization OR registry OR registries OR national probability sample* OR national data* OR electronic record* OR health record* OR medical record* OR vital records OR death records OR mortality records OR census data OR discharge data OR MarketScan OR CMS OR Medicare OR Medicaid OR PharMetrics OR (Healthcare Cost ADJ2 Utilization Project) OR pharmacy record* OR claims record* OR American Community Survey<br>NOT<br>exp animals/ not exp humans/<br><br>Limit English; 1980 -; Abstracts Available

© 2026 Esper CD et al. *JAMA Network Open*.

Database	Strategy
Embase (OVID) 1996-	Parkinson Disease/ OR Parkinson*.ti,ab.<br>AND<br>Algorithm/ OR Case Algorithm* OR case identification* OR case certification OR case definition* OR case ascertainment OR case finding* OR case cohort* OR classification* OR category OR categories OR categorization OR patient characteristics OR clinical characteristic* OR ICD* OR G20 OR diagnostic code* OR diagnostic criteria OR coding OR data mining OR retrospective analysis OR retrospective stud* OR cohort stud* OR spatial analysis OR standardiz* OR population based<br>AND<br>Database* OR (claims ADJ5 data*) OR (analy* ADJ5 record*) OR data source* OR administrative data* OR health* data* OR survey* OR surveillance OR informatic* OR information system* OR health* system* OR insurance system* OR insurance data* OR health maintenance organization OR registry OR registries OR national probability sample* OR national data* OR electronic record* OR health record* OR medical record* OR vital records OR death records OR mortality records OR census data OR discharge data OR MarketScan OR CMS OR Medicare OR Medicaid OR PharMetrics OR (Healthcare Cost ADJ2 Utilization Project) OR pharmacy record* OR claims record* OR American Community Survey cNOT<br>exp animal/ not exp human/<br>AND<br>Limit to remove Medline records<br><br>Limit English; 1980 -; Abstracts Available
PsycInfo (OVID) 1967-	Parkinson Disease's/ OR Parkinson*.ti,ab.<br>AND<br>Algorithms/ OR (Case Algorithm* OR case identification* OR case certification OR case definition* OR case ascertainment OR case finding* OR case cohort* OR classification* OR category OR categories OR categorization OR patient characteristics OR clinical characteristic* OR ICD* OR G20 OR diagnostic code* OR diagnostic criteria OR coding OR data mining OR retrospective analysis OR retrospective stud* OR cohort stud* OR spatial analysis OR standardiz* OR population based).ti,ab.<br>AND<br>Databases/ OR (claims ADJ5 data*) OR (analy* ADJ5 record*) OR data source* OR administrative data* OR health* data* OR survey* OR surveillance OR informatic* OR information system* OR health* system* OR insurance system* OR insurance data* OR health maintenance organization OR registry OR registries OR national probability sample* OR national data* OR electronic record* OR health record* OR medical record* OR vital records OR death records OR mortality records OR census data OR discharge data OR MarketScan OR CMS OR Medicare OR Medicaid OR PharMetrics OR (Healthcare Cost ADJ2 Utilization Project) OR pharmacy record* OR claims record* OR American Community Survey<br><br>Limit English; 1980 -; Abstracts Available

© 2026 Esper CD et al. *JAMA Network Open*.

Database	Strategy
CINAHL (EbscoHost)	(MH "Parkinson Disease") OR (TI,AB (Parkinson*))<br>AND<br>(MH "Algorithms") OR "Case Algorithm*" OR "case identification*" OR "case certification" OR "case definition*" OR "case ascertainment" OR "case finding*" OR classification* OR category OR categories OR categorization OR "patient characteristics" OR ICD* OR G20 OR "retrospective analysis" OR "spatial analysis" OR "case cohort*" OR "diagnostic code*" OR "diagnostic criteria" OR coding OR "data mining" OR "retrospective stud*" OR "cohort stud*" OR standardiz* OR "population based"<br>AND<br>Database* OR "claims data*" OR "data source*" OR "administrative data*" OR "health* data*" OR survey* OR surveillance OR informatic* OR "information system*" OR "health* system*" OR "insurance system*" OR "insurance data*" OR "health maintenance organization" OR registry OR registries OR "national probability sample*" OR "national data*" OR "electronic record*" OR "health record*" OR "medical record*" OR "vital records" OR "death records" OR "mortality records" OR "census data" OR "discharge data" OR MarkeaScan OR CMS OR Medicare OR Medicaid OR PharMetrics OR ("Healthcare Cost" N2 "Utilization Project") OR "pharmacy record*" OR "claims record*" OR "American Community Survey"<br>Limit English; 1980- ; exclude Medline records; Abstracts Available
Scopus	TITLE-ABS-KEY(Parkinson*) AND TITLE-ABS-KEY("Case Algorithm*" OR "case identification*" OR "case certification" OR "case definition*" OR "case ascertainment" OR "case finding*" OR classification* OR category OR categories OR categorization OR "patient characteristics" OR ICD* OR G20 OR "retrospective analysis" OR "spatial analysis" OR "case cohort*" OR "diagnostic code*" OR "diagnostic criteria" OR coding OR "data mining" OR "retrospective stud*" OR "cohort stud*" OR standardiz* OR population based) AND TITLE-ABS-KEY(Database* OR "claims data*" OR "data source*" OR "administrative data*" OR "health* data*" OR survey* OR surveillance OR informatic* OR "information system*" OR "health* system*" OR "insurance system*" OR "insurance data*" OR "health maintenance organization" OR registry OR registries OR "national probability sample*" OR "national data*" OR "electronic record*" OR "health record*" OR "medical record*" OR "vital records" OR "death records" OR "mortality records" OR "census data" OR "discharge data" OR MarketScan OR CMS OR Medicare OR Medicaid OR PharMetrics OR ("Healthcare Cost" N2 "Utilization Project") OR "pharmacy record*" OR "claims record*" OR "American Community Survey") AND NOT INDEX(medline)<br>Limit English; 1980 –; exclude Medline records; Abstracts Available

Abbreviations: CINAHL, Cumulative Index to Nursing and Allied Health; CMS, Centers for Medicare and Medicaid Services; ICD, International Classification of Diseases.

[a] The literature search was conducted to inform selection of case definitions and development of estimates. The search was then updated to ensure the selected case definitions reflected all available literature.

© 2026 Esper CD et al. *JAMA Network Open*.

eMethods 1. Screening Inclusion and Exclusion Criteria

Inclusion

***Data source type**:* Administrative, including claims data, hospital discharge data, or similar; electronic health records or electronic medical records; vital records/death records; population-based survey data; aggregated, linked, de-identified data/ sources; or other sources consistent with data source types available within or accessible to CDC

Scope of data: 1) US or international and 2) national data, a national probability sample, or regional or local data if population is ≥500 000 (assumed adequate size for population-based estimates). Exception: <500 000 permissible for studies that validate Parkinson disease (PD) case definitions

Case Definition: 1) Was used to produce PD prevalence or other population-based estimates, or created or validated as part of the study; and 2) used one or more of *ICD-9* or *-10* codes, medication codes, other coding system-based definitions, or other types of parameters utilized in population-based data sources

Exclusion

Data source type: Medical records/charts, non-population-based surveys (eg, door-to-door)

Scope of data: Data from any locality that does not meet the scope of data inclusion criteria

Case Definition: Used chart review, machine learning, or direct application of diagnostic criteria/use of physician diagnosis

© 2026 Esper CD et al. *JAMA Network Open*.

eMethods 2. Quality Assessments

Quality Assessment for Validation Studies

The Quality Assessment of Diagnostic Accuracy Studies (QUADAS-2)[1] tool was selected to assess quality of validation studies because it assesses bias of studies in systematic reviews of diagnostic accuracy studies and is applicable to the varied data source types used by studies in our review. While broadly applicable, the authors of QUADAS-2 focused on systematic reviews of index tests to be used in clinical settings. Consistent with author recommendations, we modified the QUADAS-2 tool to optimize it for use with our research question and goals, which address the use of surveillance case definitions (ie, index tests) in population-based data sources.

Reviewers (K.N.H., K.N.S., T.G.S.) studied and discussed the modified QUADAS-2 coding manual. Reviewers completed training studies (n = 5) and coding discrepancies were resolved by discussion to reach consensus. Next, each of two reviewers (K.N.S., T.G.S.) applied QUADAS-2 to all remaining validation studies (n = 18) to enable calculation of interrater reliability (eTable 4). Intrarater reliability was not calculated due to the low number of studies. Discrepancies were resolved by discussion to reach consensus (K.N.S., T.G.S.), with remaining disagreements resolved by a third reviewer (K.N.H.). Consistent with author recommendations, we report the domain bias and domain applicability questions but not the signaling questions. QUADAS-2 results are reported by study in eTable 5.

Quality Assessment for Studies without Validation

The Methodological Evaluation of Observational Research (MORE)[2] tool was selected to assess quality of nonvalidation studies as it is designed for studies of chronic disease prevalence and is applicable to the varied case identification data source types included in our review (eg, survey, claims, etc.). In accord with author recommendations, we modified the MORE tool, optimizing it for use with our research question and goals which address studies describing prevalence or other public health surveillance measures (eg, incidence, healthcare use) of PD.

Reviewers (K.N.H., K.N.S., T.G.S.) studied and discussed the modified MORE coding manual. Reviewers completed training studies (n = 5) and discrepancies were resolved by discussion to reach consensus. Reviewers (K.N.S., T.G.S.) then applied modified MORE to the remaining nonvalidation studies (n = 133), of which 40% were coded by both reviewers independently to enable calculation of interrater reliability (eTable 6). In addition, reviewers re-coded studies to enable calculation of intrarater reliability (eTable 6). Discrepancies were resolved by discussion to reach consensus (K.N.S., T.G.S.), with remaining disagreements resolved by a third reviewer (K.N.H.). MORE results are reported in eTable 7.

© 2026 Esper CD et al. *JAMA Network Open*.

eTable 2. Key Attributes of Public Health Surveillance Systems[a] Used to Evaluate Case Definitions and Data Source Types

Case definitions	Population-based data source types
Accuracy	Representativeness/coverage
Sensitivity	Timeliness
Specificity	Stability
Positive predictive value	Data quality
Negative predictive value	General attributes of data
Simplicity	Versatility of data
Acceptability	Ability to link to other sources
Cost effectiveness	
Reproducibility	
Scalability/spreadability	

[a] Key attributes used in the evaluation of the performance of data source types and case definitions, adapted from Groseclose & Buckeridge,[3] with the exception of general attributes of data and versatility of data, which were adapted from El Burai Felix et al.[4]

eFigure 1. PRISMA Flowchart

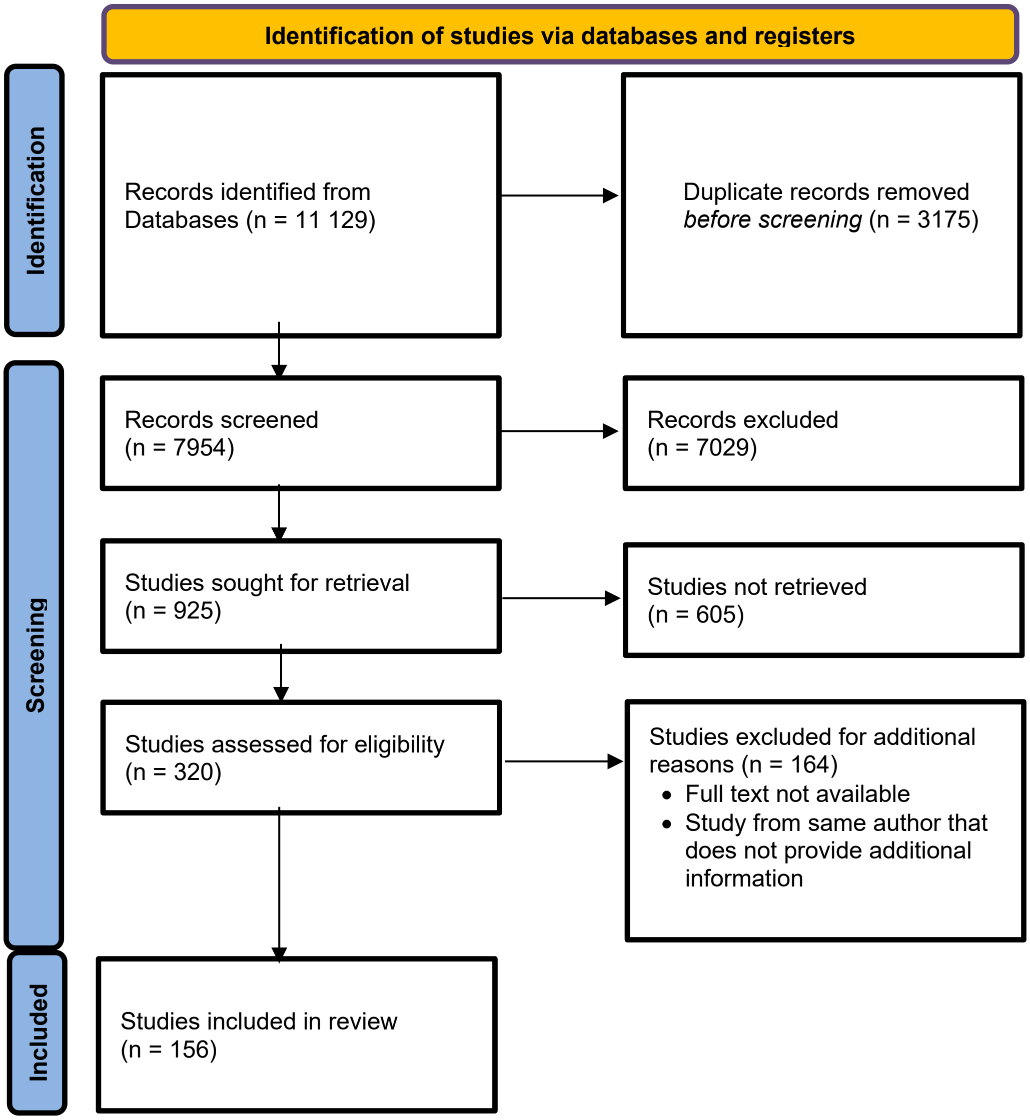

Reference: Page MJ, et al. BMJ 2021;372:n71. doi: 10.1136/bmj.n71.

© 2026 Esper CD et al. *JAMA Network Open*.

eTable 3. Study Characteristics

Source	Country	Data source type(s)[a,b]	Data source(s)	ICD (or other) code(s)	Case definition selected or used	Exclusion criteria	Case definition group[c]
Aamodt et al, 2021[5]	US	Facility-reported	HCUP	332.0	1 Inpatient Dx	NU	C
Aamodt et al, 2023[6]	US	Claims	Medicare	332, G20	≥2 Inpatient or outpatient Dx	1 Dx: 332.1, 333.0, 094.82,G21, G21.0, G21.2, G21.3, G21.4, G21.8, G21.9	B
Abuhasira et al, 2019[7]	Israel	Claims	CHS	NU	Rx with high purchase frequency (9 of 12 mo) for over 24 mo	Age <20 y or ≥85 y at time of first Rx purchase	Drug tracer
Albarmawi et al, 2022[8]	US	Claims	Medicare	332.0	≥2 Inpatient or outpatient Dx, 6-12 mo apart	Age <65 y	C
Alonso et al, 2009[9]	UK	OA	GPRD	NR[d]	≥1 Outpatient Dx + ≥2 Rx	NU	D
Baldacci et al, 2015[10]	Italy	Claims; OA	Tuscany longitudinal records linkage system for chronic diseases	*Selected case definitions 1 and 2:* 332	*Selected case definition 1:* ≥1 of:<br>1. ≥1 Inpatient Dx<br>2. PD exemption<br>3. ≥2 Rx ≥6 mo apart in 1 y (limited list of Rx)<br>*Selected case definition 2:* ≥1 of:<br>1. ≥1 Inpatient Dx<br>2. PD exemption<br>3. ≥2 Rx ≥6 mo apart in 1 y (longer list of Rx)	NU	*Selected case definition 1:* E<br><br>*Selected case definition 2:* D
Barer et al, 2022[11]	Israel	EHR	Maccabi Health Service	332.0	≥1 Inpatient or outpatient Dx (by neurologist) + ≥1 Rx	NU	C
Becker et al, 2008[12]	UK	OA	GPRD	*OXMIS codes*: 342, 342 D<br>*Read codes:* F12.0, F12z.00, F120.00	≥1 Outpatient Dx + ≥2 Rx post index Dx	1. Age <40 y<br>2. Rx: typical antipsychotics, metoclopramide, cinarizine	C
Becker et al, 2011[13]	UK	OA	GPRD	*OXMIS codes:* 342, 342 D	≥1 Outpatient Dx + ≥2 Rx	1. Age <40 y<br>2. Within 180 days of PD Dx Rx of typical antipsychotic	B

© 2026 Esper CD et al. *JAMA Network Open*.

Source	Country	Data source type(s)[a,b]	Data source(s)	ICD (or other) code(s)	Case definition selected or used	Exclusion criteria	Case definition group[c]
				Read codes: F12.0, F12z.00, F120.00		drugs, metoclopramide, cinnarizine	
Bhattacharjee et al, 2015[14]	US	Claims	Humana MA Part D	332.XX	≥1 Inpatient Dx <u>OR</u> ≥2 outpatient Dx	Age <65 y	D
Bhattacharjee et al, 2018[15]	US	Survey	NAMCS; NHAMCS	332.XX	≥1 Outpatient Dx	Age <65 y	D
Blin et al, 2015[16]	France	Claims; OA	EGB	*Selected case definitions 1 and 2:* G20	*Selected case definition 1:* ≥1 Inpatient Dx <u>OR</u> ≥1 PD Dx in long-term disability database *Selected case definition 2:* ≥1 inpatient Dx <u>OR</u> ≥1 PD Dx in long-term disability database <u>OR</u> (≥3 Rx in 1 y + ≥3 Rx in the following 1 y)	*Selected case definitions 1 and 2:* Age <18 y *Selected case definition 2:* 1. 1-2 neuroleptic Rx 6 mo prior to and 1 y after index Dx 2. 1-2 metaclopramide or metopimazine Rx 6 mo prior to index Dx 3. 1-2 cinnarizin or flunaraizine Rx in 1 y prior to and 1 y after index Dx 4. In 1 y only Rx were those used for RLS, hyperprolactemia, or MS 5. ≥3 Rx: any neuroleptic, metoclopramide, metopimizine, cinnarizine, flunarizine	*Selected case definition 1:* C *Selected case definition 2:* B
Brakedal et al, 2022[17]	Norway	OA	NorPD	ICD-10, ICPC-2, disease or disease-group reimbursement codes	(PD Rx (alone) <u>OR</u> PD Rx + Rx for PD (identified by Dx, primary care Dx, or disease or disease-group reimbursement code)) ≥3 consecutive times ≥30 d apart <u>AND</u> first and last Rx ≥180 d apart	1. Age of onset >80 y 2. Age of Dx <30 or >100 y Dopaminergic drugs for non-PD indications with duration <1 y 3. Off-treatment period[e] >2 y	Drug tracer
Brandt-Christens	Denmar k	OA	The Danish Medicinal	NU	≥1 Rx	1. Age <30 y	Drug Tracer

© 2026 Esper CD et al. *JAMA Network Open*.

Source	Country	Data source type(s)[a,b]	Data source(s)	ICD (or other) code(s)	Case definition selected or used	Exclusion criteria	Case definition group[c]
en et al, 2006[18]			Product Statistics			2. Women aged 30-40 y if only APD purchased was bromocriptine or cabergoline	
Bronskill et al, 2022[19]	Canada	Claims	OHIP	NR	≥3 Outpatient Dx ≥30 d apart (all within 2 y)	Age <40 y	B
Bůřil et al, 2021[20]	Czech Republic	Claims	NHIS; NRRHS	G20, G23.1, G23.2, G31.8	≥1 Dx (any setting)	NU	E
Butt et al, 2014[21]	Canada	Claims; OA	CIHI DAD; OHIP; NACRS; SDS	*Selected case definitions 1 and 2:* 332,0, 332.1, 332, G20, G21.0.04, G21.8-.9, G22, F02.3	*Selected case definition 1:* ≥2 outpatient Dx ≥30 d apart, within 1 y *Selected case definition 2:* ≥1 Rx + ≥1 inpatient Dx before or after Rx	*Selected case definition 1:* Age <20 y *Selected case definition 2:* Age <65 y	*Selected case definitions 1 and 2:* E
Callaghan et al, 2012[22]	US	OA; vital records	CA PDD; CA VSD	332.0, G20	1 Inpatient Dx OR death certificate underlying case of death	NU	C
Camacho-Soto et al, 2018[23]	US	Claims	Medicare	332, 332.0	≥1 Inpatient or outpatient Dx	1. Enrolled in Medicare for ≥2 y, making cases and controls aged ≥66 y 11 mo<br>2. Dx: secondary or atypical pkism[f]	B
Carriere et al, 2017[24]	France	Claims; OA	PMSI; SNIRAM	G20, F023 (and subcodes)	Combination of ≥3:<br>1. ≥1 Inpatient Dx<br>2. Designated "long-duration disease" beneficiaries with PD<br>3. ≥3 Rx	1. Dx: G21 (and subcodes), G318 (and subcodes), G23 (and subcodes), G903 (and subcodes)<br>2. Specific doses of apomorphine Chabre, rotigotine, pramipexole, lisuride, bromocriptine used for other indications<br>3. Central anticholinergic Rx<br>4. No Rx use in study period (1 y)	A

© 2026 Esper CD et al. *JAMA Network Open*.

Source	Country	Data source type(s)[a,b]	Data source(s)	ICD (or other) code(s)	Case definition selected or used	Exclusion criteria	Case definition group[c]
Cepeda et al, 2019[25]	US	Claims	MarketScan (commercial, Medicare, Medicaid); Optum (commercial, Medicare)	332.0, 332.1, G20, G21.0-.04, G21.8-.9, G22, F02.3	≥2 Inpatient or outpatient Dx (in 12 mo)	Age <65 y	E
Chandler et al, 2021[26]	US	Claims	Humana (MAPD, commercial)	332.X, G32.X	>2 Dx (any setting) OR >1 Dx (any setting) + >1 Rx	NU	D
Chang et al, 2016[27]	Taiwan	Claims	NHIRD	332	≥1 Inpatient or outpatient Dx (neurologist) + ≥1 Rx + ≥3 outpatient Dx (consecutive)	1. Age <40 y 2. Dx prior to incident Dx: dementia, psychosis, stroke, pneumonia and septicemia or acute respiratory failure with pneumonia	A
Chekani et al, 2016[28]	US	Survey	MEPS	332.XX	≥1 Dx	1. Age <19 y 2. Dx of both PD and dementia	D
Chekani et al, 2020[29]	US	Claims	Medicare	332.0	≥1 Inpatient or outpatient Dx	Age <65 y	C
Chen et al, 2012[30]	Taiwan	Claims	NHIP – LHID	332.0	≥3 Outpatient Dx + Rx for ≥60 d	1. Age <50 y or >80 y 2. Dx in index y: 430-434, 334, 331.3-331.4	B
Chen et al, 2015[31]	Taiwan	Claims	LHID	332.0	≥2 Outpatient Dx OR ≥1 inpatient Dx	NU	C
Chen CY et al, 2017[32]	Taiwan	Claims	NHI	332	≥3 Outpatient Dx in 1 y OR ≥1 inpatient Dx	1. Age <40 y 2. Dx prior to index: dementia, diabetes, stroke	D
Chen H et al, 2017[33]	Canada	Claims; OA	Canadian Institute for Health Information hospital	332	≥2 Outpatient Dx within 1 y OR ≥1 outpatient Dx + 1 Rx within 6 mo	Age <55 y or >85 y	D

© 2026 Esper CD et al. *JAMA Network Open*.

Source	Country	Data source type(s)[a,b]	Data source(s)	ICD (or other) code(s)	Case definition selected or used	Exclusion criteria	Case definition group[c]
			discharges; OHIP; ODB				
Chen et al, 2018[34]	Taiwan	Claims	NHIRD	332.0	≥1 Inpatient or outpatient Dx	Age <40 y	C
Chen SF et al, 2020[35]	Taiwan	Claims	NHIP - LHID	332	≥1 Outpatient Dx (neurologist) + ≥1 Rx	Rx of neuroleptics or metoclopramide ≤180 d prior to incident PD Dx	C
Chen W et al, 2020[36]	Canada	Claims; OA	BC provincial health administrative databases	332.XX, G20	≥1 Inpatient or outpatient Dx + ≥1 Rx within 90 d	1. Age <40 y<br>2. Dx of PD and Dx: 332.1X, 333.0X, 333.1X, G21.XX, G23.9X, R25.XX	B
Chen et al, 2021[37]	Taiwan	Claims	NHIRD	332.0	≥2 Inpatient or outpatient Dx + ≥ 1 Rx	1. Age <45 y<br>2. History of stroke<br>3. Antipsychotic Rx prior to index PD Dx	B
Chillag-Talmor et al, 2011[38]	Israel	Claims	Maccabi Health Services	NU	Applied to 3 consecutive y with highest Rx purchase frequency: 9 of 12 mo Rx (levodopa, dopamine agonists, or MAOB-I) <u>OR</u> 18 of 24 mo Rx (amantadine or MAOB-I) <u>OR</u> 6 of 12 mo simultaneous purchase of combination PD Rx	1. Age <20 y or >84 y<br>2. Rx of anticholinergic agents<br>3. Women age <50 y at first PD Rx purchase	Drug tracer
Choi et al, 2019[39]	South Korea	Claims	HIRA	G20	≥2 Inpatient or outpatient Dx	NU	C
Chou et al, 2017[40]	Taiwan	Claims	NHIRD	332	≥1 Inpatient or outpatient Dx	1. Age <40 y<br>2. Dx to avoid misdiagnosis as sec pkism prior to Dx or during study period: 986, 291, 292, 293.0, 293.1, 293.82, 295-299, 290.11, 290.12, 290.20, 290.3, 290.41, 290.42, 290.8, 290.9, 294.11	B

© 2026 Esper CD et al. *JAMA Network Open*.

Source	Country	Data source type(s)[a,b]	Data source(s)	ICD (or other) code(s)	Case definition selected or used	Exclusion criteria	Case definition group[c]
Connolly et al, 2015[41]	US	Claims	Medicare	332.0	≥1 Inpatient or outpatient Dx + ≥2 Rx	1. Age <65 y<br>2. Dx of sec pkism	B
Cortese et al, 2018[42]	Norway	Claims; OA	National Registry; NorPD; NUDB	NR[d]	1 Rx of levodopa for 365 DDD with associated inpatient or outpatient Dx of PD	NU	B
Crispo et al, 2015[43]	US	EHR	Cerner Health Facts	332, 332.0	≥1 Inpatient or outpatient Dx + ≥1 Rx	1. Age <40 y<br>2. Dx (primary or secondary) at any time: 332.1, 333.0	B
Crispo et al, 2016[44]	US	EHR	Cerner Health Facts	332, 332.0	≥2 Inpatient Dx >2 d apart + ≥1 Rx	1. Age <40 y<br>2. Dx (primary or secondary): 332.1, 333.0	B
Crispo et al, 2020[45]	Canada	Claims; OA	OHIP; NACRS	332.0, G20 *OHIP code:* 332	≥2 Outpatient Dx ≥30 d apart (all within 1 y)	1. Age <40 y<br>2. 5 y prior to or within 1 y after PD Dx: ≥1 Dx of PD, sec pkism, atypical pkism, DBS surgery	A
Dahodwala et al, 2009[46]	US	Claims	Medicaid	332.0	≥1 Inpatient or outpatient Dx	1. Age <40 y or >65 y<br>2. Dx of 332.1 in 12 mo prior to Dx in study period<br>3. ≥1 Dx of common causes of sec pkism: 433-436, 295, 296.4-296.9	B
Dahodwala et al, 2016[47]	US	Claims	Medicare	332.0	≥1 Inpatient Dx <u>OR</u> ≥2 outpatient Dx in 1 y	1. Age <65 y<br>2. History of: 295, 332.1, 333.X	B
Dahodwala et al, 2020[48]	US	Claims	Medicare	332.0	≥1 Inpatient or outpatient Dx + ≥ 1 Rx (levodopa)	Age <65 y	C
Dammertz et al, 2023[49]	Germany	Claims	SHI	G20	≥2 Outpatient Dx (with “confirmed Dx” identification) in ≥2 of 4 quarters + ≥1 PD Rx in ≥the first quarter of PD Dx	Age <50 y	B
Danila et al, 2014[g,50]	Canada	Claims; OA	HCRS; CCRS; OMHRS; DAD; NACRS	G20, G21, G22, F02.3	Meeting any of the following criteria:<br>1. 1 Dx in index assessment[g] or ≥1 Dx (LTC, HBCCF,	NU	D

© 2026 Esper CD et al. *JAMA Network Open*.

Source	Country	Data source type(s)[a,b]	Data source(s)	ICD (or other) code(s)	Case definition selected or used	Exclusion criteria	Case definition group[c]
					HC, inpatient, or ED) in entire history<br>2. 1 Dx in index assessment or ≥1 Dx (LTC, HBCCF, HC, inpatient, ED) in 2 y look-back<br>3. 1 Dx in index assessment or ≥1 Dx (inpatient, ED) in 2 y look-back<br>4. 1 Dx in index assessment		
De Vera et al, 2008[51]	Canada	Claims; OA	BCLHD	332	≥1 Inpatient or outpatient Dx + ≥2 Rx	Age <65 y	D
DeMarco et al, 2023[52]	US	EHR	Optum	332.0, G20	≥1 Inpatient Dx OR ≥2 outpatient Dx within 12 mo	NU	C
Doblhammer et al, 2018[53]	Germany	Claims	AOK	G20, G21, G22	≥1 Outpatient Dx (flagged as verified) OR ≥ 1 inpatient Dx (discharge or secondary)	Age <95 y	D
Dore et al, 2009[54]	US	Claims	Medicaid	332.0, 333.0, 781.0	(≥1 Inpatient or outpatient Dx OR ≥1 Rx) + ≥1 Inpatient Dx	1. Age <40 y<br>2. Dx: bone cancer, bone infections, sec pkism, schizophrenia, schizoaffective disorder<br>3. Rx: conventional antipsychotic	D
Etminan et al, 2008[55]	Canada	Claims, OA	BCLHD	332	≥1 Outpatient Dx + ≥2 Dx within 6 mo of PD Dx	NU	D
Eusebi et al, 2019[56]	Italy	Claims; OA	Umbra regional health information system databases	332.XX *Exemption code:* 038	≥1 Inpatient Dx OR exemption for medical charges OR ≥3 Rx in 6 mo	1. Before PD Rx, Rx: antipsychotic drugs<br>2. Rx consistent with atypical pkisms	B
Fan et al, 2019[57]	US	Claims	Medicare	332, 332.0	≥1 Inpatient or outpatient Dx	1. Age <67 y or >90 y<br>2. Dx in any y: 331.82, 333.0	D
Fang et al, 2020[58]	Taiwan	Claims	NHIRD	332.0	≥3 Outpatient Dx (from consecutive visits) OR ≥1 inpatient Dx	1. Dementia Dx prior to PD Dx	A

© 2026 Esper CD et al. *JAMA Network Open*.

Source	Country	Data source type(s)[a,b]	Data source(s)	ICD (or other) code(s)	Case definition selected or used	Exclusion criteria	Case definition group[c]
						2. ≥3 Dopaminergic Rx within 3 mo prior to PD Dx<br>3. No PD Rx after PD Dx	
Faust et al, 2020[59]	US	Claims	Medicare	332, 332.0	≥1 Inpatient or outpatient Dx	1. Age <65 y<br>2. Dx: 332.1, 333, 333.0, (322 or 332.0 without CPT 62270)	D
Feldman et al, 2011[60]	Sweden	OA	NPR; CDR	332.0, G20	≥1 Inpatient Dx (in NPR) <u>OR</u> PD listed as cause of death (in CDR)	1. Any Dx of pkism (in NPR): 332.1, 333.0, G21, G23.1, G23.2, G23.9, G25.9<br>2. Pkism as cause of death (in CDR): 332.1, 333.0, G21, G23.1, G23.2, G23.9, G25.9	B
Feldman et al, 2012[61]	Sweden	OA	NPR	332.0, G20	≥1 Inpatient Dx (primary)	1. Age <50 y<br>2. Inpatient Dx (secondary or tertiary) of PD<br>3. "PD-like" Dx: 333.0, G21.4, G21.8, G21.9, G23.1, G23.2, G23.9, G25.9	B
Finkelstein et al, 2007[62]	Canada	Claims	OHIP; ODB	332	≥1 Rx <u>OR</u> ≥1 inpatient or outpatient Dx	Dx of 333	D
François et al, 2017[63]	US	Claims	MarketScan (commercial); Medicare	332.0	(≥1 Inpatient or outpatient Dx <u>OR</u> ≥1 Rx before index Dx) + (≥1 inpatient or outpatient Dx <u>OR</u> ≥1 Rx after index Dx)	1. Age <18 y (MarketScan); <65 y (Medicare)<br>2. During study period Dx: autonomic failure/ neuropathy, MSA	C
Freedman et al 2016[64]	US	Claims	Medicare	332.0	≥1 Inpatient Dx <u>OR</u> ≥2 outpatient Dx ≥ 30 d apart	Age <66 y or >83 y	C
Fullard et al, 2018[65]	US	Claims	Medicare	332, 332.0	≥1 Inpatient or outpatient Dx	1. Age <65 y<br>2. Dx: 332.1, 333.0	B
Gandhi et al, 2021[66]	US	Claims	Medicare	332.0	≥2 Inpatient or outpatient Dx 6-12 mo apart	NU	C

© 2026 Esper CD et al. *JAMA Network Open*.

Source	Country	Data source type(s)[a,b]	Data source(s)	ICD (or other) code(s)	Case definition selected or used	Exclusion criteria	Case definition group[c]
Gordon et al, 2012[67]	US	OA	IHS	*Selected case definitions 1-3:* 332.0	*Selected case definition 1:* medical record listing PD code + (≥1 inpatient Dx OR ≥2 outpatient Dx)<br>*Selected case definition 2:* ≥1 inpatient Dx OR1 inpatient Dx OR ≥2 outpatient Dx<br>*Selected case definition 3:* (≥1 inpatient Dx OR ≥2 outpatient Dx during initial 5 study period y) + ≥1 Dx in final 4 study period y	*Selected case definitions 1-3:* NU	*Selected case definitions 1-3:* C
Gordon et al, 2013[68]	US	OA	IHS	332.0	1 Inpatient Dx OR 1 outpatient Dx (primary or secondary Dx)	NU	C
Gordon et al, 2015[69]	US	OA	IHS	332.0	≥2 Inpatient or outpatient Dx	NU	C
Guo et al, 2014[70]	Taiwan	Claims	NHIRD	332.X	≥3 Outpatient Dx OR ≥1 inpatient Dx	1. Rx with high risk of DIP for ≥3 mo before PD Dx<br>2. Dx: 430-438/A290-A294, A299, 290, 331.0, 331.2/A210, 00321, 0065, 0130-33, 0136, 0360-61, 0460-63, 047, 0490, 0491, 0520, 0530, 0543, 05472, 0550, 05601, 062-64, 0721-22, 09041-42, 0941-42, 09481-82, 09487, 09882, 10081, 11283, 1142, 11501, 11511, 11591, 1300, 1390, 320-23, 3240, 3249, 325-26, 800-804, 850-854, 742.3, 741.0, 331.3-.4, 191, 192.0-.1, 192.8-.9, 194.3-.4, 198.3, 237.0-.1, 237.5-.6, 237.9, 239.6-.7, 2250, 2252, 2273-74, 22802, 2750-51, 3334, 334, 740, 348.1, 997.01, 639.8,<br>3. 669.4, 768.7, 779.2<br>4. 3. Never received PD Rx	A

© 2026 Esper CD et al. *JAMA Network Open*.

Source	Country	Data source type(s)[a,b]	Data source(s)	ICD (or other) code(s)	Case definition selected or used	Exclusion criteria	Case definition group[c]
Guttman et al, 2003[71]	Canada	Claims	OHIP; ODB; RPDB	332	≥1 Outpatient Dx (from general/family physician or internal medicine) OR ≥1 Rx OR 1 outpatient Dx + 1 Rx (All criteria above must be met in both 2 y periods within 4 y study period)	1. Age <25 y 2. Cases meeting qualifications in only one of 2 y periods within 4 y study period	D
Han et al, 2019[72]	South Korea	Claims	HIRA Service database	G20	≥1 Inpatient or outpatient Dx (primary or any sub-diagnosis) + ≥60 d supply Rx (prescribed by neurologist)	NU	C
Heinzel et al, 2018[73]	Germany	Claims	InGef	G20	≥1 HD Dx (primary or secondary Dx only) OR ≥2 Amb Dx (verified Dx) in different quarters OR ≥2 Amb Dx (verified Dx) by different physicians OR ≥1 Amb Dx (verified Dx) + ≥1 Rx (All criteria above must be met in 1 calendar y)	NU	C
Hernán et al, 2006[74]	UK	OA	GPRD	NR[d]	≥1 Outpatient Dx + ≥2 Rx	NU	C
Hill et al, 2023[75]	US	EHR	University of Cincinnati EHR	332.0, G20	≥2 Inpatient or outpatient Dx (any diagnostic position) + ≥1 Rx	Dx: 331.82, 333.0, 331.6, 331.1, 334.3, 333.4, 334.9, 90.3, G 31.83, G23.1, G23.9, G31.95, G31.XX, G31.2, G21.XX, G91.2, G25.XX, R25.1, R27.0, G32.81, G11.XX, F25, F20-33.XX, F44.XX, F45.XX, F68.XX, T42.50A, T42.8X1A, G24, A52.19, F10.15, G10, G130.XX, G32.81, I69.893	B
Holt et al, 2010[h,76]	US	Claims	NR[g]	332.0	≥2 Inpatient or outpatient Dx ≥30 d apart	1. At any time during study period, Dx: 332.1, 331.82,	B

© 2026 Esper CD et al. *JAMA Network Open*.

Source	Country	Data source type(s)[a,b]	Data source(s)	ICD (or other) code(s)	Case definition selected or used	Exclusion criteria	Case definition group[c]
						295.XX, 293.83, 296.0X-296.90, 290.11, 290.3, 290.41, 291.0, 292.81, 293.0-293.1<br>2. During pre-index period, Dx: 291.5, 292.11, 291.X	
Horsfall et al, 2013[77]	UK	OA	THIN	*Read codes:* F12..00, F12z.00, F130300, F11x900	1 Read code: PD, PD NOS, Parkinsonism with orthostatic hypotension, cerebral degeneration in PD	*Read code:* Eu02300	B
Huse et al, 2005[78]	US	Claims	MarketScan (commercial, Medicaid)	332.0	≥2 Inpatient or outpatient Dx OR<br>≥1 inpatient or outpatient Dx + ≥2 Rx	NU	C
Iketani et al, 2020[79]	Japan	Claims	MIA databank	G20	≥1 Inpatient Dx + ≥1 PD Rx + hospitalized ≥3 days	1. Age <50 y<br>2. Dx: schizophrenia, bipolar disorder, DLB	B
Jain et al, 2015[80]	US	Survey	FHS; Medicare	NU	Self-report: PD Dx, PD hospitalization, or PD Rx	1. Age <65 y<br>2. Self-report of drugs that cause DIP	B
Jeong et al, 2019[81]	South Korea	Claims	NHI elderly cohort database; NHSP	G20	≥1 Inpatient or outpatient Dx + ≥1 Rx	1. Age <60 y<br>2. In 4 y prior to index PD Dx, Dx: G21-G23, G25, G26, I60-I69, F01-F99, G30-G31<br>3. Dx: G21-G23, G25, G26, G20	B
Johnson et al, 2011[i,82]	US	Claims	NR[h]	332.0	≥2 Inpatient or outpatient Dx ≥1 d apart OR<br>≥1 Inpatient or outpatient Dx + ≥1 Rx	1. Age <18 y or >64 y<br>2. Rx of antipsychotics during study period<br>3. Dx in 1 y prior to or 1 y after index PD Dx: dementia, Alzheimer's disease, schizophrenia, bipolar disorder, psychosis<br>4. Claims 1 y prior to or 1 y after index date:	B

© 2026 Esper CD et al. *JAMA Network Open*.

Source	Country	Data source type(s)[a,b]	Data source(s)	ICD (or other) code(s)	Case definition selected or used	Exclusion criteria	Case definition group[c]
						ambulatory assistance devices or long-term care	
Jones et al, 2012[83]	Canada	Claims; OA	CIHI Hospital Separation Abstracts; Physician Claim File; BC PharmaCare File	332, 332.0	≥1 Inpatient Dx OR ≥2 outpatient Dx (in any 3 y period) OR ≥1 Rx lasting ≥1 y	1. Age <65 y<br>2. Inpatient DX: 332.1	C
Kab et al, 2017[84]	France	Claims; OA	SNIRAM; PMSI	NU	≥1 Rx	1. Age <20 y<br>2. Women <50 y reimbursed for bromocriptine alone<br>3. Rx: anticholinergics, neuroleptics	Drug tracer
Kalilani et al, 2019[85]	US; UK	Claims; OA	MarketScan (commercial, Medicare); CPRD; HES	*Selected case definitions 1 and 2:* 332.0, G20.XX	*Selected case definition 1 (for MarketScan data):* ≥1 inpatient Dx OR ≥2 outpatient Dx ≥30 days apart and within 365 days of each other OR ≥1 outpatient Dx + ≥2 Rx within 6 mo of PD Dx<br>*Selected case definition 2 (for CPRD and HES data):* ≥1 inpatient Dx OR Primary care read code with PD Dx on primary care consultation date OR Primary care read code with neurologist Dx on primary consultation date	*Selected case definitions 1 and 2:*<br>1. Age <30 y<br>2. In baseline period, Dx: sec PD or pkism (including DIP, VasP, ET, NMS, postencephalitic pkism, syphilitic pkism), dementia<br>3. Rx of specific antipsychotics, metachlopramide, reserpine, amiadarone, or cinnarizine within 180 d of index Dx and follow-up period of less than 90 d	*Selected case definitions 1 and 2:* A
Kaltenboeck et al, 2012[86]	US	Claims	Medicare	332.0	≥2 Inpatient or outpatient Dx in 2 different quarters	Age <65 y	C
Kasamo et al, 2019[87]	Japan	Claims	JMDC	G20	≥1 Inpatient or outpatient Dx + ≥1 inpatient or outpatient Dx in 12 mo from index Dx	1. Age <21 y or >50 y<br>2. Dx of G21<br>3. Rx of antipsychotics	B
Kim et al, 2021[88]	US	Facility-reported	NHDS	332,0, 331.82, 333.0	1 Inpatient Dx	NU	E

© 2026 Esper CD et al. *JAMA Network Open*.

Source	Country	Data source type(s)[a,b]	Data source(s)	ICD (or other) code(s)	Case definition selected or used	Exclusion criteria	Case definition group[c]
Konings et al, 2023[89]	US	OA	TriNetX Analytics Research Network	G20	≥1 Amb Dx + ≥2 Rx	1. Age <50 y and >90 y<br>2. 1 Dx: G21, G21.1, F20-F29, F01-F03	B
Kostev et al, 2023[90]	German y	OA	Germany Disease Analyzer Database	G20	≥1 Outpatient Dx (primary care)	Age <18 y	C
Kowal et al, 2013[91]	US	Survey	MEPS; NNHS	332.X	≥1 Outpatient Dx (MEPS) OR ≥1 inpatient Dx (NNHS)	NU	D
Krzyzanowski et al, 2023[92]	US	Claims	Medicare	332, 332.0	≥1 Inpatient or outpatient Dx	1. Age <66 y or >90 y<br>2. Dx of 333.0 or 331.82 if prior to PD Dx	D
Lee et al, 2016[93]	Taiwan	Claims	NHIRD	332.0	≥2 Inpatient or outpatient Dx (first and last Dx ≥90 d apart) + ≥3 Rx after index Dx	1. Age <35 y<br>2. Dx of 332.1<br>3. Within 180 d prior to index PD Dx, any neuroleptic Rx<br>4. Dx prior to index Dx: 290, 331	A
Lee SE et al, 2018[94]	South Korea	Claims	NHIS	G20	≥2 Outpatient Dx OR ≥1 inpatient Dx	Age <30 y	C
Lee SH et al, 2018[95]	South Korea	Claims	NHIS NSSC	G20	≥1 Inpatient or outpatient Dx + ≥1 Rx for ≥3 mo	1. Age <40 y<br>2. Dx: G21-G23	A
Lien et al, 2017[96]	Taiwan	Claims	NHIRD LHID	332.0	≥3 Outpatient Dx + ≥1 Rx for ≥60 d	1. Age <50 y<br>2. Dx prior to index PD Dx: 430-434, 334, 331.3, 331.4	C
Lin et al, 2016[97]	Taiwan	Claims	NHI	332.0, 332.1	≥2 Outpatient Dx (with first and last visits ≥3 mo apart in 1 y) + ≥1 Rx for ≥60 d	NU	D
Lin et al, 2018[98]	Taiwan	Claims	NHIRD LHID	332.0	≥1 Inpatient or outpatient Dx	1. Age <20 y<br>2. Dx: 332.1	B
Lin et al, 2019[99]	Taiwan	Claims	NHIRD	332.0	≥1 Inpatient or outpatient Dx + ≥1 Rx	1. Age <20 y<br>2. Dx prior to index: stroke, dementia	B
Liu B et al, 2016[100]	Sweden	OA	MGR; Swedish	332.0, G20	≥1 Inpatient or outpatient Dx	Age <40 y	C

© 2026 Esper CD et al. *JAMA Network Open*.

Source	Country	Data source type(s)[a,b]	Data source(s)	ICD (or other) code(s)	Case definition selected or used	Exclusion criteria	Case definition group[c]
			Patient Register				
Liu, C.C.et al, 2016[101]	Taiwan	Claims	NHI	332.0	≥3 Inpatient or outpatient Dx + ≥3 Rx after first PD Dx during 7 y study period + first and last inpatient/outpatient Dx separated by ≥90 d	1. Age <40 y<br>2. Dx of 332.1<br>3. Any neuroleptic Rx within 180 before index Dx<br>4. ≥3 Inpatient or outpatient Dx of 290, 331 prior to index date	A
Liu, W.M. et al, 2016[102]	Taiwan	Claims	NHI	332.0	≥1 Inpatient or outpatient Dx (neurologist) + ≥1 Rx in each calendar year of 8 y study period	Dx at time of or ≤1 y before index PD Dx: dementia, cerebrovascular disease, head trauma, psychotic disorders	A
Liu et al, 2017[103]	US	Claims	MarketScan (commercial)	332<br>*CPT codes for DBS:* 61850, 61860, 61863, 61864, 61867, 61868, 61870, 61875, 61885, 61886, 61888, 64573, 64580, 95970- 95975, 95978, 95979	≥2 of:<br>1. 1 Inpatient or outpatient Dx (primary or secondary)<br>2. 1 Rx<br>3. DBS surgery	Age <40 y	D
Lix et al, 2010[104]	Canada	Claims; OA	Manitoba Centre for Healthy Policy Research Data Repository	332, G20	NR (referenced Guttman et al, 2003)	Age <25 y	D
Lusk et al, 2023[105]	US	Linked Claims + EHR	DUHS; Medicare	332, 332.0, G20	≥1 Encounter Dx or Billing Dx (inpatient, outpatient, or home healthcare) <u>OR</u> ≥1 Rx in prescribing or dispensing tables	Age <50 y	D
Maclagan et al, 2023[106]	Canada	Claims	OHIP	332	≥3 Outpatient Dx ≥30 d apart (all in 2 y)	Age <40 y	D

© 2026 Esper CD et al. *JAMA Network Open*.

Source	Country	Data source type(s)[a,b]	Data source(s)	ICD (or other) code(s)	Case definition selected or used	Exclusion criteria	Case definition group[c]
Madubata et al, 2015[107]	US	Claims	MarketScan (commercial)	332.0	≥2 Inpatient or outpatient Dx ≥30 d apart	NU	C
Mantri et al, 2019[108]	US	Claims	Medicare	332, 332.0	≥1 Inpatient or outpatient Dx	1. Age <65 y<br>2. Dx: 332.1, 333.0	B
Maxwell et al, 2022[109]	Canada	Claims	OHIP	*OHIP code:* 332	≥3 Outpatient Dx ≥30 d apart (all in 2 y)	Age <40 y or >85 y	B
Moisan et al, 2011[110]	France	Claims	MSA in 5 French Districts	NU	≥1 Rx in 1 y <u>OR</u> PD disease duration of ≤15 y if receiving free healthcare for PD	1. Age <18 y<br>2. Free healthcare for dementia or psychiatric disease + confirmation of related drugs<br>3. Rx of small doses of dopamine agonists for restless leg syndrome<br>4. Rx discontinued after ≤1 mo<br>5. Documented history of DIP	Drug tracer
Moisan et al, 2016[111]	France	Claims	National Health Insurance	NU	Identification based on: cumulative dose or ever use of Rx in 1 y, proportion of time treated, number of neurology/PC visits, and sex	1. Age <20 y<br>2. Females age <50 y with Rx for bromocriptine only<br>3. Rx for anticholinergics or neuroleptics only	Drug tracer
Muzerengi et al, 2017[112]	UK	Linked Claims + EHR	SUS; PICS	G20, F02.3	≥1 Inpatient Dx <u>OR</u> ≥1 Rx	1. Age 40 y<br>2. PD Rx with diagnosis other than PD	B
Nerius et al, 2017[113]	Germany	Claims	AOK	*Selected case definitions 1 and 2:* G20.0-G20.2, G20.9	*Selected case definition1:* 1 inpatient or outpatient Dx + (1 inpatient or outpatient Dx in another quarter <u>OR</u> 1 inpatient or outpatient Dx from a different physician in the same quarter)<br>*Selected case definition 2:* 1 inpatient or outpatient Dx + ≥1 Rx during entire follow-up time period	*Selected case definitions 1 and 2:*<br>1. Age <50<br>2. Most recent Dx is for an atypical pkism condition	*Selected case definitions 1 and 2:* B
Noyes et al, 2007[114]	US	Claims	Medicare	332.0, 332.1, 333.0, 333.1	≥1 PHY, outpatient, DME, inpatient, DNF, HHA, HP Dx in 1y	Age <65 y	E

© 2026 Esper CD et al. *JAMA Network Open*.

Source	Country	Data source type(s)[a,b]	Data source(s)	ICD (or other) code(s)	Case definition selected or used	Exclusion criteria	Case definition group[c]
Okunoye et al, 2021[115]	UK	OA	THIN	Read codes for PD[d]	≥1 Inpatient or outpatient Dx + ≥ 2 Rx	Age <50 y	C
Okunoye et al, 2022[116]	UK	OA	THIN	*Dx Read codes:* F11x900, F12..00, F12z.00, F130300, A94y100, F123.00, F12X.00, F120300, F13z300 *Symptom read codes:* 2944.00, 2944.11, 297A.00, 2987.00, 2987.11, 2994.00, 2994.1	≥1 Inpatient or outpatient Dx OR ≥1 symptom Read code OR ≥ 1 Rx	Age <50 y	D
Orayj et al, 2021[117]	Wales	OA	SAIL database	*Read codes*: F12..00, F120.00, F12z.00, 147F.00	≥1 Inpatient or outpatient Dx + started taking PD Rx in study period	1. Age <40 y 2. 1 y prior to PD Dx, Rx of antipsychotic	B
Pearson et al, 2023[118]	US	Claims	Medicare	G20	≥1 Inpatient or outpatient Dx	NU	C
Peterson et al, 2020[119]	US	OA	REP	332, 332.0, G20 *HICDA codes:* 03420110, 03420111, 03420112	≥2 Inpatient or outpatient Dx >30 d apart OR PD on death certificate	NU	D

© 2026 Esper CD et al. *JAMA Network Open*.

Source	Country	Data source type(s)[a,b]	Data source(s)	ICD (or other) code(s)	Case definition selected or used	Exclusion criteria	Case definition group[c]
Pou et al, 2022[120]	Spain	EHR	SIDIAP	NR[d]	≥1 Outpatient Dx (new) OR 1 new Rx	Age <40 y	C
Prada et al, 2019[121]	Colombi a	Claims	Two HMOs	G20, F02.3	≥2 Inpatient Dx (primary or secondary) OR ≥1 inpatient Dx (primary or secondary) + ≥2 Rx OR ≥2 Rx	1. Age <30 y<br>2. Rx for DIP inducing medications<br>3. Levodopa Rx with non-PD conditions	B
Pupillo et al, 2016[122]	Italy	OA	Longitudinal patient database from the Italian College of General Practitioners and Primary Care	332.0	1 Outpatient Dx	1. Previous Dx: 191, 237.5, 237.6, 239.6<br>2. Rx: antipsychotic drugs, tetrabenazine, alpha-methyl-dopa<br>3. ≥2 strokes or TIA's before or after PD Dx<br>4. Alzheimer's Dx more than 10 y before than PD Dx	B
Richy et al, 2013[123]	US	Claims	PharMetrics (commercial)	332.X	≥2 Inpatient or outpatient Dx + ≥1 Rx in the most recent 12 mo. In follow-up period	1. Age <18 y<br>2. ≥50 PD-related tablets prescribed per day	D
Riedel et al, 2016[124]	German y	Claims	German Pharmacoepid emiological Research Database	G20	≥1 Inpatient Dx (primary or secondary) in 1 y OR ≥1 outpatient Dx ("certain" PD Dx) + (≥1 inpatient or outpatient Dx or ≥1 Rx) within 12 mo OR Rx in 1 y + inpatient or outpatient Dx within 12 mo	1. Age <65 y<br>2. Dx of G21<br>3. Inpatient admission or outpatient Dx or "suspected," "status post," or "condition excluded" of PD	B
Schmitz et al, 2022[125]	Luxemb ourg	OA	CNS database	NU	≥2 Rx within 12 mo of each other	1. Age <50 y<br>2. Rx: neuroleptics (except quetiapine and clozapine), metoclopramide, cinnarzine, flunarizine<br>3. Rx with ATC code N04B<br>4. Rx: bromocriptine, lisuride	Drug Tracer

© 2026 Esper CD et al. *JAMA Network Open*.

Source	Country	Data source type(s)[a,b]	Data source(s)	ICD (or other) code(s)	Case definition selected or used	Exclusion criteria	Case definition group[c]
Schrag et al, 2015[126]	UK	OA	THIN	Read codes for PD[d]	≥1 Outpatient Dx + ≥2 Rx	1. Age <50 y<br>2. 1 Dx: sec pkism, schizophrenia<br>3. Prior to PD Dx, 1 Dx of dementia<br>4. Use of specific neuroleptic Rx	B
Scott et al, 2023[127]	US	OA	VHA	332.0, G20	**e**≥1 Inpatient or outpatient Dx + ≥2 Rx	Age <40 y	C
Searles Nielsen et al, 2017[128]	US	Claims	Medicare	332, 332.0	≥1 Inpatient or outpatient Dx	1. Age >90 y<br>2. Previous Dx: 331.82, 333, 333.0, 322, 322.0 (without CPT 62270)	D
Seki et al, 2023[129]	Japan	Claims	Japan Elderly Database; JDMC; NHI database	G20	≥1 Inpatient or outpatient Dx + additional Dx's for ≥6 mo from index date + ≥2 Rx ≥6 mo from index date	1. Age <30 y<br>2. Dx ≤6 mo prior to index date: DIP, cerebrovascular pkism	A
Seo et al, 2021[130]	South Korea	Claims	NHIS	V124	≥1 Inpatient or outpatient Dx	1. Age <40 y<br>2. Dx: G21, G22, G23<br>3. Prescribed antiparkinsonism Rx for total of <180 d	B
Shin et al, 2018[131]	Canada	OA	ONPHEC	332.0-.1, G20, G21.0-.4, G21.8-.9, G22, F02.3	≥2 Outpatient Dx in 1 y <u>OR</u><br>≥1 Rx + ≥1 outpatient Dx within 6 mo of each other	Age <55 y or > 85 y	D
Song et al, 2023[132]	US	Claims	Medicare	332.0, G20	≥2 Inpatient or outpatient Dx ≥1 mo apart	Persons <65 y at time of first Dx	C
Straif-Bourgeois et al, 2015[133]	US	OA	LAHIDD	332, 332.0	1 Inpatient Dx	1 Inpatient Dx: 332.1	C
Swarztrauber et al, 2005[134]	US	OA	VHA: PBM; CHIPS	332.0, 332.1, 333.0	≥1 Inpatient or outpatient Dx <u>OR</u> ≥ 1 Rx	NU	E

© 2026 Esper CD et al. *JAMA Network Open*.

Source	Country	Data source type(s)[a,b]	Data source(s)	ICD (or other) code(s)	Case definition selected or used	Exclusion criteria	Case definition group[c]
Swarztrauber et al, 2006[135]	US	OA	VHA	332.0, 333.0	≥1 Outpatient Dx + ≥1 Rx	NU	D
Szatmari et al, 2019[136]	Hungary	Claims	NEUROHUN	G20	≥2 Inpatient or outpatient Dx (minimum 1 Dx in ≥2 of 10 y)	Dx or Rx (with associated Dx): G21-G26	A
Szumski et al, 2009[137]	US	OA	VHA	332.0	≥2 Outpatient Dx by the highest specialist in record	1. Movement disorders specialist Dx other than 332.0<br>2. ≥2 neurologist or non-neurologist Dx other than 332.0	A
Thacker et al, 2016[138]	US	EHR	One institution in Albuquerque, NM	332, 332.0	1 Inpatient or outpatient Dx (primary Dx only)	Age <18 y	D
Ton et al, 2010[139]	US	Survey; Claims	Cardiovascular Health Survey; Medicare	332.0	≥1:<br>1.1 Inpatient Dx<br>2.1 Rx<br>3.Self-report PD Dx	Age <65 y	C
Valent et al, 2018[140]	Italy	OA	FVG databases	332.XX	≥1 Inpatient Dx OR medical charge exemption code for PD OR HC for PD OR nursing home admit for PD OR ≥3 Rx for ≥6 mo OR ≥1 Inpatient Dx (All above in 1 y)	Rx ATC codes N05xxxx	C
Van de Vijver et al, 2001[141]	The Netherlands	OA	PHARMO system	NU	1 Rx (specific medications have different weights)	1. Age <55 y<br>2. Rx of anticholinergic only<br>3. Rx of anticholinergic + antipsychotics only<br>4. Received inpatient discharge and did not fill PD Rx in 1 d after	Drug tracer
Van den Eeden et al, 2003[142]	US	OA; Other	KPMCP databases	332.0, 331.0, 333.0, 332.X	1 Inpatient or outpatient Dx OR PD patient referral OR PD patient from neurologist registry	NU	E

© 2026 Esper CD et al. *JAMA Network Open*.

Source	Country	Data source type(s)[a,b]	Data source(s)	ICD (or other) code(s)	Case definition selected or used	Exclusion criteria	Case definition group[c]
Vlaar et al, 2018[143]	France	Claims	SNIRAM	NU	≥1 Rx reimbursement	1. Age <20 y<br>2. Women age <50 y with Rx reimbursement for bromocriptine<br>3. Only on Rx: anticholinergics or neuroleptics	Drug tracer
Wada-Isoe et al, 2023[144]	Japan	Claims	MDV	G20	≥1 Inpatient or outpatient Dx (definitive) +<br>≥1 Rx + ≥1 inpatient or outpatient Dx (definitive, with PD identified)	1 definitive Dx: Parkinson's syndrome, juvenile Parkinson's syndrome, unilateral Parkinson's syndrome, Parkinson's disease dementia, familial Parkinson's disease, familial Parkinson's disease Yahr I-V	C
Wang et al, 2011[145]	Taiwan	Claims	NHIRD	332	≥1 Inpatient or outpatient Dx + ≥2 Rx	1. Use of PD Rx in first y of study period only<br>2. Only 1 PD Rx<br>3. In 1 y prior to cohort entry, use of antipsychotic Rx or Dx of 295	C
Wei et al, 2013[146]	US	Claims	Medicare	332.0	≥1 Inpatient or outpatient Dx in each of two study period y	Age <65 y	C
Wei et al, 2015[147]	US	Claims	Medicare	332.0	≥1 Inpatient or outpatient Dx (in each of two study period y) + ≥2 Rx	Age <65 y	C
Wei et al, 2016[148]	US	Linked Claims + EHR	VUMC EHR	332.0	≥2 of:<br>1. Outpatient Dx<br>2. Rx<br>3. PD in clinical notes	NU	C
Weimers et al, 2019[149]	Sweden	OA	NPR; Prescribed Drug Register; Total Population Register; Multigeneratio n Register	332.0, G20	≥1 Inpatient or outpatient Dx	NU	D

© 2026 Esper CD et al. *JAMA Network Open*.

Source	Country	Data source type(s)[a,b]	Data source(s)	ICD (or other) code(s)	Case definition selected or used	Exclusion criteria	Case definition group[c]
Weintraub et al, 2016[150]	US	OA	VHA	332.0	≥1 Inpatient or outpatient Dx	1. Dx of 331.82 2. Dementia Dx prior to or within 1 y of PD Dx 3. Antipsychotic Rx in 180 d prior to PD Dx 4. Age <50 y at initiation of antipsychotic therapy 5. Dx: bipolar disorder, schizophrenia, schizoaffective disorder, Huntington's disease	B
Weir et al, 2018[151]	UK	Claims; OA	HES; CPRD	G20.X	≥1 Inpatient or outpatient Dx + ≥2 Rx	1. Age <30 y 2. At any point in record, Dx: sec pkism, Parkinson's Plus syndromes[f] 3. Evidence of exposure to agents known to produce parkinsonian syndromes	B
Wetmore et al, 2019[152]	US	Claims	Medicare	332.0x	≥2 Inpatient or outpatient Dx ≥30 d apart within 1 y	1. Age <40 y 2. At any time during study period ≥2 Dx for putative dementia etiologies more specific than PD 3. After PD Dx, ≥1 Dx: 290.1-290.3 4. At any time during study period, ≥1 Dx chronic psychiatric disease 5. Before PD Dx, ≥1 Dx: alcohol-induced psychotic, delusional disorders	A
White et al, 2007[153]	US	OA	MEDVAC	332.0	≥1 Inpatient or outpatient Dx	Dx: 332.1	B
Williamson et al, 2014[154]	Canada	OA	CPCSSN	332.*	Chart terms: "Parkinson's disease," "paralysis agitans," "parkinsonism"	1. Age <60 y 2. Chart terms: "tremor," "Wolf-Parkinson-White syndrome," and	D

© 2026 Esper CD et al. *JAMA Network Open*.

Source	Country	Data source type(s)[a,b]	Data source(s)	ICD (or other) code(s)	Case definition selected or used	Exclusion criteria	Case definition group[c]
					OR PD billing code OR problem list/encounter list Dx OR Rx + (billing code or outpatient Dx)	"suspected" or "possible" variations of the inclusion criteria	
Willis et al, 2010[155]	US	Claims	Medicare	332, 332.0	≥1 Inpatient or outpatient Dx	1. Age <65 y<br>2. Dx: 332.1, 333.0	B
Willis et al, 2011[156]	US	Claims	Medicare	332.0	≥2 Outpatient Dx	1. Age <65 y<br>2. Dx after PD Dx: sec pkism, atypical parkinsonian syndrome[f]	B
Xu et al, 2023[157]	US	Survey	NHANES	NU	Yes to:<br>1. "In the past 30 days, have you used or taken medication for which a prescription is needed?"<br>2. Use of specific medications NHANES identified as associated with PD	Age <50 y	E
Yang et al, 2017[158]	Taiwan	Claims	NHIRD	332.0	≥3 Outpatient Dx OR ≥1 inpatient Dx	Age <20 y	C
Yuchi et al, 2020[159]	Canada	Claims	MSP; PharmaNet	332	≥2 Outpatient Dx in 1 y OR ≥1 Rx + ≥1 outpatient Dx within 6 mo of each other	Age <45 y and >84 y	D
Zenesini et al, 2023[160]	Italy	Claims; OA	BLHT	332.0<br>*Copayment exemption code:* 038.332	≥1 of the following in 1 y:<br>1. ≥HD Dx (primary or secondary Dx)<br>2. ≥1 copayment exemption code<br>3. ≥2 Rx for ≥180 d in 1 y	1 HD Dx of 332.1 (if using HD Dx to include as a case)	C

Abbreviations: Amb, ambulatory; AOK, Allgemeine Ortskrankenkasse; APD, antiparkinsonian drug; ATC, Anatomic Therapeutic Chemical; BASF, Beneficiary Annual Summary File; BC, British Columbia; BCLHD, British Columbia Linked Health Database; BHLT, Bologna health administrative; CA, California; CCRS, Continuing Care Reporting System; CCW, Chronic Conditions Warehouse; CDR, Cause of Death Register; CDW, Corporate Data Warehouse; CHIPS, Consumer Health Information and Performance Sets; CHS, Clalit Health Services; CIHI, Canadian Institute of Health Information; CNS, Caisse nationale de santé; CPCSSN, Canadian Primary Care Sentinel Surveillance Network; CPRD, Clinical Practice Research Database; CPT, current procedural terminology; DAD, Discharge Abstract Database; DIP, drug-induced parkinsonism; DME, durable medical equipment; Dx, diagnostic claim; DBS, deep brain stimulation; DLB, dementia with Lewy bodies; DUHS: Duke University Health System; EGB, L'Enchantillon généraliste de bénéficiaries; EHR, electronic health records; ET, essential tremor; FHS, Framingham Hearty Study; FL, Florida; FVG, Friuli-Venezia Giulia; GPRD; General Practice Research Database; HBCCF, hospital-based community care

© 2026 Esper CD et al. *JAMA Network Open*.

facilities; HC, home care; HCRS, Home Care Reporting System; HCUP, Healthcare Cost and Utilization Project; HD, hospital discharge; HES, Hospital Episode Statistics; HHA, home health; HICDA, Hospital Adaptation of the International Classification of Diseases; HIRA, Health Insurance Review and assessment; HMO, health maintenance organization; HP, hospice; ICPC, International Classification of Primary Care; IHS, Indian Health Services; IL, Illinois; JMDC, JMDC Inc. (Japanese insurance company); KPMPC: Kaiser Permanente Medical Care Program; LHID, Longitudinal Health Insurance Database; LTC, long-term care; MA, Medicare Advantage; MAPD, Medicare Advantage and Prescription Drug; MDV, Medical Data Vision; MEDVAC, Michael E. Debakey Veterans Affairs Medical Center; MEPS, Medical Expenditure Panel Survey; MGR, Multi-Generation Register; MIA, Medical Information Analysis; MSA, Mutualité Sociale Agricole; MSP, Medical Services Plan; NACRS, National Ambulatory Care Reporting System; NAMCS, National Ambulatory Medical Care Survey; NHAMCS, National Hospital Ambulatory Medical Care Survey; NHDS, National Hospital Discharge Survey; NHI, National Health Insurance; NHIP, National Health Insurance Program; NHIRD, National Health Insurance Research Database; NHIS, National Health Information System; NHSP, National Health Screening Program; NJ, New Jersey; NMS, Neuroleptic Malignant Syndrome; NNHS, National Nursing Home Survey; NorPD, Norway Prescription Database; NOS, not otherwise specified; NPR, National Patient Register; NR, not reported; NRRHS, National Registry of Reimbursed Health Services; NU, Not used; NUDB, National Education Database; OA, other administrative (see footnote e for more information); ODB, Ontario Drug Benefit; OH, Ohio; OHIP, Ontario Health Insurance Plan; NM, New Mexico; OMHRS, Ontario Mental Health Reporting System; NSSC, National Sample Cohort; NY, New York; OMHRS, Ontario Mental Health Reporting System; ONPHEC, Ontario Population Health and Environment Cohort; pkism, parkinsonism; OXMIS, Oxford Medical Information System; PA, Pennsylvania; PBM, Pharmacy Benefits Manager; PC, primary care; PD, Parkinson disease; PDD, Patient Discharge Database; PHY, Physician/Carrier; PICS, Prescribing Information and Communication Services; PMSI, Programme de Médicalisation de Systèmes d'Information; REP, Rochester Epidemiology Project; RPDB, Registered Persons Database; Rx, pharmaceutical claim (in this context, pharmaceutical claim for a PD medication specified by the article); SAIL, Secure Anonymized Information Linkage; SDS, same day surgery; sec pkism, secondary parkinsonism; SHI, Statutory Health Insurance; SIDIAP, Information System for the Development of Research in Primary Care; SNF, Skilled Nursing Facility; SNIRAM, Système National d'Information Inter-Régimes de l'Assurance Maladie; SUS, Secondary Uses Services; THIN, The Health Improvement Network; TIA, transient ischemic attack; UK, United Kingdom; VasP, vascular parkinsonism; VHA, Veterans Health Administration; VSD, Vital Statistics Database; VUMC, Vanderbilt University Medical Center.

[a] Administrative data was split into two categories: claims and other administrative. Other administrative data source types include medical or billing information that are not claims-based (eg, aggregated information pulled from EHR for research or administrative purposes).

[b] EHR and EMR (electronic medical records) are both referred to as EHR.

[c] Please see Table 2 for more information on case definition groups.

[d] Specific codes used not specified.

[e] Time difference between the date of the last dispensed medication and the date of death/end of record period.

[f] Atypical parkinsonisms, atypical parkinsonian syndrome, and Parkinson's Plus syndromes are other terms for other neurodegenerative parkinsonisms and include dementia with Lewy bodies, corticobasal degeneration/corticobasal syndrome, progressive supranuclear palsy, and multiple system atrophy.

[g] Danila et al, 2014 utilized index assessment data from the included data sources, which is a binary variable that identifies if an individual has or does not have a condition of interest based on the most recent assessment of persons within the study cohort.

[h] Holt et al, 2010 did not report the specific data source used but described it as "a de-identified health care claims database from a large United States managed care population…"[76]

[i] Johnson et al, 2011 did not specify the data source(s) used; however, they identify, "Patients with PD were selected from combined de-identified administrative health insurance and disability claims of 2.3 million employed beneficiaries from 55 large, self-insured US companies from different geographic regions and industries."[82]

© 2026 Esper CD et al. *JAMA Network Open*.

eTable 4. Interrater Reliability of Quality Assessment of Validation Articles Using Modified QUADAS-2 Tool

QUADAS-2 question	Percent agreement (%) (No. = 18[b])	Kappa[a] (95% CI) (No. = 18[b])
Domain 1: Participant selection		
Risk of bias	33.3	-0.1 (-0.5-0.2)
Applicability	55.6	0.1 (-0.4-0.5)
Doman 2: Index test		
Risk of bias	83.3	0.2 (-0.1-0.5)
Applicability	66.7	0.2 (-0.2-0.6)
Domain 3: Reference standard		
Risk of bias	83.3	0.7 (0.4-1.0)
Applicability	88.9	0.8 (0.4-1.0)
Domain 4: Flow and timing		
Risk of bias	83.3	0.7 (0.4-1.0)

Abbreviations: CI, confidence interval; QUADAS-2, Quality Assessment of Diagnostic Accuracy Studies-2.
[a] Calculated using Cohen's Kappa.
[b] Of the 23 validation studies identified in this review, five were used for training, leaving 18 for interrater reliability calculations.

© 2026 Esper CD et al. *JAMA Network Open*.

eTable 5. Risk of Bias and Applicability Concerns in Included Validation Studies Identified Using Modified QUADAS-2 Tool

	Risk of bias					Applicability concerns			
Source	Patient selection	Index test	Reference standard	Flow and timing	Concern?	Patient selection	Index test	Reference standard	Concern?
Baldacci et al, 2015[10]	H	L	H	H	Yes	H	H	L	Yes
Butt et al, 2014[21]	H	L	L	L	Yes	H	L	L	Yes
Chillag-Talmor et al, 2011[38]	H	L	H	L	Yes	H	L	L	Yes
Feldman et al, 2012[61]	L	L	L	L	No	L	L	L	No
Gordon et al, 2013[68]	H	L	L	H	Yes	H	L	L	Yes
Hernán et al, 2006[74]	H	L	H	U	Yes	H	L	L	Yes
Hill et al, 2023[75]	H	L	H	L	Yes	H	L	L	Yes
Jain et al, 2015[80]	L	L	L	L	No	H	Varies by index test[a]	L	Varies by index test[a]
Lee et al, 2016[93]	H	L	H	H	Yes	H	L	U	Yes
Liu et al, 2016[101]	H	L	H	H	Yes	H	L	U	Yes
Lusk et al, 2023[105]	L	L	H	L	Yes	L	L	H	Yes

© 2026 Esper CD et al. *JAMA Network Open*.

Source	Risk of bias: Patient selection	Risk of bias: Index test	Risk of bias: Reference standard	Risk of bias: Flow and timing	Risk of bias: Concern?	Applicability concerns: Patient selection	Applicability concerns: Index test	Applicability concerns: Reference standard	Applicability concerns: Concern?
Noyes et al, 2007[114]	L	L	H	L	Yes	L	L	L	No
Peterson et al, 2020[119]	L	L	L	L	No	L	L	L	No
Scott et al, 2023[127]	L	L	L	L	No	H	H	L	Yes
Swarztrauber et al, 2005[134]	L	L	L	L	No	H	L	L	Yes
Swaztrauber et al, 2006[135]	H	L	L	H	Yes	H	L	L	Yes
Szatmari et al, 2019[136]	L	L	H	H	Yes	L	L	L	No
Szumski et al, 2009[137]	H	L	L	L	Yes	H	L	L	Yes
Thacker et al, 2016[138]	H	L	H	H	Yes	H	L	L	Yes
Wei et al, 2016[148]	H	L	H	U	Yes	H	L	L	Yes
White et al, 2007[153]	H	L	L	L	Yes	H	L	L	Yes
Williamson et al, 2014[154]	L	U	L	L	Yes	L	U	L	Yes
Zenesini et al, 2023[160]	H	L	L	L	Yes	H	H	L	Yes

© 2026 Esper CD et al. *JAMA Network Open*.

Abbreviations: H, High risk of bias or applicability concern in domain indicated; L, low risk of bias or applicability concern in domain indicated; QUADAS-2, Quality Assessment of Diagnostic Accuracy Studies-2; U, unclear risk of bias or applicability concern in domain indicated.
[a] Jain et al 2015 reported on three index tests. Concerns about the applicability of the index tests and overall results associated with that test are as follows:1) Medicare-based: Low, No; 2) Prescription-based: High, Yes; and 3) Self-report-based: High, Yes.[80]

© 2026 Esper CD et al. *JAMA Network Open*.

eTable 6. Interrater and Intrarater Reliability of Quality Assessment of Nonvalidation Articles Using Modified MORE Tool

MORE question	Interrater reliability (No. = 53) Percent agreement (%)	Interrater reliability (No. = 53) Kappa[a] (95% CI)	Intrarater reliability Reviewer 1 (No. = 10) Percent agreement (%)	Intrarater reliability Reviewer 1 (No. = 10) Kappa[a] (95% CI)	Reviewer 2 (No. = 9) Percent agreement (%)	Reviewer 2 (No. = 9) Kappa[a] (95% CI)
1. Funding	78.8	0.7 (0.6-0.9)	90.0	0.9 (0.6-1.0)	66.7	0.6 (0.2-0.9)
2. Conflict of interest	92.3	0.9 (0.7-1.0)	100	1.0 (1.0-1.0)	100	1.0 (1.0-1.0)
3. Ethical approval	90.4	0.8 (0.6-1.0)	100	1.0 (1.0-1.0)	88.9	0.7 (0.2-1.0)
4. Study aim	100.0	1.0 (1.0-1.0)	100	1.0 (1.0-1.0)	100	1.0 (1.0-1.0)
5. Study design	73.1	0.6 (0.4-0.8)	90.0	0.9 (0.6-1.0)	66.7	0.6 (0.2-0.9)
6. Sampling method	78.8	0.6 (0.3-0.8)	90.0	0.8 (0.5-1.0)	77.8	0.6 (0.1-1.0)
7. Sampling design	86.5	0.8 (0.7-0.9)	100	1.0 (1.0-1.0)	88.9	0.8 (0.5-1.0)
8. Sampling bias	96.2	0.6 (0.2-1.0)	100	1.0 (1.0-1.0)	100	1.0 (1.0-1.0)
9. Case identification method	90.4	0.3 (-0.2-0.7)	100	1.0 (1.0-1.0)	88.9	0 (0-0)
10. Validation of case identification method	55.8	0.3 (0.1-0.5)	90.0	0.8 (0.3-1.0)	77.8	0.7 (0.4-1.0)

Abbreviations: CI, confidence interval; MORE, Methodological Evaluation of Observational Research.
[a] Calculated using Cohen's Kappa.

© 2026 Esper CD et al. *JAMA Network Open*.

eTable 7. Poor Reporting and Bias Issues Identified in Included Nonvalidation Literature Using Modified MORE Tool

	Quality issues identified per modified MORE question[a]									
Source	Funding	Conflict of interest	Ethical approval	Study aim	Study design	Sampling method	Sampling design	Sampling bias	Case identification method	Validation of case identification method
Aamodt et al, 2021[5]									Minor	PR
Aamodt et al, 2023[6]									Minor	PR
Abuhasira et al, 2019[7]									Minor	
Albarmawi et al, 2022[8]									Minor	PR
Alonso et al, 2009[9]									Minor	
Barer et al, 2022[11]									Minor	PR
Becker et al, 2008[12]									Minor	
Becker et al, 2011[13]		PR			PR				Minor	
Bhattacharjee et al, 2015[14]			PR				Minor		Minor	Major
Bhattacharjee et al, 2018[15]	PR								Minor	PR
Blin et al, 2015[16]									Minor	Minor
Brakedal et al, 2022[17]									Minor	PR
Brandt-Christensen et al, 2006[18]		PR	PR						Minor	PR

© 2026 Esper CD et al. *JAMA Network Open*.

	Quality issues identified per modified MORE question[a]									
Source	Funding	Conflict of interest	Ethical approval	Study aim	Study design	Sampling method	Sampling design	Sampling bias	Case identification method	Validation of case identification method
Bronskill et al, 2022[19]									Minor	
Búřil et al, 2021 [20]									Minor	PR
Callaghan et al, 2012[22]					PR		Minor		Minor	PR
Camacho-Soto et al, 2018[23]									Minor	PR
Carriere et al, 2017 [24]	PR		PR		PR				Minor	Minor
Cepeda et al, 2019[25]							Minor		Minor	
Chandler et al, 2021[26]	PR						Minor		Minor	PR
Chang et al, 2016[27]	PR								Minor	
Chekani et al, 2016[28]	PR					Major			Minor	PR
Chekani et al, 2020[29]	PR								Minor	Major
Chen et al, 2012[30]					PR				Minor	PR
Chen et al, 2015[31]	PR								Minor	PR
Chen CY et al, 2017[32]									Minor	Major
Chen H et al, 2017[33]									Minor	
Chen et al, 2018[34]	PR								Minor	PR

© 2026 Esper CD et al. *JAMA Network Open*.

	Quality issues identified per modified MORE question[a]									
**Source**	**Funding**	**Conflict of interest**	**Ethical approval**	**Study aim**	**Study design**	**Sampling method**	**Sampling design**	**Sampling bias**	**Case identification method**	**Validation of case identification method**
Chen SF et al, 2020[35]									Minor	
Chen W et al, 2020[36]									Minor	
Chen et al, 2021[37]									Minor	
Choi et al, 2019[39]									Minor	PR
Chou et al, 2017[40]									Minor	
Connolly et al, 2015[41]			PR						Minor	
Cortese et al, 2018[42]									Minor	PR
Crispo et al, 2015[43]							Minor		Minor	PR
Crispo et al, 2016[44]							Minor		Minor	PR
Crispo et al, 2020[45]									Minor	
Dahodwala et al, 2009[46]					PR				Minor	PR
Dahodwala et al, 2017[47]									Minor	
Dahodwala et al, 2020[48]									Minor	
Dammertz et al, 2023[49]	PR				PR				Minor	Minor

© 2026 Esper CD et al. *JAMA Network Open*.

Source	Quality issues identified per modified MORE question[a]								
	Funding	Conflict of interest	Ethical approval	Study aim	Study design	Sampling method	Sampling design	Sampling bias	Case identification method	Validation of case identification method
Danila et al, 2014[50]					PR				Minor	PR
De Vera et al, 2008[51]	PR	PR	PR						Minor	
DeMarco et al, 2023[52]							Minor		Minor	Minor
Doblhammer et al, 2018[53]			PR	Minor	PR	Major			Minor	Minor
Dore et al, 2009[54]			PR						Minor	
Etminan et al, 2008[55]		PR	PR						Minor	PR
Eusebi et al, 2019[56]			PR		PR				Minor	Major
Fan et al, 2019[57]									Minor	Minor
Fang et al, 2020[58]									Minor	
Faust et al, 2020[59]									Minor	Minor
Feldman et al, 2011[60]			PR					PR	Minor	
Finkelstein et al, 2007[62]		PR				Major	Major	PR	Minor	Minor
François et al, 2017[63]			PR						Minor	PR
Freedman et al, 2016[64]									Minor	Major
Fullard et al, 2018[65]									Minor	Minor

© 2026 Esper CD et al. *JAMA Network Open*.

	Quality issues identified per modified MORE question[a]									
Source	Funding	Conflict of interest	Ethical approval	Study aim	Study design	Sampling method	Sampling design	Sampling bias	Case identification method	Validation of case identification method
Gandhi et al, 2021[66]									Minor	
Gordon et al, 2012[67]	PR						Minor		Minor	PR
Gordon et al, 2013[68]	PR						Minor		Minor	PR
Guo et al, 2014[70]									Minor	PR
Guttman et al, 2003[71]		PR	PR						Minor	Major
Han et al, 2019[72]									Minor	Minor
Heinzel et al, 2018[73]									Minor	Minor
Holt et al, 2010[76]			PR				Minor	PR	Minor	PR
Horsfall et al, 2013[77]	PR								Minor	Minor
Huse et al, 2005[78]		PR	PR				Minor		Minor	PR
Iketani et al, 2020[79]									Minor	PR
Jeong et al, 2019[81]	PR				PR				Minor	PR
Johnson et al, 2011[82]			PR				Minor		Minor	PR
Jones et al, 2012[83]	PR	PR							Minor	Minor
Kab et al, 2017[84]			PR		PR				Minor	
Kalilani et al, 2019[85]									Minor	

© 2026 Esper CD et al. *JAMA Network Open*.

	Quality issues identified per modified MORE question[a]									
Source	Funding	Conflict of interest	Ethical approval	Study aim	Study design	Sampling method	Sampling design	Sampling bias	Case identification method	Validation of case identification method
Kaltenboeck et al, 2012[86]		PR	PR						Minor	Minor
Kasamo et al, 2019[87]					PR				Minor	PR
Kim et al, 2021[88]		PR			PR					PR
Konings et al, 2023[89]							Minor		Minor	
Kostev et al, 2023[90]									Minor	PR
Kowal et al, 2013[91]			PR		PR	Major			Minor	PR
Krzyzanowski et al, 2023[92]									Minor	
Lee SE et al, 2018[94]	PR				PR				Minor	PR
Lee SH et al, 2018[95]									Minor	PR
Lien et al, 2017[96]	PR								Minor	
Lin et al, 2016[97]			PR		PR				Minor	PR
Lin et al, 2018[98]									Minor	PR
Lin et al, 2019[99]									Minor	
Liu B et al, 2016[100]									Minor	Minor
Liu WM et al, 2016[102]	PR								Minor	PR

© 2026 Esper CD et al. *JAMA Network Open*.

	Quality issues identified per modified MORE question[a]									
Source	Funding	Conflict of interest	Ethical approval	Study aim	Study design	Sampling method	Sampling design	Sampling bias	Case identification method	Validation of case identification method
Liu et al, 2017[103]			PR				Minor		Minor	PR
Lix et al, 2010[104]					PR				Minor	Minor
Maclagan et al, 2023[106]									Minor	
Madubata et al, 2015[107]			PR		PR		Minor		Minor	PR
Mantri et al, 2019[108]					PR				Minor	PR
Maxwell et al, 2022[109]									Minor	
Moisan et al, 2011[110]				Minor	PR				Minor	
Moisan et al, 2016[111]			PR		PR				Minor	Minor
Muzerengi, et al 2017[112]						Major	Major	PR	Minor	PR
Nerius et al, 2017[113]	PR		PR		PR				Minor	Minor
Okunoye et al, 2021[115]									Major	
Okunoye et al, 2022[116]									Minor	
Orayj et al, 2021[117]									Minor	PR
Pearson et al, 2023[118]									Minor	PR
Pou et al, 2022[120]	PR								Minor	PR

© 2026 Esper CD et al. *JAMA Network Open*.

Source	Quality issues identified per modified MORE question[a]									
	Funding	Conflict of interest	Ethical approval	Study aim	Study design	Sampling method	Sampling design	Sampling bias	Case identification method	Validation of case identification method
Prada et al, 2019[121]			PR		PR				Minor	
Pupillo et al, 2016[122]			PR		PR				Minor	PR
Richy et al, 2013[123]			PR				Minor		Minor	PR
Riedel et al, 2016[124]					PR				Minor	PR
Schmitz et al, 2022[125]					PR				Minor	Major
Schrag et al, 2015[126]									Minor	
Searles Nielsen et al, 2017[128]									Minor	PR
Seki et al, 2023[129]									Minor	PR
Seo et al, 2021[130]									Minor	Major
Shin et al, 2018[131]									Minor	
Song et al, 2023[132]									Minor	PR
Straif-Bourgeois et al, 2015[133]	PR	PR	PR		PR		Minor		Minor	PR
Ton et al, 2010[139]		PR	PR	Minor				PR		Minor
Valent et al, 2018[140]	PR								Minor	PR

© 2026 Esper CD et al. *JAMA Network Open*.

	Quality issues identified per modified MORE question[a]									
**Source**	**Funding**	**Conflict of interest**	**Ethical approval**	**Study aim**	**Study design**	**Sampling method**	**Sampling design**	**Sampling bias**	**Case identification method**	**Validation of case identification method**
Van den Eeden et al, 2003[142]		PR			PR		Minor		Minor	PR
van de Vijver et al, 2001[141]		PR	PR		PR				Minor	PR
Vlaar et al, 2018[143]			PR		PR				Minor	Minor
Wada-Isoe et al, 2023[144]					PR				Minor	PR
Wang et al, 2011[145]									Minor	PR
Wei et al, 2013[146]									Minor	
Wei et al, 2015[147]									Minor	PR
Weimers et al, 2019[149]									Minor	PR
Weintraub et al, 2016[150]							Minor		Minor	Major
Weir et al, 2018[151]									Minor	
Wetmore et al, 2019[152]									Minor	Minor
Willis et al, 2010[155]		PR							Minor	PR
Willis et al, 2011[156]									Minor	Minor
Xu et al, 2023[157]									Major	Major

© 2026 Esper CD et al. *JAMA Network Open*.

Source	Quality issues identified per modified MORE question[a]									
	Funding	Conflict of interest	Ethical approval	Study aim	Study design	Sampling method	Sampling design	Sampling bias	Case identification method	Validation of case identification method
Yang et al, 2017[158]									Minor	Minor
Yuchi et al, 2020[159]									Minor	PR

Abbreviations: MORE, Methodological Evaluation of Observational Research; PR, poor reporting.

[a] The modified MORE tool identified three types of risks to quality flags: poor reporting (item in question is not reported in the manuscript under review), minor risk of bias, and major risk of bias. The table above presents the risks to quality flags identified per article per modified MORE question. Blank cells indicate no risk to quality issues for the corresponding modified MORE question.

© 2026 Esper CD et al. *JAMA Network Open*.

eTable 8. NNCSS's Assessment of Situational Factors to Determine Suitability of PD Case Definitions

**Can PD case definitions be differentiated and grouped by their suitability for NNCSS due to whether they are…**	
Reflective of current diagnostic criteria or treatment?	No
Routinely cited in the literature?	Limited
Used frequently?	No
Used recently?	No
Used by leaders in the field?	No
Used in different types of data sources?	Limited; Relatively few in US
Used to estimate prevalence versus for another purpose?	Limited
**Conclusion**	**No**

Abbreviations: NNCSS, National Neurological Conditions Surveillance System; PD, Parkinson Disease.

© 2026 Esper CD et al. *JAMA Network Open*.

eTable 9. *ICD-9* and *ICD-10* Coding of Parkinson Disease and Other Parkinsonisms

Condition	*ICD-9* code	*ICD-10* code
Primary/Neurodegenerative parkinsonism[c]		
Parkinson's disease	332[a], 332.0	G20[b]
Other neurodegenerative parkinsonisms	333.0	G23.X
Multiple System Atrophy	No code	G90.3
Progressive Supranuclear Palsy	No code	G23.1
Corticobasal Degeneration/Syndrome	331.6	G31.85
Dementia with Lewy Bodies	331.82	G31.82
Secondary parkinsonism[c]	332, 332.1	G21.X
Drug-induced parkinsonism	332.1	G21.1X
Vascular parkinsonism	No code	G21.4

Abbreviation: ICD, International Classification of Diseases.

[a] 332 – Parkinson's disease (PD) is not a billing code. The associated billing codes and definitions (per ICD-9 classification)[161] are:

332.0 – "paralysis agitans: includes (Parkinson's disease) PD or parkinsonism (non-specific, idiopathic, primary)."

332.1 – "secondary parkinsonism: includes drug-induced parkinsonism (DIP) (parkinsonism due to drugs), neuroleptic-induced parkinsonism."

[b] The definition of G20 per ICD-10 classification[162] is "Parkinson's disease (PD) including hemiparkinsonism, idiopathic parkinsonism or PD, paralysis agitans, parkinsonism or PD not otherwise specified (NOS), primary parkinsonism or PD."

[c] ICD code definition overlap occurs for PD and the other parkinsonisms in ICD-9 and ICD-10. For example, in ICD-10, G20 is commonly referred to as Parkinson's disease. However, based on its ICD-10 definition, G20 also includes some other parkinsonisms, despite all neurodegenerative and secondary parkinsonisms having their own ICD-10 codes. Despite this, based on G20's ICD-10 definitions, it is technically correct for a person with an other parkinsonism condition to be coded as G20.

© 2026 Esper CD et al. *JAMA Network Open*.

eResults. Additional NNCSS Case Definition Parameters

Lower Bound Age Parameter for Parkinson Disease Case Definitions

We conducted a supplemental targeted literature search and consulted with a movement disorders specialist (CDE) to establish a lower age bound for the NNCSS Parkinson disease (PD) case definitions. Although PD predominantly affects older adults,[163,164] a distinct subset of patients are impacted at an earlier age, referred to as young-onset PD. There is some discrepancy as to the age range of young-onset PD, with some experts indicating age 21 to 40 years[165-169] and a recent publication from the Movement Disorders Society Task Force on Early Onset Parkinson Disease[170] recommending age 21 to 50 years. Despite these differences, most young-onset PD cases have an age of onset closer to the upper end of the range, with one recent study identifying an average onset at 43 years.[171] Of the 103 studies in our systematic review that included age parameters and did not use data sources restricted to age 65 and older, 29% used a lower age bound between 30 to 40 years, and 17% between 20 to 29 years. Balancing these considerations with concern about attenuating national prevalence estimates by using too low a bound, we restricted our PD surveillance case definitions to persons aged 35 years and older.

Drug Codes

To ensure inclusion of all appropriate drugs when using pharmaceutical claims in the NNCSS case definitions, we examined drug use within the included literature, procured the Food and Drug Administration's (FDA's) list of approved drugs for Parkinson disease[172] (up to 2019, the anticipated timeframe of initial NNCSS estimates), and consulted with movement disorder specialists. The list of included drugs and their associated National Drug Codes (NDC) for 2019 analyses is found in eTable 10.

© 2026 Esper CD et al. *JAMA Network Open*.

eTable 10. Evaluation of Group A and E Case Definitions on Key Attributes of Public Health Surveillance Systems by Case Definition Group and Data Source Type – Part B: Other Surveillance System Attributes [a,b,c]

Source	Case Definition	*ICD* Code	Reference Standard PD or PD with OP[d]	Reference Standard Source	S	A	CE	R	S/S
**Group A**									
Data source type: claims									
Lee et al,[27] 2016[e]	• Inclusions: ≥2 inpatient or outpatient diagnosis (first and last diagnosis ≥90 d apart) and ≥3 PD pharmaceutical claims after index PD diagnosis (all in 3 y)<br>• Exclusions: secondary parkinsonism diagnosis (*ICD-9* code 332.1) at any time, or any neuroleptic pharmaceutical claim within 180 d prior to first PD diagnosis, or dementia diagnosis (*ICD-9* codes 290 or 331) prior to first PD diagnosis	*ICD-9* code 332.0	PD	PD cohort[f]	4	6	4	4	3
Liu et al,[28] 2016[e]	• Inclusions: ≥3 inpatient or outpatient diagnosis and ≥3 PD pharmaceutical claims after first diagnosis in 7 y and first and last inpatient/outpatient diagnosis separated by ≥90 d<br>• Exclusions: secondary parkinsonism diagnosis (ICD-9 code 332.1) at any time, or any neuroleptic pharmaceutical claim within 180 d prior to first PD diagnosis or ≥3 inpatient, or outpatient diagnosis for dementia (ICD-9 codes 290 or 331) prior to first PD diagnosis	*ICD-9* code 332.0	PD	PD cohort[f]	4	6	4	3	2
Szatmari et al,[35] 2019[e]	• Inclusions: ≥2 inpatient or outpatient diagnosis (1 diagnosis in ≥2 of 10 y)<br>• Exclusions: diagnosis with ICD-10 codes G21-G26 at any time	*ICD-10* code G20	PD	MRR[g]	5	4	6	4	3
Data source type: other administrative[h]									
Szumski et al,[36] 2009[e]	• Inclusions: ≥2 outpatient diagnosis (if most used diagnosis from highest specialist)	*ICD-9* code 332.0	PD	MRR[g]	4	6	3	3	1

© 2026 Esper CD et al. *JAMA Network Open*.

Source	Case Definition	*ICD* Code	Reference Standard PD or PD with OP[d]	Source	S	A	CE	R	S/S
	• Exclusions: any other code more diagnosed by the highest specialist on record or only 1 diagnosis of *ICD-9* code 332.0								
Szumski et al,[36] 2009[i]	• Inclusions: ≥2 outpatient diagnosis (if most used diagnosis from highest specialist) and ≥1 pharmaceutical claim<br>• Exclusions: any other code more diagnosed by the highest specialist on record or only 1 diagnosis of ICD-9 code 332.0	*ICD-9* code 332.0	PD	MRR[g]	3	6	3	3	1
**Group E**[j]									
Data source type: claims									
Noyes et al,[30] 2007[i]	Inclusions: ≥1 diagnosis (PHY only)	*ICD-9* codes 332.0, 332.1, 333.0, or 333.1	PD and OP	MCBS and pharmaceutical claims[k]	6	4	3	6	6
Noyes et al,[30] 2007[i]	Inclusions: ≥1 diagnosis (in any claims)	*ICD-9* codes 332.0, 332.1, 333.0, or 333.1	PD and OP	MCBS and pharmaceutical claims[k]	6	3	2	6	6
Noyes et al,[30] 2007[i]	Inclusions: ≥1 diagnosis (PHY only)	*ICD-9* codes 332.0, 332.1, 333.0, or 333.1	PD and OP	MCBS[l]	6	4	3	6	6
Noyes et al,[30] 2007[i]	Inclusions: ≥1 diagnosis (in any claims)	*ICD-9* codes 332.0, 332.1, 333.0, or 333.1	PD and OP	MCBS[l]	6	3	2	6	6
Data source type: other administrative									
Feldman et al,[23] 2012[i]	Inclusions: ≥1 inpatient diagnosis	*ICD-9* codes 332.0 or 333.0; *ICD-10* codes G20, G21.4, G21.8-G21.9, G23.1-G23.2, G23.9, or G25.9; or *ICD 7-8* codes	PD and OP	SALT study[m]	6	3	3	6	6
Swarztrauber et al,[33] 2005[i]	Inclusions: ≥1 inpatient or outpatient diagnosis	*ICD-9* codes 332.0, 332.1, or 333.0	PD and OP	MRR[g]	6	4	3	6	6

© 2026 Esper CD et al. *JAMA Network Open*.

Source	Case Definition	*ICD* Code	Reference Standard PD or PD with OP[d]	Source	S	A	CE	R	S/S
Swarztrauber et al,[33] 2005[i]	Inclusions: (≥1 inpatient or outpatient diagnosis) or (≥1 pharmaceutical claim)	*ICD-9* codes 332.0, 332.1, 333.0, or 781.0	PD and OP	MRR[g]	6	2	2	6	6
Swarztrauber et al,[33] 2005[i]	Inclusions: 1 pharmaceutical claim	NU	PD and OP	MRR[g]	6	3	2	6	6
Swarztrauber et al,[33] 2005[e]	Inclusions: (≥1 inpatient or outpatient diagnosis) or (≥1 pharmaceutical claim)	*ICD-9* codes 332.0, 332.1, or 333.0	PD and OP	MRR[g]	6	3	2	6	6
Szumski et al,[36] 2009[e]	Inclusions: 1 pharmaceutical claim	NU	PD	MRR[g]	6	3	2	6	6
Data source type: claims and other administrative									
Baldacci et al,[20] 2015[e]	Inclusions: ≥1 of the following: ≥1 inpatient PD diagnosis, PD exemption, or ≥2 PD pharmaceutical claim dispensed in 1 y ≥6 mo apart	*ICD-9* code 332	PD	PD cohort[f]	5	3	2	2	2
Butt et al,[21] 2014[i,n]	Inclusions: 1 pharmaceutical claim	NU	PD and OP	MRR[g]	6	3	2	6	6
Butt et al,[21] 2014[e,n,o]	Inclusions: (≥2 outpatient diagnosis ≥30 d apart in 1 y) or (1 pharmaceutical claim and 1 outpatient diagnosis 6 mo before or after pharmaceutical claim)	*ICD-9* codes 332, 332.0, or 332.1; or *ICD-10* codes G20, G21.0-G21.4, G21.8-G21.9, G22, or F02.3	PD and OP	MRR[g]	5	6	6	5	6
Butt et al,[21] 2014[e,n,o]	Inclusions: 2 outpatient diagnosis ≥30 d apart in 1 y	*ICD-9* codes 332, 332.0, or 332.1; or *ICD-10* codes G20, G21.0-G21.4, G21.8-G21.9, G22, or F02.3	PD and OP	MRR[g]	5	4	5	6	6
Butt et al,[21] 2014[i,n]	Inclusions: 1 outpatient diagnosis or 1 pharmaceutical claim	*ICD-9* codes 332, 332.0, or 332.1; or *ICD-10* codes G20, G21.0-G21.4, G21.8-G21.9, G22,	PD and OP	MRR[g]	6	3	2	6	6

© 2026 Esper CD et al. *JAMA Network Open*.

Source	Case Definition	*ICD* Code	Reference Standard PD or PD with OP[d]	Source	S	A	CE	R	S/S
		or F02.3							
Butt et al,[21] 2014[i,n]	Inclusions: 1 outpatient diagnosis and 1 pharmaceutical claim	*ICD-9* codes 332, 332.0, or 332.1; or *ICD-10* codes G20, G21.0-G21.4, G21.8-G21.9, G22, or F02.3	PD and OP	MRR[g]	6	4	4	6	6
Butt et al,[21] 2014[i,n]	Inclusions: 1 pharmaceutical claim and 1 outpatient diagnosis 6 mo before or after pharmaceutical claim	*ICD-9* codes 332, 332.0, or 332.1; or *ICD-10* codes G20, G21.0-G21.4, G21.8-G21.9, G22, or F02.3	PD and OP	MRR[g]	5	4	5	6	6
Butt et al,[21] 2014[i,n]	Inclusions: 1 outpatient diagnosis and 1 pharmaceutical claim, all in 1 y	*ICD-9* codes 332, 332.0, or 332.1; or *ICD-10* codes G20, G21.0-G21.4, G21.8-G21.9, G22, or F02.3	PD and OP	MRR[g]	5	4	5	6	6
Butt et al,[21] 2014[i,n]	Inclusions: 2 outpatient diagnosis ≥30 d apart and 1 pharmaceutical claim, all in 1 y	*ICD-9* codes 332, 332.0, or 332.1; or *ICD-10* codes G20, G21.0-G21.4, G21.8-G21.9, G22, or F02.3	PD and OP	MRR[g]	5	5	5	6	6
Butt et al,[21] 2014[i,n]	Inclusions: (≥2 outpatient diagnosis ≥30 d apart in 1 y) or (1 pharmaceutical claim and 1 outpatient diagnosis)	*ICD-9* codes 332, 332.0, or 332.1; or *ICD-10* codes G20, G21.0-G21.4, G21.8-G21.9, G22, or F02.3	PD and OP	MRR[g]	5	5	5	6	6
Butt et al,[21] 2014[i,n]	Inclusions: 1 pharmaceutical claim and 1 inpatient or outpatient diagnosis 6 mo before or after pharmaceutical claim	*ICD-9* codes 332, 332.0, or 332.1; or *ICD-10* codes G20, G21.0-G21.4, G21.8-G21.9, G22, or F02.3	PD and OP	MRR[g]	5	4	5	6	6

© 2026 Esper CD et al. *JAMA Network Open*.

Source	Case Definition	*ICD* Code	Reference Standard PD or PD with OP[d]	Source	S	A	CE	R	S/S
Butt et al,[21] 2014[i,n]	Inclusions: (1 inpatient diagnosis or 2 outpatient diagnosis ≥30 d apart in 1 y) or (1 pharmaceutical claim and 1 inpatient or outpatient diagnosis)	*ICD-9* codes 332, 332.0, or 332.1; or *ICD-10* codes G20, G21.0-G21.4, G21.8-G21.9, G22, or F02.3	PD and OP	MRR[g]	5	4	5	5	5
Butt et al,[21] 2014[i,n]	Inclusions: (1 inpatient diagnosis or 2 outpatient diagnosis ≥30 d apart in 1 y) or (1 pharmaceutical claim and 1 inpatient or outpatient diagnosis 6 mo before or after pharmaceutical claim)	*ICD-9* codes 332, 332.0, or 332.1; or *ICD-10* codes G20, G21.0-G21.4, G21.8-G21.9, G22, or F02.3	PD and OP	MRR[g]	4	4	5	5	5
Butt et al,[21] 2014[i,n]	Inclusions: 1 inpatient diagnosis	*ICD-9* codes 332, 332.0, or 332.1; or *ICD-10* codes G20, G21.0-G21.4, G21.8-G21.9, G22, or F02.3	PD and OP	MRR[g]	6	3	3	6	6
Butt et al,[21] 2014[i,n]	Inclusions: 1 inpatient or emergency department or same-day surgery diagnosis	*ICD-9* codes 332, 332.0, or 332.1; or *ICD-10* codes G20, G21.0-G21.4, G21.8-G21.9, G22, or F02.3	PD and OP	MRR[g]	5	2	3	5	5
Butt et al,[21] 2014[i,n]	Inclusions: 1 outpatient diagnosis	*ICD-9* codes 332, 332.0, or 332.1; or *ICD-10* codes G20, G21.0-G21.4, G21.8-G21.9, G22, or F02.3	PD and OP	MRR[g]	6	4	4	6	6
Butt et al,[21] 2014[i,n]	Inclusions: 1 outpatient diagnosis (specialist only)	*ICD-9* codes 332, 332.0, or 332.1; or *ICD-10* codes G20, G21.0-G21.4, G21.8-G21.9, G22, or F02.3	PD and OP	MRR[g]	4	5	4	4	4
Butt et al,[21]	Inclusions: 2 outpatient diagnosis in 1 y	*ICD-9* codes 332, 332.0, or 332.1; or	PD and OP	MRR[g]	6	5	5	6	6

© 2026 Esper CD et al. *JAMA Network Open*.

Source	Case Definition	*ICD* Code	Reference Standard PD or PD with OP[d]	Source	S	A	CE	R	S/S
2014[i,n]		*ICD-10* codes G20, G21.0-G21.4, G21.8-G21.9, G22, or F02.3							
Butt et al,[21] 2014[i,n]	Inclusions: 2 outpatient diagnosis ≥30 d apart in 1 y (specialist only)	*ICD-9* codes 332, 332.0, or 332.1; or *ICD-10* codes G20, G21.0-G21.4, G21.8-G21.9, G22, or F02.3	PD and OP	MRR[g]	3	6	4	3	3
Butt et al,[21] 2014[i,n]	Inclusions: 2 outpatient diagnosis ≥30 d apart in 2 y	*ICD-9* codes 332, 332.0, or 332.1; or *ICD-10* codes G20, G21.0-G21.4, G21.8-G21.9, G22, or F02.3	PD and OP	MRR[g]	5	4	4	6	6
Butt et al,[21] 2014[i,n]	Inclusions: 2 outpatient diagnosis ≥30 d apart in 3 y	*ICD-9* codes 332, 332.0, or 332.1; or *ICD-10* codes G20, G21.0-G21.4, G21.8-G21.9, G22, or F02.3	PD and OP	MRR[g]	5	4	3	5	5
Butt et al,[21] 2014[i,n]	Inclusions: 3 outpatient diagnosis ≥30 d apart in 1 y	*ICD-9* codes 332, 332.0, or 332.1; or *ICD-10* codes G20, G21.0-G21.4, G21.8-G21.9, G22, or F02.3	PD and OP	MRR[g]	5	5	5	6	6
Butt et al,[21] 2014[i,n]	Inclusions: (1 inpatient diagnosis) or (2 outpatient diagnosis ≥30 d apart, all in 1 y)	*ICD-9* codes 332, 332.0, or 332.1; or *ICD-10* codes G20, G21.0-G21.4, G21.8-G21.9, G22, or F02.3	PD and OP	MRR[g]	5	4	4	6	6
Butt et al,[21] 2014[e,o,p]	Inclusions: 2 outpatient diagnosis ≥30 d apart in 1 y	*ICD-9* codes 332, 332.0, or 332.1; or *ICD-10* codes G20, G21.0-G21.4,	PD and OP	MRR[g]	5	4	5	6	6

© 2026 Esper CD et al. *JAMA Network Open*.

Source	Case Definition	*ICD* Code	Reference Standard PD or PD with OP[d]	Source	S	A	CE	R	S/S
		G21.8-G21.9, G22, or F02.3							
Butt et al,[21] 2014[e,o,p]	Inclusions: 1 pharmaceutical claim and 1 outpatient diagnosis 6 mo before or after pharmaceutical claim	*ICD-9* codes 332, 332.0, or 332.1; or *ICD-10* codes G20, G21.0-G21.4, G21.8-G21.9, G22, or F02.3	PD and OP	MRR[g]	5	4	5	6	6
Butt et al,[21] 2014[i,p]	Inclusions: 1 inpatient diagnosis	*ICD-9* codes 332, 332.0, or 332.1; or *ICD-10* codes G20, G21.0-G21.4, G21.8-G21.9, G22, or F02.3	PD and OP	MRR[g]	6	3	3	6	6
Butt et al,[21] 2014[i,p]	Inclusions: 1 inpatient or emergency department or same-day surgery diagnosis	*ICD-9* codes 332, 332.0, or 332.1; or *ICD-10* codes G20, G21.0-G21.4, G21.8-G21.9, G22, or F02.3	PD and OP	MRR[g]	5	2	3	5	5
Butt et al,[21] 2014[i,p]	Inclusions: 1 outpatient diagnosis	*ICD-9* codes 332, 332.0, or 332.1; or *ICD-10* codes G20, G21.0-G21.4, G21.8-G21.9, G22, or F02.3	PD and OP	MRR[g]	6	4	4	6	6
Butt et al,[21] 2014[i,p]	Inclusions: 1 outpatient diagnosis (specialist only)	*ICD-9* codes 332, 332.0, or 332.1; or *ICD-10* codes G20, G21.0-G21.4, G21.8-G21.9, G22, or F02.3	PD and OP	MRR[g]	4	5	4	4	4
Butt et al,[21] 2014[i,p]	Inclusions: 2 outpatient diagnosis in 1 y	*ICD-9* codes 332, 332.0, or 332.1; or *ICD-10* codes G20, G21.0-G21.4, G21.8-G21.9, G22, or F02.3	PD and OP	MRR[g]	6	5	5	6	6

© 2026 Esper CD et al. *JAMA Network Open*.

Source	Case Definition	*ICD* Code	Reference Standard PD or PD with OP[d]	Source	S	A	CE	R	S/S
Butt et al,[21] 2014[i,p]	Inclusions: 2 outpatient diagnosis ≥30 d apart in 1 y (specialist only)	*ICD-9* codes 332, 332.0, or 332.1; or *ICD-10* codes G20, G21.0-G21.4, G21.8-G21.9, G22, or F02.3	PD and OP	MRR[g]	3	6	4	3	3
Butt et al,[21] 2014[i,p]	Inclusions: 2 outpatient diagnosis ≥30 d apart in 2 y	*ICD-9* codes 332, 332.0, or 332.1; or *ICD-10* codes G20, G21.0-G21.4, G21.8-G21.9, G22, or F02.3	PD and OP	MRR[g]	5	4	4	6	6
Butt et al,[21] 2014[i,p]	Inclusions: 2 outpatient diagnosis ≥30 d apart in 3 y	*ICD-9* codes 332, 332.0, or 332.1; or *ICD-10* codes G20, G21.0-G21.4, G21.8-G21.9, G22, or F02.3	PD and OP	MRR[g]	5	4	3	5	5
Butt et al,[21] 2014[i,p]	Inclusions: 3 outpatient diagnosis ≥30 d apart in 1 y	*ICD-9* codes 332, 332.0, or 332.1; or *ICD-10* codes G20, G21.0-G21.4, G21.8-G21.9, G22, or F02.3	PD and OP	MRR[g]	5	5	5	6	6
Butt et al,[21] 2014[i,p]	Inclusions: 1 inpatient diagnosis or 2 diagnosis ≥30 d apart, all in 1 y	*ICD-9* codes 332, 332.0, or 332.1; or *ICD-10* codes G20, G21.0-G21.4, G21.8-G21.9, G22, or F02.3	PD and OP	MRR[g]	5	4	4	6	6
Butt et al,[21] 2014[i,p]	Inclusions: 1 outpatient diagnosis or 1 pharmaceutical claim	*ICD-9* codes 332, 332.0, or 332.1; or *ICD-10* codes G20, G21.0-G21.4, G21.8-G21.9, G22, or F02.3	PD and OP	MRR[g]	6	3	2	6	6
Butt et al,[21] 2014[i,p]	Inclusions: 1 outpatient diagnosis and 1 pharmaceutical claim	*ICD-9* codes 332, 332.0, or 332.1; or	PD and OP	MRR[g]	6	3	2	6	6

© 2026 Esper CD et al. *JAMA Network Open*.

Source	Case Definition	*ICD* Code	Reference Standard PD or PD with OP[d]	Source	S	A	CE	R	S/S
		ICD-10 codes G20, G21.0-G21.4, G21.8-G21.9, G22, or F02.3							
Butt et al,[21] 2014[i,p]	Inclusions: 1 pharmaceutical claim	*ICD-9* codes 332, 332.0, or 332.1; or *ICD-10* codes G20, G21.0-G21.4, G21.8-G21.9, G22, or F02.3	PD and OP	MRR[g]	6	4	4	6	6
Butt et al,[21] 2014[i,p]	Inclusions: 1 outpatient diagnosis and 1 pharmaceutical claim, all in 1 y	*ICD-9* codes 332, 332.0, or 332.1; or *ICD-10* codes G20, G21.0-G21.4, G21.8-G21.9, G22, or F02.3	PD and OP	MRR[g]	5	4	5	6	6
Butt et al,[21] 2014[i,p]	Inclusions: 2 outpatient diagnosis ≥30 d apart and 1 pharmaceutical claim, all in 1 y	*ICD-9* codes 332, 332.0, or 332.1; or *ICD-10* codes G20, G21.0-G21.4, G21.8-G21.9, G22, or F02.3	PD and OP	MRR[g]	5	5	5	6	6
Butt et al,[21] 2014[i,p]	Inclusions: (≥2 outpatient diagnosis ≥30 d apart in 1 y) or (1 pharmaceutical claim and 1 outpatient diagnosis)	*ICD-9* codes 332, 332.0, or 332.1; or *ICD-10* codes G20, G21.0-G21.4, G21.8-G21.9, G22, or F02.3	PD and OP	MRR[g]	5	5	5	6	6
Butt et al,[21] 2014[i,p]	Inclusions: (≥2 outpatient diagnosis ≥30 d apart in 1 y) or (1 pharmaceutical claim and 1 outpatient diagnosis 6 mo before or after pharmaceutical claim)	*ICD-9* codes 332, 332.0, or 332.1; or *ICD-10* codes G20, G21.0-G21.4, G21.8-G21.9, G22, or F02.3	PD and OP	MRR[g]	5	6	6	5	5
Butt et al,[21] 2014[i,p]	Inclusions: 1 pharmaceutical claim and 1 inpatient or outpatient diagnosis 6 mo before or after pharmaceutical claim	*ICD-9* codes 332, 332.0, or 332.1; or *ICD-10* codes G20, G21.0-G21.4,	PD and OP	MRR[g]	5	4	5	6	6

© 2026 Esper CD et al. *JAMA Network Open*.

Source	Case Definition	*ICD* Code	Reference Standard PD or PD with OP[d]	Source	S	A	CE	R	S/S
		G21.8-G21.9, G22, or F02.3							
Butt et al,[21] 2014[i,p]	Inclusions: (1 inpatient diagnosis or ≥2 outpatient diagnosis ≥30 d apart in 1 y) or (1 pharmaceutical claim and 1 inpatient or outpatient diagnosis)	*ICD-9* codes 332, 332.0, or 332.1; or *ICD-10* codes G20, G21.0-G21.4, G21.8-G21.9, G22, or F02.3	PD and OP	MRR[g]	4	4	5	5	5
Butt et al,[21] 2014[i,p]	Inclusions: (1 inpatient diagnosis or 2 outpatient diagnosis ≥30 d apart in 1 y) or (1 pharmaceutical claim and 1 inpatient or outpatient diagnosis 6 mo before or after pharmaceutical claim)	*ICD-9* codes 332, 332.0, or 332.1; or *ICD-10* codes G20, G21.0-G21.4, G21.8-G21.9, G22, or F02.3	PD and OP	MRR[g]	4	4	5	5	5
Data source type: EHR and linked claims									
Wei et al,[38] 2016[i]	Inclusions: ≥1 pharmaceutical claim	NU	PD	MRR[g]	6	3	2	6	6
Wei et al,[38] 2016[i]	Inclusions: ≥1 mention of PD in EHR	NU	PD	MRR[g]	6	2	1	1	3
Data source type: survey									
Jain et al,[26] 2015[i]	Inclusions: ≥1 PD pharmaceutical claim (self-reported)	NU	PD	Framingham Heart Study[q]	5	2	2	4	4
Jain et al,[26] 2015[i]	Inclusions: yes to ≥1 of the following (self-reported): diagnosed with PD, hospitalized with PD, or ≥1 PD pharmaceutical claim	NU	PD	Framingham Heart Study[q]	5	2	2	4	4
Jain et al,[26] 2015[e]	Inclusions: yes to ≥1 of the following (self-reported): diagnosed with PD or hospitalized with PD	NU	PD	Framingham Heart Study[q]	6	3	2	4	4

Abbreviations: A, acceptability; CE, cost-effectiveness; EHR, electronic health records; *ICD*, *International Classification of Diseases*; MCBS, Medicare Current Beneficiary Survey; MRR, medical record review; NPV, negative predictive value; NR, not reported; NU, not used; OP, other parkinsonisms; PD, Parkinson disease; PHY, physician or carrier claims; PPV, positive predictive value; R, reproducibility; S, simplicity; S/S, scalability and spreadability; SALT, Screening Across the Lifespan Twin study.

[a]Table 2 provides more information on case definition groups. Table 3 includes information on each case definition's measures of accuracy.

[b]Data source type refers to the type of data source to which the case definition was applied.

[c]Ratings based on National Neurological Conditions Surveillance System's perceived assessment of the key attributes of surveillance systems[18] listed for each case definition relative to the other case definitions assessed. Case definitions were rated 1 to 6, with 1 being the lowest performance and 6 being the highest.

[d]PD with OP refers to a reference standard that compares the case definition to a gold standard for PD and OP.

[e]Case definition selected by authors of the cited study after they validated case definition(s) in their study.

[f]PD cohort refers to a group of people previously diagnosed with PD.

© 2026 Esper CD et al. *JAMA Network Open*.

[g]MRR was referred to in the cited articles as “medical chart review.”
[h]For the purposes of this study, administrative data–based studies were split into 2 categories: claims and other. Other administrative data source types included medical or billing information that is not claims based (eg, aggregated information pulled from the EHR for research or administrative purposes).
[i]Case definition not selected by authors of the cited study after they validated case definition(s) in their study.
[j]Group E case definitions did not include any exclusions.
[k]Noyes et al[30] reference standard using MCBS self-report of PD or self-reported use of PD medication.
[l]Noyes et al[30] reference standard using MCBS self-report of PD.
[m]Feldman et al[23] reference standard was SALT, a longitudinal study that included diagnoses of PD based on survey and specialist medical record review.
[n]Butt et al[21] case definition validated in a cohort of persons aged 20 years and older.
[o]Butt et al[21] selected 4 case definitions: (1) age 20 years and older with pharmaceutical claims, (2) age 20 years and older without pharmaceutical claims, (3) age 65 years and older with pharmaceutical claims, and (4) age 65 years and older without pharmaceutical claims.
[p]Butt et al,[21] 2014 case definition validated in a cohort of persons aged 65 years and older.
[q]Jain et al,[26] 2015 reference standard using Framingham Heart Study, a longitudinal study that included diagnosis of PD based on neurologist examination and MRR.

© 2026 Esper CD et al. *JAMA Network Open*.

eTable 11. NNCSS's Selected Parkinson Disease Surveillance Case Definitions and Characteristics

	PD Probable case definition	PD Possible case definition
**Case definition**	≥2 IP or OP Dx of PD[a] ≥90 d apart<br>OR<br>≥1 IP or OP Dx of PD ≥90 d followed by ≥2 PD Rx[b]<br><br>Exclusion applied to all possible cases:<br>≥1 IP or OP Dx for any other parkinsonism[c] at any time	≥2 OP Dx of PD[a] or other parkinsonism[c] ≥30 d apart, in 1 y<br>OR<br>≥1 PD Rx[b] + ≥1 OP Dx of PD or other parkinsonism 6 mo before or after PD Rx
**Characteristics**	• More specific<br>• Includes people with PD and unlikely to include people with other parkinsonisms<br>• May miss some people with uncertain or unclear PD diagnoses	• More sensitive<br>• Includes people with PD, people with incomplete or unclear evidence of PD, and people with other parkinsonisms whose physical features may overlap with PD<br>• Includes people with PD and a portion of people with some other parkinsonisms, but is not a case definition for PD and all other parkinsonisms

Abbreviations: Dx, diagnostic claim; IP, inpatient; NNCSS, National Neurological Conditions Surveillance System; OP, outpatient; PD, Parkinson disease; Rx, pharmaceutical claim.
[a] Dx of PD: ICD-10 Code G20 (See eTable 9).
[b] All included medications are US Food and Drug Administration-approved for PD (See eTable 11).
[c] Dx of other parkinsonisms: ICD-10 codes G21.X, G23.X, G31.83, G31.85, G90.3 (See eTable 9).

© 2026 Esper CD et al. *JAMA Network Open*.

eTable 12. Medications Used in NNCSS's Parkinson Disease Case Definitions[a,b]

Drug	Product National Drug Code(s)
Lodosyn (carbidopa)	25010-711
Generic carbidopa	16714-067, 40032-980, 42799-123, 43386-980, 43975-220, 47781-332, 51407-314, 59651-146, 63629-1932, 68682-200, 70710-1221, 70771-1355
Dhivy (carbidopa/levodopa)	75854-701
Duopa (carbidopa/levodopa)	0074-3012
Rytary (carbidopa/levodopa extended release)	64896-661, 64896-662, 64896-663, 64896-664
Sinemet (carbidopa/levodopa)	78206-166, 78206-167, 78206-168
Generic carbidopa/levodopa (including extended release and controlled release)	0093-9701, 0093-9702, 0228-2538, 0228-2539, 0228-2540, 0378-0078, 0378-0085, 0378-0088, 0378-0094, 0378-1133, 0615-8067, 0615-8180, 0615-8181, 0615-8251, 0615-8275, 0904-6237, 0904-6238, 16729-078, 16729-079, 42291-465, 42291-466, 42291-467, 42291-472, 43353-202, 43353-327, 46708-332, 46708-333, 47335-186, 47335-187, 47335-188, 50090-3419, 50090-3857, 50228-457, 50228-458, 50228-459, 50228-460, 50228-461, 51079-923, 51079-978, 51407-166, 51407-167, 51407-168, 51862-078, 51862-079, 51862-080, 51862-855, 51862-856, 51862-858, 55154-7886, 62332-332, 62332-333, 62756-457, 62756-461, 62756-517, 62756-518, 62756-519, 62756-985, 63629-2355, 63629-2356, 63629-2357, 63629-2358, 63629-9154, 63739-108, 67544-672, 68001-171, 68001-172, 68071-2240, 68084-093, 68084-094, 68084-281, 68084-282, 69367-338, 69367-339, 69367-340, 70518-2908, 70518-2909, 70518-3250, 71205-877, 71205-878, 71205-879, 71610-269, 71610-579
Inbrija (levodopa)	10144-342
Stalevo (carbidopa/levodopa/entacapone)	0078-0407, 0078-0409, 0078-0527, 52427-805, 52427-809, 52427-816, 52427-827, 52427-834, 52427-842
Generic carbidopa/levodopa/entacapone	0378-8302, 0378-8305, 0781-5613, 0781-5625, 0781-5637, 0781-5641, 0781-5654, 0781-5669, 16571-689, 16571-690, 16571-691, 16571-692, 16571-693, 16571-694, 64679-782, 64679-783, 64679-784, 64679-785, 64679-786, 64679-787
Comtan (entacapone)	0078-0327, 52427-800
Generic entacapone	0378-9080, 0527-1830, 0615-8298, 0781-5578, 0904-6822, 27241-049, 33342-260, 43353-996, 46708-478, 47335-007, 50268-295, 51079-273, 60687-188, 62332-478, 64679-711, 64679-781, 65862-654, 71610-247
Tasmar (tolcapone)	0187-0938
Generic tolcapone	50742-193, 68682-938
Ongentys (opicapone)	70370-3025, 70370-3050
Mirapex (pramipexole dihydrochloride)	0597-0109, 0597-0113, 0597-0115, 0597-0116, 0597-0285, 0597-0286, 0597-0287
Generic pramipexole dihydrochloride (including extended release)	0904-6704, 13668-091, 13668-092, 13668-093, 13668-094, 13668-095, 13668-184, 16714-916, 16714-917, 16714-918, 16714-919, 16714-920, 16714-921, 16714-922, 29300-207, 29300-208, 29300-209, 29300-210, 29300-211, 29300-270, 33342-208, 33342-209, 33342-210, 33342-211, 33342-212, 33342-213, 33342-214, 46708-003, 46708-004, 46708-005,

© 2026 Esper CD et al. *JAMA Network Open*.

Drug	Product National Drug Code(s)
	46708-006, 46708-007, 46708-574, 46708-575, 46708-576, 46708-577, 46708-578, 46708-579, 46708-580, 46708-611, 46708-612, 46708-613, 46708-614, 46708-615, 50090-2190, 50090-2217, 50090-2455, 50090-3280, 50090-3350, 50090-5036, 50228-126, 50228-127, 50228-128, 50228-129, 50228-130, 50228-131, 50742-331, 50742-332, 50742-333, 50742-334, 50742-335, 50742-336, 50742-337, 55111-611, 55111-612, 55111-613, 55111-614, 55111-615, 57237-180, 57237-181, 57237-182, 57237-183, 57237-184, 57237-185, 60429-085, 60429-086, 60429-087, 60429-089, 60429-090, 60687-570, 60687-581, 60687-592, 62332-003, 62332-004, 62332-005, 62332-006, 62332-007, 62332-154, 62332-155, 62332-156, 62332-157, 62332-158, 62332-159, 62332-160, 63629-5013, 63629-5034, 63629-5042, 63629-8289, 64380-746, 64380-747, 64380-748, 64380-749, 64380-750, 64380-751, 65841-734, 65841-735, 65841-736, 65841-737, 65841-738, 65862-604, 65862-605, 65862-606, 65862-607, 65862-608, 65862-609, 68382-196, 68382-197, 68382-198, 68382-199, 68382-200, 68382-474, 68382-475, 68382-476, 68382-477, 68382-478, 68382-874, 68382-875, 68462-330, 68462-331, 68462-332, 68462-333, 68462-334, 68462-627, 69680-145, 69680-146, 70771-1328, 70771-1329, 70771-1330, 70771-1331, 70771-1332, 70771-1333, 70771-1334, 71034-002, 71034-003, 71335-0584, 71335-1504, 71335-1537, 71335-1808, 71335-1877
Generic ropinirole (including extended release)	0228-3640, 0228-3658, 0228-3659, 0228-3660, 0228-3661, 0378-5501, 0378-5502, 0378-5503, 0378-5504, 0378-5505, 0378-5525, 0378-5550, 0615-8188, 0615-8189, 0615-8190, 0615-8191, 0781-5780, 0781-5782, 0781-5784, 0781-5786, 0781-5788, 0904-6373, 0904-6374, 0904-6375, 0904-6376, 0904-6377, 0904-6378, 0904-6379, 16729-232, 16729-233, 16729-234, 16729-235, 16729-236, 16729-237, 16729-238, 43353-258, 43353-265, 43353-266, 43353-267, 43547-268, 43547-269, 43547-270, 43547-271, 43547-272, 43547-273, 43547-274, 43547-595, 43547-596, 43547-597, 43547-598, 43547-599, 43547-600, 43547-601, 46708-030, 46708-031, 46708-032, 46708-033, 46708-034, 46708-035, 46708-036, 46708-262, 46708-263, 46708-264, 46708-265, 46708-266, 50090-2068, 50090-3304, 50090-4348, 50090-4684, 50090-4803, 50090-4810, 50090-5167, 51655-360, 55111-659, 55111-661, 55111-662, 55111-727, 55111-728, 55154-7633, 551, 54-7888, 60429-817, 60429-818, 60429-819, 60429-820, 60429-821, 60429-822, 60429-823, 60687-577, 60687-588, 61919-564, 61919-780, 62332-030, 62332-031, 62332-032, 62332-033, 62332-034, 62332-035, 62332-036, 62332-107, 62332-108, 62332-109, 62332-110, 62332-111, 63629-4798, 63629-7152, 63629-8123, 65841-712, 65841-713, 65841-714, 65841-715, 65841-716, 65841-717, 65841-718, 68462-253, 68462-254, 68462-255, 68462-256, 68462-257, 68462-258, 68462-259, 70518-2439, 70518-2476, 70518-2450, 71335-0064, 71335-0587, 71335-0727, 71335-0829, 71335-1167, 71335-1188, 71335-1231, 71335-1434, 71335-1626, 71335-1982, 72189-222

© 2026 Esper CD et al. *JAMA Network Open*.

Drug	Product National Drug Code(s)
Neupro (rotigotine)	50474-801, 50474-802, 50474-803, 50474-804, 50474-805, 50474-806, 50474-808
Gocovri (amantadine hydrochloride)	70482-085, 70482-170
Osmolex extended release (amantadine hydrochloride)	68025-074, 68025-075, 68025-076, 70482-075, 70482-076
Generic amantadine hydrochloride	0121-0646, 0527-1704, 0591-4920, 0615-8263, 0832-0111, 0832-1015, 0904-6630, 0904-7042, 10135-692, 10135-693, 17856-0646, 23155-362, 24689-105, 24689-112, 42291-125, 42543-493, 42543-497, 46708-246, 46708-586, 50090-0020, 50268-069, 50383-807, 53002-3750, 55154-9444, 59746-699, 60432-093, 60687-239, 60687-422, 62332-246, 62332-586, 65841-835, 68382-512, 69097-925, 69097-926, 69452-142, 70518-3381, 71205-223, 71335-0279, 71335-0469, 72888-033, 73152-075, 73152-076
Apokyn (apomorphine hydrochloride)	27505-004
Kynmobi (apomorphine hydrochloride)	63402-010, 63402-015, 63402-020, 63402-025, 63402-030, 63402-088, 63402-110, 63402-115, 63402-120, 63402-125, 63402-130, 63402-188
Generic apomorphine hydrochloride	52817-720
Nourianz (istradefylline)	42747-602, 42747-604
Azilect (rasagiline mesylate)	68546-142, 68546-229
Generic rasagiline mesylate	0378-1270, 0378-1271, 23155-746, 23155-747
Xadago (safinamide mesylate)	27505-110, 27505-111
Zelapar (selegiline hydrochloride)	0187-0453
Generic selegiline hydrochloride	16571-659, 50090-2918, 60429-176, 60505-0055, 60505-3438, 70954-504, 71205-888, 72319-006

Abbreviations: NNCSS, National Neurological Conditions Surveillance System; PD, Parkinson Disease.
[a] All included drugs are US Food and Drug Administration approved for PD treatment.
[b] List of drugs current to 2022 as NNCSS's first PD analyses used data from 2017-2019. This list will be updated as needed for future analyses using newer data.

eReferences

1. Whiting PF, Rutjes AWS, Westwood ME, et al. QUADAS-2: A Revised Tool for the Quality Assessment of Diagnostic Accuracy Studies. *Annals of Internal Medicine*. 2011/10/18 2011;155:529-536. doi:10.7326/0003-4819-155-8-201110180-00009
2. Shamliyan TA, Kane RL, Ansari MT, et al. Development quality criteria to evaluate nontherapeutic studies of incidence, prevalence, or risk factors of chronic diseases: pilot study of new checklists. *Journal of Clinical Epidemiology*. 2011/06/01/ 2011;64(6):637-657. doi:10.1016/j.jclinepi.2010.08.006
3. Groseclose SL, Buckeridge DL. Public Health Surveillance Systems: Recent Advances in Their Use and Evaluation. *Annu Rev Public Health*. Mar 2017;38:57-79. doi:10.1146/annurev-publhealth-031816-044348
4. El Burai Felix S, Yusuf H, Ritchey M, et al. A Standard Framework for Evaluating Large Health Care Data and Related Resources. *Morbidity and Mortality Weekly Report Supplement*. 2024;(73)(3):1-13. doi:10.15585/mmwr.su7303a1
5. Aamodt WW, Travers J, Thibault D, Willis AW. Hospital Magnet Status Associates With Inpatient Safety in Parkinson Disease. *Journal of Neuroscience Nursing*. Jun 1 2021;53(3):116-122. doi:10.1097/JNN.0000000000000582
6. Aamodt WW, Dahodwala N, Bilker WB, Farrar JT, Willis AW. Unique characteristics of end-of-life hospitalizations in Parkinson disease. *Front Aging Neurosci*. Oct 11 2023;15doi:10.3389/fnagi.2023.1254969
7. Abuhasira R, Zlotnik Y, Horev A, Ifergane G. Fibromyalgia-Like Syndrome Associated with Parkinson's Disease-A Cohort Study. *J Clin Med*. Jul 28 2019;8(8):1118. doi:10.3390/jcm8081118
8. Albarmawi H, Zhou S, Shumlman LM, et al. The economic burden of Parkinson disease among Medicare beneficiaries. *Journal of Managed Care & Speciality Pharmacy*. 2022;28(4):405-414. doi:10.18553/jmcp.2022.28.4.405
9. Alonso A, Rodriguez LA, Logroscino G, Hernán MA. Use of antidepressants and the risk of Parkinson's disease: a prospective study. *J Neurol Neurosurg Psychiatry*. Jun 2009;80(6):671-674. doi:10.1136/jnnp.2008.152983
10. Baldacci F, Policardo L, Rossi S, et al. Reliability of administrative data for the identification of Parkinson's disease cohorts. Research Support, Non-U.S. Gov't. *Neurological Sciences*. Feb 08 2015;36(5):783-786. doi:10.1007/s10072-015-2062-z
11. Barer Y, Gurevich T, Chodick G, et al. Advanced Stage Parkinson's Disease: From Identification to Characterization Using a Nationwide Database. *Movement Disorders Clinical Practice*. 2022;9(4):458-467. doi:10.1002/mdc3.13458
12. Becker C, Jick SS, Meier CR. Use of statins and the risk of Parkinson's disease: a retrospective case-control study in the UK. *Drug Safety*. 2008;31:399-407.
13. Becker C, Brobert GP, Johansson S, Jick SS, Meier CR. Risk of incident depression in patients with Parkinson disease in the UK. *Eur J Neurol*. Mar 2011;18(3):448-453. doi:10.1111/j.1468-1331.2010.03176.x
14. Bhattacharjee S, Sambamoorthi U. Diabetes care among elderly medicare beneficiaries with Parkinson's disease and diabetes. *J Diabetes Metab Disord*. 2015;14:75. doi:10.1186/s40200-015-0209-3
15. Bhattacharjee S, Vadiei N, Goldstone L, Alrabiah Z, Sherman SJ. Patterns and Predictors of Depression Treatment among Older Adults with Parkinson's Disease and Depression in Ambulatory Care Settings in the United States. *Parkinsons Dis*. 2018;2018:3402983. doi:10.1155/2018/3402983
16. Blin P, Dureau-Pournin C, Foubert-Samier A, et al. Parkinson's disease incidence and prevalence assessment in France using the national healthcare insurance database. Research Support, Non-U.S. Gov't. *Eur J Neurol*. Mar 2015;22(3):464-471. doi:10.1111/ene.12592
17. Brakedal B, Toker L, Haugarvoll K, Tzoulis C. A nationwide study of the incidence, prevalence and mortality of Parkinson's disease in the Norwegian population. *NPJ Parkinsons Dis*. Mar 2 2022;8(1):19. doi:10.1038/s41531-022-00280-4
18. Brandt-Christensen M, Kvist K, Nilsson FM, Andersen PK, Kessing LV. Use of antiparkinsonian drugs in Denmark: Results from a nationwide pharmacoepidemiological study. *Mov Disord*. 2006;21(8):1221-1225. doi:10.1002/mds.20907
19. Bronskill SE, Maclagan LC, Maxwell CJ, et al. Trends in Health Service Use for Canadian Adults With Dementia and Parkinson Disease During the First Wave of the COVID-19 Pandemic. *JAMA Health Forum*. Jan 2022;3(1):e214599. doi:10.1001/jamahealthforum.2021.4599
20. Bu´řill J, Bu´řilova´ P, Pokorna´ A, Kova´čova´ I, Bala´zˇ M. Representation of Parkinson's disease and atypical Parkinson's syndromes in the Czech Republic-A nationwide retrospective study. *PLoS One*. 2021;16(2):e0246342. doi:10.1371/journal.pone.0246342

© 2026 Esper CD et al. *JAMA Network Open*.

21. Butt DA, Tu K, Young J, et al. A validation study of administrative data algorithms to identify patients with Parkinsonism with prevalence and incidence trends. Research Support, Non-U.S. Gov't Validation Studies. *Neuroepidemiology*. 2014;43(1):28-37. doi:10.1159/000365590
22. Callaghan RC, Cunningham JK, Sykes J, Kish SJ. Increased risk of Parkinson's disease in individuals hospitalized with conditions related to the use of methamphetamine or other amphetamine-type drugs. *Drug Alcohol Depend*. Jan 1 2012;120(1-3):35-40. doi:10.1016/j.drugalcdep.2011.06.013
23. Camacho-Soto A, Gross A, Nielsen SS, Dey N, Racette BA. Inflammatory bowel disease and risk of Parkinson's disease in Medicare beneficiaries. *Parkinsonism Relat Disord*. May 2018;50:23-28. doi:10.1016/j.parkreldis.2018.02.008
24. Carriere N, Verloop D, Dupont C, et al. Descriptive study of the parkinsonian population in the north of France: Epidemiological analysis and healthcare consumption. *Revue Neurologique*. Jun 2017;173(6):396-405. doi:10.1016/j.neurol.2017.03.036
25. Cepeda MS, Kern DM, Seabrook GR, Lovestone S. Comprehensive Real-World Assessment of Marketed Medications to Guide Parkinson's Drug Discovery. *Clinical Drug Investigation*. Jul 20 2019;39(11):1067-1075. doi:10.1007/s40261-019-00830-4
26. Chandler JM, Nair R, Biglan K, et al. Characteristics of Parkinson’s Disease in Patients with and without Cognitive Impairment. *Journal of Parkinson's Disease*. 2021;11(3):1381-1392. doi:10.3233/jpd-202190
27. Chang YP, Yang CY, Hu KF, et al. Risk factors for pneumonia among patients with Parkinson's disease: a Taiwan nationwide population-based study. *Neuropsychiatr Dis Treat*. 2016;12:1037-1046. doi:10.2147/NDT.S99365
28. Chekani F, Bali V, Aparasu RR. Quality of life of patients with Parkinson's disease and neurodegenerative dementia: A nationally representative study. *Res Social Adm Pharm*. Jul-Aug 2016;12(4):604-613. doi:10.1016/j.sapharm.2015.09.007
29. Chekani F, Holmes HM, Johnson ML, Chen H, Sherer JT, Aparasu RR. Risk of Mortality Associated With Atypical Antipsychotic use: A National Cohort Study of Older Adults With Depression and Parkinson's Disease. *American Journal of Geriatric Psychiatry*. 2020;28(10):1079-1088. doi:10.1016/j.jagp.2020.01.193
30. Chen YY, Cheng PY, Wu SL, Lai CH. Parkinson's disease and risk of hip fracture: an 8-year follow-up study in Taiwan. *Parkinsonism & Related Disorders*. Jun 2012;18(5):506-509. doi:doi:10.1016/j.parkreldis.2012.01.014
31. Chen JC, Tsai TY, Li CY, Hwang JH. Obstructive sleep apnea and risk of Parkinson's disease: a population-based cohort study. *J Sleep Res*. Mar 22 2015;24(4):432-437. doi:10.1111/jsr.12289
32. Chen CY, Hung HJ, Chang KH, et al. Long-term exposure to air pollution and the incidence of Parkinson's disease: A nested case-control study. *PLoS ONE*. Aug 15 2017;12(8):e0182834. doi:10.1371/journal.pone.0182834
33. Chen H, Kwong JC, Copes R, et al. Living near major roads and the incidence of dementia, Parkinson's disease, and multiple sclerosis: a population-based cohort study. Research Support, Non-U.S. Gov't. *The Lancet*. 2017;389(10070):718-726. doi:10.1016/S0140-6736(16)32399-6
34. Chen CK, Huang JY, Wu YT, Chang YC. Dental Scaling Decreases the Risk of Parkinson's Disease: A Nationwide Population-Based Nested Case-Control Study. *Int J Environ Res Public Health*. Jul 26 2018;15(8):1587. doi:10.3390/ijerph15081587
35. Chen SF, Yang YC, Hsu CY, Shen YC. Risk of Parkinson's disease in patients with hypothyroidism: A nationwide population-based cohort study. *Parkinsonism Relat Disord*. 2020;74:28-32. doi:10.1016/j.parkreldis.2020.04.001
36. Chen W, Sadatsafavi M, Tavakoli H, Samii A, Etminan M. Effects of beta2-Adrenergic Agonists on Risk of Parkinson's Disease in COPD: A Population-Based Study. *Pharmacotherapy*. May 2020;40(5):408-415. doi:10.1002/phar.2383
37. Chen PC, Chung CC, Cheng YY, et al. Retinal Diseases and Parkinson Disease: A Population-Based Study. *Front Neurosci*. 2021;15doi:10.3389/fnins.2021.679092
38. Chillag-Talmor O, Giladi N, Linn S, et al. Use of a refined drug tracer algorithm to estimate prevalence and incidence of Parkinson's disease in a large israeli population. Research Support, Non-U.S. Gov't. *J Parkinsons Dis*. 2011;1(1):35-47. doi:10.3233/JPD-2011-11024
39. Choi HG, Lim JS, Lee YK, Sim S, Kim M. Mortality and cause of death in South Korean patients with Parkinson's disease: a longitudinal follow-up study using a national sample cohort. *BMJ Open*. 2019;9(9):e029776. doi:10.1136/ bmjopen-2019-029776
40. Chou PS, Lai CL, Chou YH, Chang WP. Sleep apnea and the subsequent risk of Parkinson's disease: a 3-year nationwide population-based study. *Neuropsychiatr Dis Treat*. Mar 30 2017;13:959-965. doi:10.2147/NDT.S134311
41. Connolly JG, Bykov K, Gagne JJ. Thiazolidinediones and Parkinson Disease: A Cohort Study. *American Journal of Epidemiology*. Oct 22 2015;182(11):936-944. doi:10.1093/aje/kwv109

© 2026 Esper CD et al. *JAMA Network Open*.

42. Cortese M, Riise T, Engeland A, Ascherio A, Bjornevik K. Urate and the risk of Parkinson's disease in men and women. *Parkinsonism & Related Disorders*. Jul 2018;52:76-82. doi:10.1016/j.parkreldis.2018.03.026
43. Crispo JA, Fortin Y, Thibault DP, et al. Trends in inpatient antiparkinson drug use in the USA, 2001-2012. *Eur J Clin Pharmacol*. 2015;71:1011-1019. doi:10.1007/s00228-015-1881-4
44. Crispo JAG, Willis AW, Thibault DP, et al. Associations between Anticholinergic Burden and Adverse Health Outcomes in Parkinson Disease. *PLoS One*. Mar 3 2016;11(3):e0150621. doi:10.1371/journal.pone.0150621
45. Crispo JAG, Lam M, Le B, et al. Disparities in Deep Brain Stimulation Use for Parkinson's Disease in Ontario, Canada. *Canadian Journal of Neurological Sciences / Journal Canadien des Sciences Neurologiques*. Sep 2020;47(5):642-655. doi:10.1017/cjn.2020.79
46. Dahodwala N, Siderowf A, Xie M, Noll E, Stern M, Mandell DS. Racial differences in the diagnosis of Parkinson's disease. *Mov Disord*. 2009;24(8):1200-1205. doi:10.1002/mds.22557
47. Dahodwala N, Willis AW, Li P, Doshi JA. Prevalence and Correlates of Anti-Parkinson Drug Use in a Nationally Representative Sample. *Movement Disorders Clinical Practice*. Aug 22 2016;4(3):335-341. doi:10.1002/mdc3.12422
48. Dahodwala N, Pettit AR, Jahnke J, et al. Use of a medication-based algorithm to identify advanced Parkinson's disease in administrative claims data: Associations with claims-based indicators of disease severity. *Clin Park Relat Disord*. Feb 26 2020;3doi:10.1016/j.prdoa.2020.100046
49. Dammertz L, Schrag A, Bohlken J, et al. Falling incidence of Parkinson's disease in Germany. *European Journal of Neurology*. Oct 2023;30(10):3124-3131. doi:10.1111/ene.16000
50. Danila O, Hirdes JP, Maxwell CJ, et al. Prevalence of neurological conditions across the continuum of care based on interRAI assessments. *BMC Health Services Research*. 2014;14(29)doi:10.1186/1472-6963-14-29
51. De Vera M, Rahman MM, Rankin J, Kopec J, Gao X, Choi H. Gout and the risk of Parkinson's disease: a cohort study. *Arthritis Rheum*. Nov 15 2008;59(11):1549-1554. doi:10.1002/art.24193
52. DeMarco EC, Zhang Z, Robinson H, Hinyard L. Anxiety in Parkinson's Patients: What's Timing Got to Do with It? *Journal of Geriatric Psychiatry and Neurology*. Nov 2023;36(6):496-504. doi:10.1177/08919887231163293
53. Doblhammer G, Barth A. Prevalence of Morbidity at Extreme Old Age in Germany: An Observational Study Using Health Claims Data. *J Am Geriatr Soc*. Jul 2018;66(7):1262-1268. doi:10.1111/jgs.15460
54. Dore DD, Trivedi AN, Mor V, Friedman JH, Lapane KL. Atypical antipsychotic use and risk of fracture in persons with Parkinsonism. *Mov Disord*. Oct 15 2009;24(13):1941-1948. doi:10.1002/mds.22679
55. Etminan M, Carleton BC, Samii A. Non-steroidal anti-inflammatory drug use and the risk of Parkinson disease: a retrospective cohort study. *Journal of Clinical Neuroscience*. May 2008;15(5):576-577. doi:10.1016/j.jocn.2007.02.095
56. Eusebi P, Franchini D, De Giorgi M, et al. Incidence and prevalence of Parkinson's disease in the Italian region of Umbria: a population-based study using healthcare administrative databases. *Neurological Sciences*. 2019;40(8):1709-1712. doi:10.1007/s10072-019-03872-w
57. Fan J, Searles Nielsen S, Faust IM, Racette BA. Transplant and risk of Parkinson disease. *Parkinsonism & Related Disorders*. 2019;63:149-155. doi:10.1016/j.parkreldis.2019.02.013
58. Fang TC, Wu YH, Chen YH, Lin CH, Chang MH. Risk of Dementia in Patients with Depression or Parkinson's Disease: A Retrospective Cohort Study. *Parkinsons Dis*. 2020;2020(1):8493916. doi:10.1155/2020/8493916
59. Faust IM, Racette BA, Nielsen SS. Validation of a Parkinson Disease Predictive Model in a Population-Based Study. *Parkinsons Dis*. 2020;2020:2857608. doi:10.1155/2020/2857608
60. Feldman AL, Johansson ALV, Nise G, Gatz M, Pedersen NL, Wirdefeldt K. Occupational exposure in parkinsonian disorders: a 43-year prospective cohort study in men. *Parkinsonism Relat Disord*. Nov 2011;17(9):677-682. doi:10.1016/j.parkreldis.2011.06.009
61. Feldman AL, Johansson ALV, Gatz M, et al. Accuracy and sensitivity of Parkinsonian disorder diagnoses in two Swedish national health registers. *Neuroepidemiology*. 2012;38(3):186-193. doi:10.1159/000336356
62. Finkelstein MM, Jerrett M. A study of the relationships between Parkinson's disease and markers of traffic-derived and environmental manganese air pollution in two Canadian cities. *Environmental Research*. 2007;104(3):420-432. doi:10.1016/j.envres.2007.03.002
63. François C, Biaggioni I, Shibao C, et al. Fall-related healthcare use and costs in neurogenic orthostatic hypotension with Parkinson's disease. *Journal of Medical Economics*. 2017;20(5):525-532. doi:10.1080/13696998.2017.1284668
64. Freedman DM, Wu J, Chen H, et al. Associations between cancer and Parkinson's disease in U.S. elderly adults. *International Journal of Epidemiology*. Mar 17 2016;45(3):741-751. doi:10.1093/ije/dyw016

© 2026 Esper CD et al. *JAMA Network Open*.

65. Fullard ME, Thibault DP, Todaro V, et al. Sex disparities in health and health care utilization after Parkinson diagnosis: Rethinking PD associated disability. *Parkinsonism Relat Disord*. Mar 2018;48:45-50. doi:10.1016/j.parkreldis.2017.12.012
66. Gandhi AB, Onukwugha E, Albarmawi H, et al. Health Care Resource Utilization Associated With Parkinson Disease Among Medicare Beneficiaries. *Neurology*. Aug 10 2021;97(6):e597-e607. doi:10.1212/WNL.0000000000012290
67. Gordon PH, Mehal JM, Holman RC, Rowland AS, Cheek JE. Parkinson's disease among American Indians and Alaska natives: a nationwide prevalence study. *Movement Disorders*. Aug 14 2012;27(11):1456-1459. doi:10.1002/mds.25153
68. Gordon PH, Zhao H, Bartley D, et al. Prevalence of Parkinson disease among the Navajo: a preliminary examination. *Journal of Parkinson's Disease*. 2013;3(2):193-198. doi:10.3233/JPD-120158
69. Gordon PH, Mehal JM, Holman RC, Bartholomew ML, Cheek JE, Rowland AS. Incidence and prevalence of Parkinson's disease among Navajo people living in the Navajo nation. *Movement Disorders*. Feb 04 2015;30(5):714-720. doi:10.1002/mds.26147
70. Guo YJ, Liao YC, Lin CH, Chang MH. Initial Medication in Patients of Newly Diagnosed Parkinson's Disease in Taiwan. *PLoS ONE*. Sep 15 2014;9(9):e107465. doi:10.1371/journal.pone.0107465
71. Guttman M, Slaughter PM, Theriault ME, DeBoer DP, Naylor CD. Burden of Parkonsonism: A Population-Based Study. *Mov Disord*. 2003;18(3):313-319. doi:10.1002/mds.10333
72. Han S, Kim S, Kim H, Shin HW, Na KS, Suh HS. Prevalence and incidence of Parkinson's disease and drug-induced parkinsonism in Korea. *BMC Public Health*. Oct 22 2019;19:1328. doi:10.1186/s12889-019-7664-6
73. Heinzel S, Berg D, Binder S, et al. Do We Need to Rethink the Epidemiology and Healthcare Utilization of Parkinson's Disease in Germany? *Front Neurol*. Jun 28 2018;9doi:10.3389/fneur.2018.00500
74. Hernán MA, Logroscino G, García Rodriguez LA. Nonsteroidal anti-inflammatory drugs and the incidence of Parkinson disease. *Neurology*. 2006;66(7):1097-1099. doi:10.1212/01.wnl.0000204446.82823.28
75. Hill EJ, Sharma J, Wissel B, et al. Parkinson's disease diagnosis codes are insufficiently accurate for electronic health record research and differ by race. *Parkinsonism & Related Disorders*. Sep 2023;114:105764. doi:10.1016/j.parkreldis.2023.105764
76. Holt RJ, Sklar AR, Darkow T, Goldberg GA, Johnson JC, Harley CR. Prevalence of Parkinson's disease-induced psychosis in a large U.S. managed care population. *J Neuropsychiatry Clin Neurosci*. 2010;22(1):105-110. doi:10.1176/jnp.2010.22.1.105
77. Horsfall L, Petersen I, Walters K, Schrag A. Time trends in incidence of Parkinson's disease diagnosis in UK primary care. *J Neurol*. Dec 23 2013;260(5):1351-1357. doi:10.1007/s00415-012-6804-z
78. Huse DM, Schulman K, Orsini L, Castelli-Haley J, Kennedy S, Lenhart G. Burden of illness in Parkinson's disease. *Mov Disord*. Nov 2005;20(11):1449-1454. doi:10.1002/mds.20609
79. Iketani R, Imai S, Horiguchi H, Furushima D, Fushimi K, Yamada H. Risk stratification for physical morbidity using factors associated with atypical antipsychotic treatment in Parkinson's disease: A retrospective observational study using administrative claims data. *Journal of Clinical Neuroscience*. Jun 2020;76:189-194. doi:10.1016/j.jocn.2020.04.009
80. Jain S, Himali J, Beiser A, et al. Validation of Secondary Data Sources to Identify Parkinson Disease Against Clinical Diagnostic Criteria. *Am Journal Epidemiol*. Feb 1 2015;181(3):185-190. doi:10.1093/aje/kwu326
81. Jeong SM, Jang W, Shin DW. Association of statin use with Parkinson's disease: Dose–response relationship. *Mov Disord*. 2019;34(7):1014-1021. doi:10.1002/mds.27681
82. Johnson S, Davis M, Kaltenboeck A, et al. Early retirement and income loss in patients with early and advanced Parkinson's disease. *Appl Health Econ Health Policy*. Aug 06 2011;9(6):367-376. doi:10.2165/11596900-000000000-00000
83. Jones CA, Martin WRW, Wieler M, King-Jesso P, Voaklander DC. Incidence and mortality of Parkinson's disease in older Canadians. *Parkinsonism & Related Disorders*. 2012;18(4):327-331. doi:10.1016/j.parkreldis.2011.11.018
84. Kab S, Spinosi J, Chaperon L, et al. Agricultural activities and the incidence of Parkinson's disease in the general French population. Research Support, Non-U.S. Gov't. *Eur J Epidemiol*. Feb 09 2017;32(3):203-216. doi:10.1007/s10654-017-0229-z
85. Kalilani L, Friesen D, Boudiaf N, Asgharnejad M. The characteristics and treatment patterns of patients with Parkinson's disease in the United States and United Kingdom: A retrospective cohort study. *PLoS One*. Nov 22 2019;14(11):e0225723. doi:10.1371/journal.pone.0225723

© 2026 Esper CD et al. *JAMA Network Open*.

86. Kaltenboeck A, Johnson SJ, Davis MR, et al. Direct costs and survival of medicare beneficiaries with early and advanced Parkinson's disease. *Parkinsonism & Related Disorders*. May 2012;18(4):321-326. doi:10.1016/j.parkreldis.2011.11.015
87. Kasamo S, Takeuchi M, Ikuno M, et al. Real-world pharmacological treatment patterns of patients with young-onset Parkinson's disease in Japan: a medical claims database analysis. *J Neurol*. 2019;266(8):1944-1952. doi:10.1007/s00415-019-09360-7
88. Kim DS, Kunicki ZJ, Philips OW, et al. Racial and geographic disparities with gastrostomy tube placement in dementia and parkinsonian disorders. *Parkinsonism & Related Disorders*. Oct 2021;91:28-31. doi:10.1016/j.parkreldis.2021.08.016
89. Konings B, Villatoro L, Van den Eynde J, et al. Gastrointestinal syndromes preceding a diagnosis of Parkinson's disease: testing Braak's hypothesis using a nationwide database for comparison with Alzheimer's disease and cerebrovascular diseases. *Gut*. Nov 2023;72(11):2103-2111. doi:10.1136/gutjnl-2023-329685
90. Kostev K, Doege C, Jacob L, et al. Association between Antiepileptic Drugs and Incident Parkinson's Disease among Patients Followed in German Primary Care Practices. *Brain Sci*. Mar 6 2023;13(3)doi:10.3390/brainsci13030450
91. Kowal SL, Dall TM, Chakrabarti R, Storm MV, Jain A. The current and projected economic burden of Parkinson's disease in the United States. *Mov Disord*. Mar 2013;28(3):311-318. doi:10.1002/mds.25292
92. Krzyzanowski B, Searles Nielsen S, Turner JR, Racette BA. Fine Particulate Matter and Parkinson Disease Risk Among Medicare Beneficiaries. *Neurology*. Nov 21 2023;101(21):e2058-e2067. doi:10.1212/WNL.0000000000207871
93. Lee PC, Liu LL, Sun Y, et al. Traffic-related air pollution increased the risk of Parkinson's disease in Taiwan: A nationwide study. *Environ Int*. 2016;96:75-81. doi:10.1016/j.envint.2016.08.017
94. Lee SE, Han K, Baek JY, et al. Association Between Diabetic Retinopathy and Parkinson Disease: The Korean National Health Insurance Service Database. *J Clin Endocrinol Metab*. Sep 1 2018;103(9):3231-3238. doi:10.1210/jc.2017-02774
95. Lee SH, Lee SJ, Kim YJ. Region-Based Analysis of Prevalence and Incidence of Parkinson's Disease: Analysis of the National Sample Cohort in South Korea. *J Clin Neurol*. Oct 2018;14(4):478-486. doi:10.3988/jcn.2018.14.4.478
96. Lien WH, Lien WC, Kuan TS, Wu ST, Chen YT, Chiu CJ. Parkinson disease and musculoskeletal pain: an 8-year population-based cohort study. *Pain*. Jul 2017;158(7):1234-1240. doi:10.1097/j.pain.0000000000000904
97. Lin KD, Yang CY, Lee MY, Ho SC, Liu CK, Shin SJ. Statin therapy prevents the onset of Parkinson disease in patients with diabetes. *Ann Neurol*. Oct 2016;80(4):532-540. doi:10.1002/ana.24751
98. Lin FY, Yang YC, Lin CL, Lee LJH. Increased risk of overactive bladder in patients with idiopathic Parkinson's disease: Insight from a nationwide population-based cohort study. *PLoS One*. 2018;13(3):e0193783. doi:10.1371/journal.pone.0193783
99. Lin WY, Lin MS, Weng YH, et al. Association of Antiviral Therapy With Risk of Parkinson Disease in Patients With Chronic Hepatitis C Virus Infection. *JAMA Neurol*. Jun 5 2019;76(9):1019-1027. doi:10.1001/jamaneurol.2019.1368
100. Liu B, Chen H, Fang F, Tillander A, Wirdefeldt K. Early-Life Factors and Risk of Parkinson's Disease: A Register-Based Cohort Study. *PLoS One*. 2016;11(4):e0152841. doi:10.1371/journal.pone.0152841
101. Liu CC, Li CY, Lee PC, Sun Y. Variations in incidence and prevalence of Parkinson's disease in Taiwan: A population-based nationwide study. *Parkinson's Disease*. 2016;2016(8756359)doi:10.1155/2016/8756359
102. Liu WM, Wu RM, Lin JW, Liu YC, Chang CH, Lin CH. Time trends in the prevalence and incidence of Parkinson's disease in Taiwan: A nationwide, population-based study. *Journal of the Formosan Medical Association*. 2016;115(7):531-538. doi:10.1016/j.jfma.2015.05.014
103. Liu G, Sterling NW, Kong L, et al. Statins may facilitate Parkinson's disease: Insight gained from a large, national claims database. *Mov Disord*. 2017;32(6):913-917. doi:10.1002/mds.27006
104. Lix LM, Hobson DE, Azimaee M, Leslie WD, Burchill C, Hobson S. Socioeconomic variations in the prevalence and incidence of Parkinson's disease: a population-based analysis. Research Support, Non-U.S. Gov't. *Journal of Epidemiology & Community Health*. 2010;64(4):335-340. doi:10.1136/jech.2008.084954
105. Lusk JB, Choi S, Clark AG, et al. Dementia and Parkinson's disease diagnoses in electronic health records vs. Medicare claims data: a study of 101,980 linked patients. *BMC Neurology*. Sep 12 2023;23(1):325. doi:10.1186/s12883-023-03361-w
106. Maclagan LC, Marras C, Sewell IJ, et al. Trends in health service use among persons with Parkinson's disease by rurality: A population-based repeated cross-sectional study. *PLoS One*. 2023;18(5):e0285585. doi:10.1371/journal.pone.0285585

© 2026 Esper CD et al. *JAMA Network Open*.

107. Madubata CC, Olsen MA, Stwalley DL, Gutmann DH, Johnson KJ. Neurofibromatosis type 1 and chronic neurological conditions in the United States: an administrative claims analysis. *Genetics in Medicine*. 2015;17(1):36-42. doi:10.1038/gim.2014.70
108. Mantri S, Fullard ME, Beck J, Willis AW. State-level prevalence, health service use, and spending vary widely among Medicare beneficiaries with Parkinson disease. *NPJ Parkinsons Dis*. Jan 2019;5:1. doi:10.1038/s41531-019-0074-8
109. Maxwell CJ, Maclagan LC, Harris DA, et al. Incidence of neurological and psychiatric comorbidity over time: a population-based cohort study in Ontario, Canada. *Age and ageing*. Feb 2 2022;51(2)doi:10.1093/ageing/afab277
110. Moisan F, Gourlet V, Mazurie JL, et al. Prediction Model of Parkinson's Disease Based on Antiparkinsonian Drug Claims. *American Journal of Epidemiology*. Aug 1 2011;174(3):354-363. doi:10.1093/aje/kwr081
111. Moisan F, Kab S, Mohamed F, et al. Parkinson disease male-to-female ratios increase with age: French nationwide study and meta-analysis. *J Neurol Neurosurg Psychiatry*. Sep 2016;87(9):952-957. doi:10.1136/jnnp-2015-312283
112. Muzerengi S, Rick C, Begaj I, et al. Coding accuracy for Parkinson's disease hospital admissions: implications for healthcare planning in the UK. *Public Health*. 2017;146:4-9. doi:10.1016/j.puhe.2016.12.024
113. Nerius M, Fink A, Doblhammer G. Parkinson's disease in Germany: prevalence and incidence based on health claims data. *Acta Neurol Scand*. Nov 2017;136(5):386-392. doi:10.1111/ane.12694
114. Noyes K, Liu H, Holloway R, Dick AW. Accuracy of Medicare claims data in identifying Parkinsonism cases: comparison with the Medicare current beneficiary survey. *Mov Disord*. 2007;22(4):509-514. doi:10.1002/mds.21299
115. Okunoye O, Horsfall L, Marston L, Walters K, Schrag A. Mortality of People with Parkinson's Disease in a Large UK-Based Cohort Study: Time Trends and Relationship to Disease Duration. *Mov Disord*. Dec 2021;36(12):2811-2820. doi:10.1002/mds.28727
116. Okunoye O, Marston L, Walters K, Schrag A. Change in the incidence of Parkinson's disease in a large UK primary care database. *npj Parkinsons Dis*. Mar 15 2022;8:23. doi:10.1038/s41531-022-00284-0
117. Orayj K. Impact of Antidepressants on Cardiac Events and All-Cause Mortality in Parkinson's Disease: A National Data-Linkage Study. *Neuropsychiatr Dis Treat*. 2021;17:2499-2510. doi:10.2147/NDT.S325521
118. Pearson C, Hartzman A, Munevar D, et al. Care access and utilization among medicare beneficiaries living with Parkinson's disease. *NPJ Parkinsons Dis*. Jul 10 2023;9:108. doi:10.1038/s41531-023-00523-y
119. Peterson BJ, Rocca WA, Bower JH, Savica R, Mielke MM. Identifying incident Parkinson's disease using administrative diagnostic codes: a validation study. *Clinical Parkinsonism & Related Disorders*. 2020;3:100061. doi:10.1016/j.prdoa.2020.100061
120. Pou MA, Orfila F, Pagonabarraga J, Ferrer-Moret S, Corominas H, Diaz-Torne C. Risk of Parkinson's disease in a gout Mediterranean population: A case-control study. *Joint Bone Spine*. 2022;89(6)doi:10.1016/j.jbspin.2022.105402
121. Prada SI, Pérez AM, Valderrama-Chaparro J, Molina-Echeverry MI, Orozco JL, Takeuchi Y. Direct cost of Parkinson's disease in a health system with high judicialization: evidence from Colombia. *Expert Review of Pharmacoeconomics & Outcomes Research*. 2019;20(6):587-593. doi:10.1080/14737167.2020.1681266
122. Pupillo E, Cricelli C, Mazzoleni F, et al. Epidemiology of Parkinson's Disease: A Population-Based Study in Primary Care in Italy. *Neuroepidemiology*. 2016;47(1):38-45. doi:10.1159/000448402
123. Richy FF, Pietri G, Moran KA, Senior E, Makaroff LE. Compliance with pharmacotherapy and direct healthcare costs in patients with Parkinson's disease: a retrospective claims database analysis. *Appl Health Econ Health Policy*. 2013;11:395-406. doi:10.1007/s40258-013-0033-1
124. Riedel O, Bitters D, Amann U, Garbe E, Langner I. Estimating the prevalence of Parkinson's disease (PD) and proportions of patients with associated dementia and depression among the older adults based on secondary claims data. Research Support, Non-U.S. Gov't. *International Journal of Geriatric Psychiatry*. 2016;31(8):938-943. doi:10.1002/gps.4414
125. Schmitz S, Vaillant M, Renoux C, et al. Prevalence and Cost of Care for Parkinson's Disease in Luxembourg: An Analysis of National Healthcare Insurance Data. *PharmacoEconomics Open*. May 2022;6:405-414. doi:10.1007/s41669-021-00321-3
126. Schrag A, Horsfall L, Walters K, Noyce A, Petersen I. Prediagnostic presentations of Parkinson's disease in primary care: a case-control study. *The Lancet Neurology*. Jan 2015;14(1):57-64. doi:10.1016/S1474-4422(14)70287-X
127. Scott GD, Neilson LE, Woltjer R, Quinn JF, Lim MM. Lifelong Association of Disorders Related to Military Trauma with Subsequent Parkinson's Disease. *Mov Disord*. Aug 2023;38(8):1483-1492. doi:10.1002/mds.29457

© 2026 Esper CD et al. *JAMA Network Open*.

128. Searles Nielsen S, Warden MN, Camacho-Soto A, Willis AW, Wright BA, Racette BA. A predictive model to identify Parkinson disease from administrative claims data. *Neurology*. Sep 1 2017;89:1448-1456. doi:10.1212/WNL.0000000000004536
129. Seki M, Kawata Y, Hayashi A, Arai M, Fujimoto S. Prescribing patterns and determinants for elderly patients with Parkinson's disease in Japan: a retrospective observational study using insurance claims databases. *Frontiers in Neurology*. 2023;14:1162016. doi:10.3389/fneur.2023.1162016
130. Seo HG, Byun SJ, Oh BM, Park SJ. Ten-Year Relative Survival From the Diagnosis of Parkinson's Disease: A Nationwide Database Study. *J Am Med Dir Assoc*. Aug 2021;22(8):1757-1761. doi:10.1016/j.jamda.2020.11.021
131. Shin S, Burnett RT, Kwong JC, et al. Effects of ambient air pollution on incident Parkinson's disease in Ontario, 2001 to 2013: a population-based cohort study. *International Journal of Epidemiology*. Dec 1 2018;47(6):2038-2048. doi:10.1093/ije/dyy172
132. Song Y, E JY, Guo T, et al. Treatment Patterns and Healthcare Resource Use in Medicare Beneficiaries with Parkinson's Disease. *ClinicoEconomics and Outcomes Research*. 2023;15:631-643. doi:10.2147/CEOR.S422023
133. Straif-Bourgeois S, Ratard R. Parkinson Disease Hospitalizations and Mortality in Louisiana, 1999-2012. *Journal of the Louisiana State Medical Society*. 2015;167(6):248-251.
134. Swarztrauber K, Anau J, Peters D. Identifying and distinguishing cases of parkinsonism and Parkinson's disease using ICD-9 CM codes and pharmacy data. *Mov Disord*. Aug 2005;20(8):964-970. doi:10.1002/mds.20479
135. Swarztrauber K, Koudelka C, Brodsky MA. Initial pharmacotherapy in a population of veterans with Parkinson disease. *Neurology*. 2006;66(9):1425-1426. doi:10.1212/01.wnl.0000210433.49727.40
136. Szatmári S, Jr. , Ajtay A, Bálint M, Takáts A, Oberfrank F, Bereczki D. Linking Individual Patient Data to Estimate Incidence and Prevalence of Parkinson's Disease by Comparing Reports of Neurological Services and Pharmacy Prescription Refills at a Nationwide Level. *Front Neurol*. 2019;10:640. doi:10.3389/fneur.2019.00640
137. Szumski NR, Cheng EM. Optimizing algorithms to identify Parkinson's disease cases within an administrative database. *Mov Disord*. Jan 15 2009;24(1):51-56. doi:10.1002/mds.22283
138. Thacker T, Wegele AR, Pirio Richardson S. Utility of electronic medical record for recruitment in clinical research: from rare to common disease. *Mov Disord Clin Pract*. 2016;3(5):507-509. doi:10.1002/mdc3.12318
139. Ton TG, Jain S, Boudreau R, et al. Post Hoc Parkinson's Disease: Identifying an Uncommon Disease in the Cardiovascular Health Study. *Neuroepidemiology*. 2010;35(4):241-249. doi:10.1159/000319895
140. Valent F, Devigili G, Rinaldo S, Del Zotto S, Tullio A, Eleopra R. The epidemiology of Parkinson's disease in the Italian region Friuli Venezia Giulia: a population-based study with administrative data. *Neurological Sciences*. Apr 2018;39(4):699-704. doi:10.1007/s10072-018-3273-x
141. van de Vijver DAMC, Roos RAC, Jansen PAF, Porsius AJ, de Boer A. Estimation of incidence and prevalence of Parkinson's disease in the elderly using pharmacy records. Research Support, Non-U.S. Gov't. *Pharmacoepidemiol Drug Saf*. 2001;10(6):549-554. doi:10.1002/pds.624
142. Van Den Eeden SK, Tanner CM, Bernstein AL, et al. Incidence of Parkinson's disease: variation by age, gender, and race/ethnicity. Research Support, U.S. Gov't, P.H.S. *Am J Epidemiol*. Jun 1 2003;157(11):1015-1022. doi:10.1093/aje/kwg068
143. Vlaar T, Kab S, Schwaab Y, Frery N, Elbaz A, Moisan F. Association of Parkinson's disease with industry sectors: a French nationwide incidence study. *Eur J Epidemiol*. Nov 2018;33(11):1101-1111. doi:10.1007/s10654-018-0399-3
144. Wada - Isoe K, Tsuboi Y, Kondo H, Kojima Y, Takeshima T, Iwasaki K. Non - ergot dopamine agonist therapy for Parkinson's disease in Japan: A claims database analysis. *Neurology and Clinical Neuroscience*. 2023;11(2):63-71. doi:10.1111/ncn3.12689
145. Wang MT, Lian PW, Yeh CB, Yen CH, Ma KH, Chan AL. Incidence, prescription patterns, and determinants of antipsychotic use in patients with Parkinson's disease. *Mov Disord*. Aug 1 2011;26(9):1663-1669. doi:10.1002/mds.23719
146. Wei YJ, Palumbo FB, Simoni-Wastila L, et al. Antiparkinson drug use and adherence in medicare part D beneficiaries with Parkinson's disease. *Clin Ther*. Oct 2013;35(10):1513-1525.e1. doi:10.1016/j.clinthera.2013.09.001
147. Wei YJ, Palumbo FB, Simoni-Wastila L, et al. Relationships between antiparkinson medication nonadherence, regimen modifications, and healthcare utilization and expenditures. *Parkinsonism & Related Disorders*. 2015;21(1):36-41. doi:10.1016/j.parkreldis.2014.10.021
148. Wei WQ, Teixeira PL, Mo H, Cronin RM, Warner JL, Denny JC. Combining billing codes, clinical notes, and medications from electronic health records provides superior phenotyping performance. *J Am Med Inform Assoc*. Apr 2016;23(e1):e20-e27. doi:10.1093/jamia/ocv130

© 2026 Esper CD et al. *JAMA Network Open*.

149. Weimers P, Halfvarson J, Sachs MC, et al. Inflammatory Bowel Disease and Parkinson's Disease: A Nationwide Swedish Cohort Study. *Inflamm Bowel Dis*. Jan 1 2019;25(1):111-123. doi:10.1093/ibd/izy190
150. Weintraub D, Chiang C, Kim HM, et al. Association of Antipsychotic Use With Mortality Risk in Patients With Parkinson Disease. *JAMA Neurology*. 2016;73(5):535-541. doi:10.1001/jamaneurol.2016.0031
151. Weir S, Samnaliev M, Kuo TC, et al. Short- and long-term cost and utilization of health care resources in Parkinson's disease in the UK. *Mov Disord*. Jul 2018;33(6):974-981. doi:10.1002/mds.27302
152. Wetmore JB, Li S, Yan H, et al. Increases in institutionalization, healthcare resource utilization, and mortality risk associated with Parkinson disease psychosis: Retrospective cohort study. *Parkinsonism Relat Disord*. Nov 2019;68:95-101. doi:10.1016/j.parkreldis.2019.10.018
153. White D, Moore S, Waring S, Cook K, Lai E. Identifying incident cases of parkinsonism among veterans using a tertiary medical center. *Mov Disord*. May 15 2007;22(7):915-923. doi:10.1002/mds.21353
154. Williamson T, Green ME, Birtwhistle R, et al. Validating the 8 CPCSSN Case Definitions for Chronic Disease Surveillance in a Primary Care Database of Electronic Health Records. *Ann Fam Med*. 2014;12(4):367-372. doi:10.1370/afm.1644
155. Willis AW, Evanoff BA, Lian M, Criswell SR, Racette BA. Geographic and ethnic variation in Parkinson disease: a population-based study of US Medicare beneficiaries. *Neuroepidemiology*. 2010;34(3):143-151. doi:10.1159/000275491
156. Willis AW, Schootman M, Evanoff BA, Perlmutter JS, Racette BA. Neurologist care in Parkinson disease: A utilization, outcomes, and survival study. *Neurology*. Aug 30 2011;77(9):851-857. doi:10.1212/WNL.0b013e31822c9123
157. Xu S, Li W, Di Q. Association of Dietary Patterns with Parkinson's Disease: A Cross-Sectional Study Based on the United States National Health and Nutritional Examination Survey Database. *Eur Neurol*. 2023;86(1):63-72. doi:10.1159/000527537
158. Yang YW, Hsieh TF, Li CI, et al. Increased risk of Parkinson disease with diabetes mellitus in a population-based study. *Medicine (Baltimore)*. Jan 2017;96(3):e5921. doi:10.1097/MD.0000000000005921
159. Yuchi W, Sbihi H, Davies H, Tamburic L, Brauer M. Road proximity, air pollution, noise, green space and neurologic disease incidence: a population-based cohort study. *Environ Health*. Jan 21 2020;19:8. doi:10.1186/s12940-020-0565-4
160. Zenesini C, Belotti LMB, Baccari F, et al. Validation of Administrative Health Data Algorithms for Identifying Persons with Parkinson's Disease and the 10-Year Prevalence Trend in Bologna, Italy. *Neuroepidemiology*. 2023;57(5):336-344. doi:10.1159/000533362
161. SNOMED International. Mapping Tool. Accessed 10/15/22, 2022. https://mapping.ihtsdotools.org/#/project/records?refSetId=P447562003
162. World Health Organization. ICD-10 Version: 2019. Accessed 10/15/22, 2022. https://icd.who.int/browse10/2019/en#/G20-G26
163. Tolosa E, Garrido A, Scholz SW, Poewe W. Challenges in the diagnosis of Parkinson's disease. *Lancet Neurol*. 2021;20(5):385-397. doi:10.1016/s1474-4422(21)00030-2
164. Armstrong MJ, Okun MS. Diagnosis and Treatment of Parkinson Disease: A Review. *JAMA*. Feb 11 2020;323(6):548-560. doi:10.1001/jama.2019.22360
165. Bloem BR, Okun MS, Klein C. Parkinson's disease. *Lancet*. Jun 12 2021;397(10291):2284-2303. doi:10.1016/S0140-6736(21)00218-X
166. Mehanna R, Jankovic J. Young-onset Parkinson's disease: Its unique features and their impact on quality of life. *Parkinsonism Relat Disord*. Aug 2019;65:39-48. doi:10.1016/j.parkreldis.2019.06.001
167. Post B, van den Heuvel L, van Prooije T, van Ruissen X, van de Warrenburg B, Nonnekes J. Young Onset Parkinson's Disease: A Modern and Tailored Approach. *J Parkinsons Dis*. 2020;10(s1):S29-S36. doi:10.3233/JPD-202135
168. Golbe LI. Young-onset Parkinson's Disease: A clinical review. *Neurology*. Feb 1991;41(2)(1)doi:10.1212/WNL.41.2_Part_1.168
169. Mehanna R, Moore S, Hou JG, Sarwar AI, Lai EC. Comparing clinical features of young onset, middle onset and late onset of Parkinson's disease. *Parkinsonism & Related Disorders*. May 2014;20(5):530-534. doi:10.1016/j.parkreldis.2014.02.013
170. Mehanna R, Smilowska K, Fleisher J, et al. Age Cutoff for Early-Onset Parkinson's Disease: Recommendations from the International Parkinson and Movement Disorder Society Task Force on Early Onset Parkinson's Disease. *Mov Disord Clin Pract*. 2022;9(7):869-878. doi:10.1002/mdc3.13523

© 2026 Esper CD et al. *JAMA Network Open*.

171. Te Groen M, Bloem BR, Wu SS, Post B, Parkinson's Foundation Quality Improvement Initiative. Better quality of life and less caregiver strain in young-onset Parkinson's disease: a multicentre retrospective cohort study. *J Neurol*. Mar 2021;268(3):1102-1109. doi:10.1007/s00415-020-10266-y

172. Association APD. Medications for Parkinson's. Accessed 10/15, 2022. https://www.apdaparkinson.org/what-is-parkinsons/treatment-medication/medication/

© 2026 Esper CD et al. *JAMA Network Open*.
