# Supplementary material for: Case Definitions and Data Sources for NNCSS Parkinson Disease Surveillance: A Systematic Review
Source: JAMA Netw Open. 2026 Jun 8;9(6):e2613928. doi: 10.1001/jamanetworkopen.2026.13928 (PMC13247814; doi:10.1001/jamanetworkopen.2026.13928)
Supplement: Supplement 2. — Data Sharing Statement [file jamanetwopen-e2613928-s002.pdf]

Data Sharing Statement

Esper. Case Definitions and Data Sources for NNCSS Parkinson Disease Surveillance. *JAMA Netw Open*. Published May 28, 2026. doi:10.1001/jamanetworkopen.2026.13928

Data
Data available: No
